# Synthesis and Evaluation of Novel 2,2-Dimethylthiochromanones as Anti-Leishmanial Agents

**DOI:** 10.3390/molecules26082209

**Published:** 2021-04-12

**Authors:** Seán Coll, Mohammad Alhazmi, Patrícia de Aguiar Amaral, Sandra Bourgeade-Delmas, Anne-Cécile Le Lamer, James W. Barlow

**Affiliations:** 1 Department of Chemistry, RCSI University of Medicine and Health Sciences, 123 St. Stephen’s Green, Dublin 2, Ireland; seancoll@rcsi.ie (S.C.); malhazmi@rcsi.ie (M.A.); 2 Laboratory of Medicinal Plants (LaPlaM/PPGCA), Universidade do Extremo Sul Catarinense (UNESC), Avenida Universitária 1105, Bairro Universitário, Criciúma 88806-000, SC, Brazil; amaral@unesc.net; 3 UMR 152 IRD-UPS PHARMADEV, Faculté des Sciences Pharmaceutiques, Université Toulouse III-Paul Sabatier, 35 Chemin des Maraîchers, 31062 Toulouse CEDEX 09, France; sandra.bourgeade-delmas@ird.fr (S.B.-D.); anne-cecile.le-lamer@univ-tlse3.fr (A.-C.L.L.)

**Keywords:** thiochromanone, uniflorol, gibbilimbol, *Leishmania*

## Abstract

Within this work, we describe the design and synthesis of a range of novel thiochromanones based on natural products reported to possess anti-leishmanial action, and their synthetic derivatives. All compounds were elaborated via the key intermediate 2,2,6-trimethoxythiochromanone, which was modified at the benzylic position to afford various ester, amine and amide analogues, substituted by chains of varying lipophilicity. Upon testing in *Leishmania*, IC_50_ values revealed the most potent compounds to be phenylalkenyl and haloalkyl amides **11a** and **11e**, with IC_50_ values of 10.5 and 7.2 μM, respectively.

## 1. Introduction

**Figure 1:**
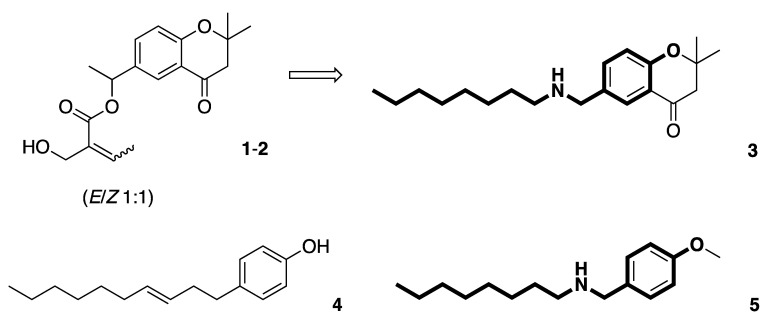
Natural products with activity against *Leishmania* and their synthetic analogues.

Leishmaniasis refers to a spectrum of diseases due to infection with one of a number of protozoal species within the genus *Leishmania*, transmitted through the bite of infected sandflies. Historically, it has been widespread in tropical climates across many continents. In humans, *Leishmania* parasites replicate intracellularly and patients classically present with either visceral or cutaneous disease [1]. Available treatments suffer from disadvantages including toxicity, formulation challenges and a lack of oral dosage forms. Of particular concern, resistance to all common agents, namely pentavalent antimonials, pentamidine, miltefosine, paromomycin and amphotericin B has been documented [2]. Therefore, novel approaches to prevent and treat *Leishmania* require an ongoing research focus. In this context, small molecule therapies remain affordable and druggable approaches, with significant anti-parasitic activity demonstrated for several compounds, encompassing natural products such as oxygenated heterocycles and alkenylphenols, and their synthetic isosteres (Figure 1). Examples include the chromanone uniflorols **1**–**2** of *Calea* spp, which inhibited *L. major* promastigote growth by 55–89% between 25–100 μg/mL [3]. Synthetic derivatization of these compounds with the aim of improving stability and activity resulted in the synthesis of **3**, which inhibited axenic amastigotes and intracellular amastigotes of *L. infantum* with IC_50_ values of 25.3 and 24.6 μM respectively [4]. This compound is notable for possessing a lipophilic aminoalkyl chain *para* to the chromanone oxygen. Considering this structural scaffold, parallels emerge to the observations of Varela et al. [5], who prepared a range of synthetic phenolic derivatives based on the alkenylphenol gibbilimbol B, **4**, a natural product isolated from *Piper malacophyllum* by de Oliveira and colleagues [6], who noted that it possessed leishmanicidal activity. Subsequent papers by Varela and colleagues have expanded the structure-activity relationships around this compound, with the aim of improving selectivity and solubility, while maintaining anti-parasitic activity. Of the various analogues prepared to date, methyl ether **5**, bearing a *para*-octanoyl chain, displayed high activity against amastigotes of both *L. infantum* and *Trypanosoma cruzi*, with IC_50_ values of 1.3 and 5.8 μM respectively [7].

**Figure 2:**
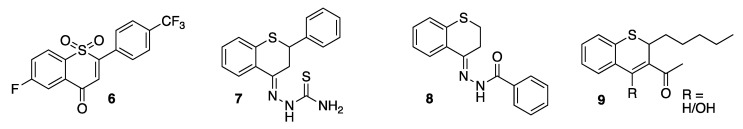
Thiochromanones with activity against *Leishmania.*

In addition to these structures, other groups have probed the anti-leishmanial activity of related synthetic heterocycles. One notable example is the thiochromanone class (Figure 2), the sulfur analogues of chromanones such as 1–3. Thiochromanones represent an interesting group from a medicinal chemistry perspective, with examples known to display anti-fungal [8], anti-cancer [9] or anti-trypanosomal [10] activities. In the context of *Leishmania*, certain thiochromanone derivatives containing either semicarbazone, thiosemicarbazone or triazine nitrile warheads were developed as specific cysteine protease B inhibitors [11]. In more recent work, Vargas and colleagues [12] prepared several thiochromanones modified at ring positions 2 and 6. Upon testing against *L. (V) panamensis*, active compounds such as 6 were those bearing a vinyl sulfone moiety and a phenyl moiety at *C*-2, with EC_50_ values < 10 μM and a selectivity index of over 100 for some compounds. Within this series of compounds, activity decreased upon removal of either the double bond or the sulfone moiety. The same group later published further results on the activity of acyl hydrazone derivatives of thiochromanones against the same leishmanial species [13]. Such derivatization significantly enhanced anti-leishmanial activity, with semicarbazone and thiosemicarbazone derivatives of thioflavanone displaying the highest activities, with 7 displaying an EC_50_ value of 5.1 µM, with low cytotoxicity. Thiochroman hydrazides without *C*-2 substitution were separately evaluated by Zapata et al. [14], who studied topical application of 8 co-formulated with saponins, and this combination proved very effective against parasite survival (*L. braziliensis* & *L. pifanoi*) and infectivity. Most recently, Ortiz et al. [15] published an evaluation of the activity of various thiochromenes, thichromanones and hydrazones with *C*-2 or *C*-3 carbonyl or carboxyl substitutions against intracellular amastigotes of *L. (V) panamensis*. A number of compounds, notably of structural type 9, had EC_50_ values below 10 µM, but clear structure-activity relationships were not discerned.

**Figure 3:**
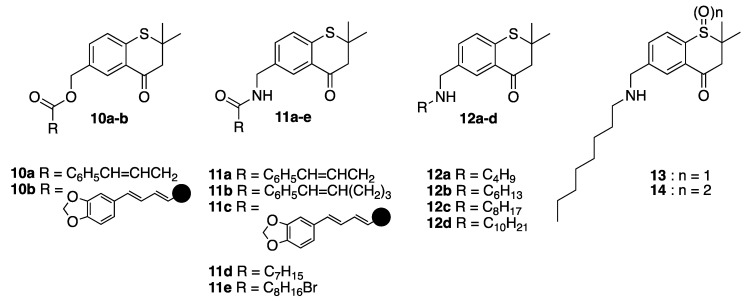
Synthetic targets based on lead compounds **1**–**3**.

Given the interesting activities of the chromanones and thiochromanones described in the literature against *Leishmania* species, we resolved to prepare thio analogues of the known anti-leishmanial chromanones 1–3 (Figure 3). We envisaged ester 10 and amide 11 derivatives, which incorporate either a benzylester or benzylamido linkage *para* to the thiochromanone sulfur. We also sought to vary the amido functionality by replacing it with a more basic alkylamino chain, as seen in 12. These compounds represent both sulfur analogues of chromanone 3 and also derivatives of the potent gibbilimbol ether 5, within a thiochromanone framework. To investigate the impact of variant oxidation states of sulfur, we envisaged compounds 13 and 14.

## 2. Results

### 2.1. Chemistry

**Scheme 1:**
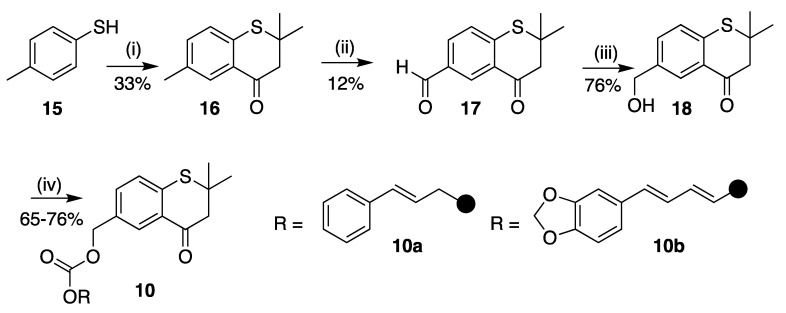
Synthesis of compounds **10a**,**b**. *Reagents and conditions*: (i) (CH_3_)_2_C=CHCOOH, MeSO_3_H, 80 °C, 8 h; (ii) K_2_S_2_O_8_, CuSO_4_, H_2_O/CH_3_CN (1:1), 75–80 °C, 1 h; (iii) *D. carota*, RT, 72 h; (iv) Appropriate acid, EDC, Et_3_N, DCM, 0 °C-RT, 24–72 h.

Construction of thiochromanone skeletons may follow one of a number of approaches, including Pd-catalyzed carbonylative heteroannulation of iodothiophenols with allenes and carbon monoxide, base-catalyzed cyclization of β-halopropanoic acids with arylthiophenols, base-catalyzed condensation of β-propiolactone with 2-ethylthiophenol followed by acid-promoted cyclization, and intramolecular Friedel−Crafts acylation with Lewis acids or methanesulfonic acid [16]. A recently described one-pot procedure employs a microwave-assisted protocol for the synthesis of 2-/3-methylthiochroman-4-ones by superacid-catalyzed alkylation followed by cyclic acylation [17]. However, preparation of the more hindered 2,2-dimethyl-substituted analogues is more challenging than either un- or mono-substituted C-2 analogues, and protocols such as base-catalyzed cyclization afforded very low yields in our preliminary experiments. Attempts at superacid-catalyzed reactions were entirely unsuccessful. Ultimately, we utilised the established Friedel-Crafts heterocyclisation of commercial thiophenol **15** with 3,3-dimethylacrylic acid in methanesulfonic acid [18], to afford thiochromanone **16** (Scheme 1). Oxidation using persulfate afforded the benzylic aldehyde **17**, easily separable from the side-product sulfoxide. Bioreduction using Daucus carota cleanly afforded **18**, which was esterified with appropriate acids using the coupling agent 1-ethyl-3-(3′-dimethylaminopropyl)carbodiimide hydrochloride (EDC) under basic conditions.

**Scheme 2:**
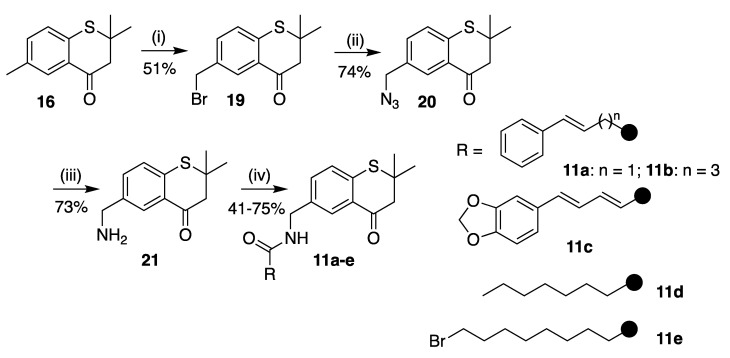
Synthesis of compounds **11a**–**e**. *Reagents and conditions*: (i) NBS, dibenzoyl peroxide, cyclohexane, 95–100 °C, 8 h; (ii) NaN_3_, DMF, 50 °C, 3 h; (iii) PPH_3_, THF/H_2_O (10:1), RT, 12 h; (iv) RCOOH, EDC, Et_3_N, RT, 24 h.

For amide series **11** (Scheme 2), benzylic bromination of **16** with *N*-bromosuccinimide (NBS) afforded **19** in good yield, analogous to the conditions employed for the chromanone analogue [4]. Nucleophilic substitution with sodium azide proceeded smoothly to azide **20**, which was reduced either under a Staudinger protocol or via zinc powder in aqueous media to afford amine **21**. Amidation of the primary amine nitrogen was achieved using EDC coupling with the appropriate acid under basic conditions.

**Scheme 3:**
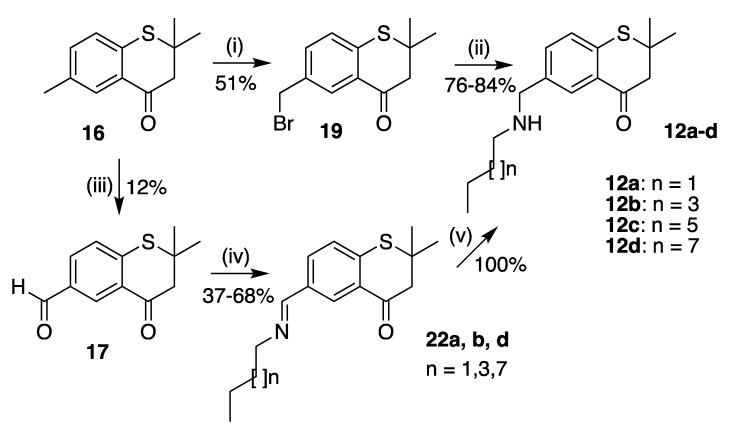
Synthesis of compounds **12a**–**d**. *Reagents and conditions*: (i) NBS, dibenzoyl peroxide, cyclohexane, 95–100 °C, 8 h; (ii) Alkylamine, CH_3_CN, Δ, 6 h; (iii) K_2_S_2_O_8_, CuSO_4_, H_2_O/CH_3_CN (1:1), 75–80 °C, 1 h; (iv) Alkylamine, STAB, CH_3_COOH, THF/DCM, RT, 24 h; (v) NaBH_4_, MeOH, RT, 1 h.

In parallel, amine series **12** was prepared (Scheme 3) via substitution of bromide synthon **19** in refluxing acetonitrile, or by first oxidising **16** to aldehyde **17** before reductive amination with sodium triacetoxyborohydride (STAB). Direct coupling of the bromide with primary amines proceeded almost instantaneously, yet required careful stoichiometric control and subsequent chromatography to avoid residual contaminating amine. On the other hand, particularly for the higher-boiling alkylamines, reductive amination proved more favourable, with the stable intermediate imine **22** being purified prior to regioselective reduction with one equivalent of sodium borohydride.

**Scheme 4:**
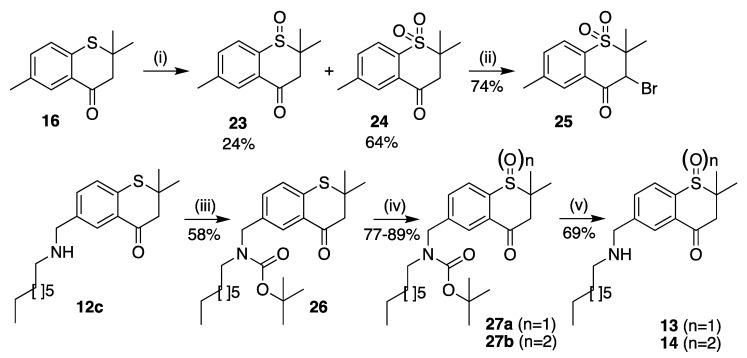
Synthesis of compounds **13** and **14**. *Reagents and conditions*: (i) NaBO_3_.4H_2_O, GAA, 55 °C, 4 h; (ii) NBS, dibenzoyl peroxide, cyclohexane, 95–100 °C, 8 h; (iii) Boc_2_O, DCM, RT, 12 h; (iv) 35% H_2_O_2_, Montmorillonite K10, RT, 12 h or NaBO_3_.4H_2_O, GAA, 55 °C, 4 h; (v) TFA, DCM, 0 °C-RT, 24 h.

To provide sulfur oxides **13** and **14**, successive oxidations of **16** were envisaged. Although perborate-catalyzed oxidation of **16** afforded both sulfoxide **23** and sulfone **24** products (Scheme 4), further reaction with persulfate did not proceed. Under bromination conditions with NBS, in the case of **23,** decomposition occurred, while alpha bromination of **24** occurred with no accompanying benzylic bromination. Protection of the secondary nitrogen in **12c** with a BOC group prior to oxidation with hydrogen peroxide in the presence of Montmorillonite K10 [19] obtained sulfoxide **27a**. Likewise, perborate oxidation of **26** in glacial acetic acid (GAA) [20] afforded **27b**. Deprotection of these BOC-amides afforded the respective sulfoxide **13** and sulfone **14**.

### 2.2. Computational Study

Simple molecular descriptors were calculated using the Molinspiration online property calculation toolkit (http://www.molinspiration.com, accessed on 1 February 2021). Results are shown in Table 1.

### 2.3. Pharmacological Activity

Selected compounds were evaluated for their leishmanicidal ability as previously described [21], through examining their activity on *L. infantum* axenic amastigotes, and the results are expressed in Table 2 as IC_50_ values. In parallel, evaluation of the cytotoxicity of test compounds was performed by MTT assay using the J774A.1 macrophage cell line. Results are presented as CC_50_ values.

## 3. Discussion

Thirteen novel thiochromanone analogues were synthesised in this work, all esters (**10a**,**b**), amides (**11a**–**e**) or amines (**12a**–**d**, **13**, **14**) at the 6-position of a 2,2-dimethylthiochromanome core.

To obey Lipinski’s rule, orally active drugs should have: (a) no more than 5 hydrogen bond donors (n-OHNH); (b) no more than 10 hydrogen acceptors (n-ON); (c) an octanol–water partition coefficient (log P) not >5; and (d) a molecular weight <500 Da. Of our synthesised compounds, seven fulfilled Lipinski’s rule, with six each showing one violation. Rotatable bond count (nRotb), molecular volume (Vol.) and topological polar surface area (TPSA) were also calculated for the compounds. Molecular volume is a function of molecular weight and structure and considers all accessible conformations available to the molecule under physiological conditions, and alongside TPSA, is used to predict drug transport properties. Drugs of poor bioavailability and absorption have high TPSA values. Our compounds had TPSA values ranging from 29 to 65, with compound **12c**, the thio isostere of **3** showing predicted improved bioavailabilty with a lower TPSA score.

Compounds **10a** and **10b** are ester derivatives of previously synthesised chromanone esters, and while both compounds showed activity against *L. infantum*, styryl ester **10a** was three-fold more active, with an IC_50_ of 34.4 μM. As the ester moiety of these molecules is susceptible to hydrolysis, we prepared series **11**, a group of amides. A comparison of the activity of **10a** and **11a** revealed that the amide analogue was over three times more active than the ester against *L. infantum*, perhaps reflecting the expected better stability profile. However, piperamide **11c** was devoid of activity. Within amides **11**, extending the lipophilic chain of **11a** by two carbons, as in **11b**, halved activity (10.5 vs. 19.9 μM). While a linear octanoyl chain in **11d** reduced activity 5-fold compared to the styryl derivative, the haloalkyl unit in **11e** resulted in the most active compound (IC_50_ 7.2 μM), superior to the phenylalkenyl derivative **11a**. Within the alkylamide compounds **12a**–**d**, extension of the lipophilic carbon chain by two atoms each time correlated with an increase in activity, with the decylamine derivative **12d** having an IC_50_ of 12.4 μM. Compound **12c** (IC_50_ 19.5 μM) represents the thio isostere of the previously reported **3**, which inhibited axenic amastigotes of *L. infantum* with an IC_50_ of 25.3 μM, suggesting that the sulfur atom results in a moderate improvement in activity. However, we must note the cytotoxicity of some of the compounds, notably **11b** and alkylamines **12**, which although showing interesting activity against axenic amastigotes, were comparably cytotoxic to macrophages. Structures with similarity to compounds **4** and **5** may have effects as membrane disruptors; this needs further exploration within our series of compounds to try and optimise promising anti-leishmanial activity but without appreciable toxicity. Additional work should involve exploration of likely mechanisms of action, such as inhibition of pteridine reductase 1 (PTR1), a proposed mechanism of action of analogous chroman-4-one derivatives [22,23]. In conclusion, compounds **11a** and **11e** represent interesting compounds, with notable anti-leishmanial activity and good selectivity.

## 4. Materials and Methods

### 4.1. Chemistry

All required chemicals, solvents, and reagents were purchased from Sigma-Aldrich (Arklow, Ireland) and were of reagent grade. Reaction progress was monitored on pre-coated thin layer chromatographic aluminum sheets (silica gel 60 F254, Merck, Carrigtwohill, Ireland), and TLC visualization was done using a UV lamp. Fourier transform infrared spectra were carried out with neat film coated samples on diamond using a Nicolet^TM^ iS^TM^ 10 FT-IR spectrophotometer (Thermo Fisher, Dublin, Ireland). Significant absorption peak (νmax) values are given in cm^−1^. ^1^H- and ^13^C-NMR spectra were recorded on an Avance 400 spectrometer (Bruker, Rheinstetten, Germany) at 400 MHz and 100 MHz, respectively, in CDCl_3_ and CD_3_OD, using tetramethylsilane (TMS) as the internal standard (Spectra available in Supplementary Materials). Chemical shift values are given on the δ (ppm) scale, with signals described as follows: s (singlet), d (doublet), dd (double doublet), t (triplet), q (quartet), br. (broad signal), m (multiplet), and coupling constants (*J*) expressed in Hz. Mass spectral analyses were recorded using a LCT Premiere XE (ESI-TOF MS) instrument (Waters, Dublin, Ireland). All calculated exact mono isotopic mass distributions were calibrated against internal reference standards.

#### *2,2,6-Trimethylthiochroman-4-one* (**16**)

This compound was prepared and characterized as previously described [17]. Yield: 33%.

#### *2,2-Dimethyl-4-oxothiochromane-6-carbaldehyde* (**17**)

To a solution of **16** (0.53 g, 2.57 mmol) in acetonitrile (15 mL) was added a solution of potassium persulfate (1.39 g, 5.14 mmol) in water (15 mL), and copper sulfate (0.13 g, 0.52 mmol). The resulting solution was stirred at 75–80 °C for 1 h and then cooled. The cold mixture was diluted with saturated sodium bicarbonate solution and extracted with diethyl ether (3 × 50 mL). The diethyl ether extracts were concentrated in vacuo and the residue purified by flash chromatography (pet. ether/EtOAc 10:1) to give **17** as a pale oil that solidified on standing (70 mg, 12%). ^1^H-NMR (CDCl_3_) δ_H_ 1.43 (6H, s, C(CH_3_)_2_), 2.86 (2H, s, CH_2_), 7.30 (1H, d, *J* = 8.2 Hz, ArH), 7.84 (1H, d, *J* = 7.9 Hz, ArH), 8.47 (1H, s, H5), 9.90 (1H, s, CHO). ^13^C-NMR δ_C_ 28.6 ((CH_3_)_2_), 45.3 (C(CH_3_)_2_), 53.2 (CH_2_), 128.4 (ArCH), 129.6 (ArC), 131.6 (ArCH), 132.1 (ArCH), 133.1 (ArC), 149.5 (ArC), 190.7 (CHO), 193.8 (C=O).

#### *6-(Hydroxymethyl)-2,2-dimethylthiochroman-4-one* (**18**)

To **17** (70 mg, 0.032 mmol) in DMF (1 mL) was added distilled water (50 mL) and freshly cut slices of *D. carota* (10 g). The resulting mixture was stirred vigorously at room temperature for 72 h. The reaction was filtered, and the filtrate washed with ethyl acetate (50 mL). The water/ethyl acetate mixture was separated, and the ethyl acetate extract dried over Na_2_SO_4_. The crude orange oil was purified by flash column chromatography (pet. ether/EtOAc 7:1) to afford alcohol **18**, (54 mg, 76%). ^1^H-NMR (CDCl_3_) δ_H_ 1.47 (6H, s, CH_3_), 2.88 (2H, s, CH_2_), 4.69 (2H, s, CH_2_OH), 5.30 (1H, s, OH), 7.24 (1H, d, ArH), 7.46 (1H, dd, *J* = 8.3, 1.7 Hz, ArH), 8.08 (1H, s, H5).

#### *(2,2-Dimethyl-4-oxothiochroman-6-yl)methyl (E)-4-phenylbut-3-enoate* (**10a**)

To a solution of *trans*-styrylacetic acid (15 mg, 0.09 mmol) in dichloromethane (5 mL) was added EDC-HCl (30 mg, 0.16 mmol) and 4-(dimethylamino)pyridine (DMAP) (2 mg). To this solution was added **18** (20 mg, 0.09 mmol). The reaction was stirred for 72 h at room temperature. The residual solvent was removed in vacuo, and the crude residue purified by flash column chromatography (pet. ether/EtOAc 10:1) to afford **10a** as a pale, straw-coloured oil (25 mg, 76%). IR (neat) ν_max_ 964, 1147, 1190, 1678, 1733, 2924 cm^−1^. ^1^H- NMR (CDCl_3_) δ_H_ 1.47 (6H, s, (CH_3_)_2_), 2.88 (2H, s, CH_2_CO), 3.30 (2H, d, *J* = 7.1 Hz, CH_2_COO), 5.12 (2H, s, OCH_2_), 6.31 (1H, m, CH=C), 6.50 (1H, m, CH=C), 7.21–7.43 (7H, m, 7 × ArH), 8.11 (1H, d, *J* = 1.5 Hz, H5). ^13^C NMR δ_C_ 28.6 (2C), 38.3, 44.8, 53.7, 65.8, 121.4, 126.3 (2C), 127.6, 128.0, 128.5, 128.6 (2C), 129.7, 132.5, 133.6, 133.7, 136.8, 141.7, 171.4, 194.7. HRMS (M + NH_4_)^+^ 384.1611, C_22_H_26_NO_3_S requires 384.1628.

#### *(2,2-Dimethyl-4-oxothiochroman-6-yl)methyl (2E,4E)-5-(benzo[d][1,3]dioxol-5-yl)penta-2,4-dienoate* (**10b**)

Compound **18** (25 mg, 0.11 mmol) was reacted as for **10a** with piperic acid (24 mg, 0.11 mmol) to afford **10b** as a straw-coloured oil (31 mg, 65%). IR (neat) ν_max_ 994, 1035, 1126, 1232, 1250, 1445, 1603, 1677, 1705, 2924 cm^−1^. ^1^H-NMR (CDCl_3_) δ_H_ 1.47 (6H, s, (CH_3_)_2_), 2.88 (2H, s, CH_2_CO), 5.17 (2H, s, ArCH_2_O), 5.98 (3H, m, OCH_2_O & CH=C), 6.70 (1H, m, C=CH), 6.78–6.64 (2H, m, 2 × C=CH), 6.91 (1H, d, *J* = 8.3 Hz, C=CH), 6.99 (1H, s, C=CH), 7.24 (1H, d, *J* = 8.1 Hz, C=CH), 7.42–7.48 (2H, m, 2 × C=CH), 8.13 (1H, br. s, H5). ^13^C-NMR δ_C_ 28.6 (2C), 44.8, 53.8, 65.4, 101.4, 105.9, 108.6, 119.7, 123.1, 124.4, 127.9, 128.5, 129.7, 130.5, 133.0, 133.6, 140.7, 141.5, 145.6, 148.3, 148.7, 166.9, 194.7. HRMS (M + NH_4_)^+^ 440.1570, C_24_H_26_NO_5_S requires 440.1526.

#### *6-(Bromomethyl)-2,2-dimethylthiochroman-4-one* (**19**)

Compound **16** (1.55 g (7.5 mmol) was dissolved in cyclohexane (25 mL). To this stirred solution was added *N*-bromosuccinimide (2.66 g, 14.9 mmol) and a catalytic quantity of dibenzoylperoxide, and the reaction mixture was stirred at 100 °C for 6 h. Upon completion, the solvent was removed *in vacuo*, and the residue purified by flash chromatography (pet. ether/EtOAc 10:1) to give **19** as a golden oil (1.072 g, 51%), which solidified on standing. IR ν_max_ (neat) 1676 cm^−1^; ^1^H-NMR (CDCl_3_) δ_H_ 1.47 (6H, s, (CH_3_)_2_, 2.87 (2H, s, CH_2_), 4.48 (2H, s, ArCH_2_), 7.22 (1H, d, *J* = 8.2 Hz, H8), 7.45 (1H, dd, *J* = 8.2, 2.1 Hz, H7), 8.10 (1H, d, *J* = 2.2 Hz, H5); ^13^C-NMR δ_C_ 28.6 ((CH_3_)_2_), 32.7 (CH_2_), 44.9(C(CH_3_)_2_), 53.6 (CH_2_), 128.3 (ArCH), 128.9 (ArCH), 129.7 (ArC), 134.2 (ArCH), 134.4 (ArC), 141.9 (ArC), 194.5 (C=O).

#### *6-(Azidomethyl)-2,2-dimethylthiochroman-4-one* (**20**)

To a solution of **19** (0.81 g, 2.84 mmol) in DMF (6 mL) was added sodium azide (2 g, 30.8 mmol). The resultant slurry was heated on an oil bath for 4 h at 50 °C, and then partitioned between water and ethyl acetate. The aqueous layer was washed twice with ethyl acetate (2 × 20 mL portions) and the combined organic layers dried over sodium sulfate and concentrated *in vacuo*. The residue was purified by flash column chromatography on silica gel (pet. ether/EtOAc 10:1) to yield the azide (0.52 g, 74%) as a bright yellow oil. ^1^H- NMR (CDCl_3_) δ_H_ 1.48 (6H, s, (CH_3_)_2_, 2.89 (2H, s, CH_2_CO), 4.34 (2H, s, CH_2_N_3_), 7.27 (1H, d, *J* = 8 Hz, H8), 7.38 (1H, dd, *J* = 8, 2 Hz, H7), 8.04 (1H, d, *J* = 1.7 Hz, H5); ^13^C-NMR δ_C_ 28.6 ((CH_3_)_2_), 44.8 (C2), 53.7 (CH_2_), 54.1 (CH_2_), 128.3 (ArCH), 128.3 (ArCH), 129.7 (ArC), 132.1 (ArC), 133.3 (ArCH), 141.7 (ArC), 194.6 (C=O).

#### *6-(Aminomethyl)-2,2-dimethylthiochroman-4-one* (**21**)

To **20** (57 mg, 0.23 mmol) in EtOH/H_2_O (3:1, 5 mL) was added Zn (20 mg, 0.31 mmol) and NH_4_Cl (25 mg, 0.47 mmol). The mixture was stirred vigorously at room temperature for 24 h followed by reflux for 6 h. Ethyl acetate (20 mL) and NaOH (1 M) (1 mL) were added. The mixture was filtered, and the filtrate was washed with brine, dried over anhydrous sodium sulfate. Removal of solvent under reduced pressure afforded a yellow oil. ^1^H-NMR (CDCl_3_) δ_H_ 1.47 (6H, s, (CH_3_)_2_, 2.88 (2H, s, CH_2_CO), 3.86 (2H, s, CH_2_NH_2_), 7.21 (1H, d, *J* = 8.1 Hz, H8), 7.40 (1H, dd, *J* = 8.1, 1.8 Hz, H7), 8.04 (1H, d, *J* = 1.3 Hz, H5); ^13^C- NMR δ_C_ 28.6 ((CH_3_)_2_), 44.7 (C2), 45.7 (CH_2_), 53.9 (CH_2_), 127.0 (ArCH), 127.9 (ArCH), 129.6 (ArC), 132.9 (ArCH), 133.5 (ArC), 139.7 (ArC), 195.1 (C=O). As an alternative method, to a mixture of **20** (462 mg, 1.87 mmol) in THF (10 mL) and H_2_O (1 mL) was added PPh_3_ (0.86 g, 3.29 mmol). The reaction mixture was stirred at RT for 12 h. The mixture was acidified to pH = 1 with 1N HCl and extracted with EtOAc (100 mL). The aqueous layer was separated and the pH adjusted to pH 10 with 1N NaOH, and extracted with DCM (3 × 100 mL). The residual solvent was removed *in vacuo* to afford 302 mg (73%) of **21**.

#### *(E)-N-((2,2-Dimethyl-4-oxothiochroman-6-yl)methyl)-4-phenylbut-3-enamide* (**11a**)

To a solution of *trans*-styrylacetic acid (41 mg, 0.25 mmol) in dichloromethane (5 mL) at 0 °C under a N_2_ atmosphere was added EDC-HCl (100 mg, 0.52 mmol) and triethylamine (0.09 mL, 0.65 mmol). After 30 min, **21** (50 mg, 0.23 mmol) was added to this solution. The reaction was allowed to reach room temperature and stirred for 24 h. The residual solvent was removed in vacuo, the residue extracted with saturated NaHCO_3_/DCM, and the crude organic residue purified by flash column chromatography (pet. ether/EtOAc 5:1) to afford amide **11a** as a pale oil (56 mg, 67%). IR (neat) ν_max_ 965, 1186, 1212, 1304, 1468, 1538, 1675, 2923 cm^−1^; ^1^H-NMR (CDCl_3_) δ_H_ 1.46 (6H, s, (CH_3_)_2_, 2.86 (2H, s, CH_2_CO), 3.22 (2H, d, *J* = 7.1 Hz, CH_2_C=C), 4.43 (2H, d, *J* = 5.9 Hz, CH_2_N), 5.97 (1H, br., NH), 6.31 (1H, m, CH_2_CH=CH), 6.55 (1H, m, CH_2_CH=CH), 7.19–7.39 (7H, m, 7 × ArH), 7.98 (1H, d, *J* = 1.7 Hz, H5); ^13^C-NMR δ_C_ 28.6 ((CH_3_)_2_), 40.8 (CH_2_), 43.0 (CH_2_), 44.7 (C2), 53.8 (CH_2_), 122.0, 126.4 (2C), 127.6, 127.9, 128.2, 128.7 (2C), 133.4, 134.9 (ArC), 135.1, 140.7 (ArC), 170.7 (NHC=O), 194.8 (C=O) (*2C* signals obscured). HRMS (M + Na)^+^ 388.1361, C_22_H_23_NO_2_NaS requires 388.1347.

#### *(2,2-Dimethyl-4-oxothiochroman-6-yl)methyl-6-phenylhex-5-enoate (E:Z 3:1)* (**11b**)

Compound **21** (87 mg (0.39 mmol) was reacted as described for **11a** with 6-phenylhex-5-enoic acid (75 mg, 0.39 mmol) to afford **11b** as a pale straw-coloured oil that on standing became a semi-solid gum (113 mg, 73%). IR (neat) ν_max_ 964, 1091, 1213, 1304, 1466, 1648, 1676, 2852, 2922, 2955 cm^−1^; ^1^H-NMR (CDCl_3_) δ_H_ 1.46 (6H, s, (CH_3_)_2_, 1.82–1.90 (2H, m, CH_2_), 2.19–2.40 (4H, m, NHCOCH_2_ & C=CHCH_2_), 2.86 (2H, s, CH_2_CO), 4.30 & 4.41 (2H, 2 × d, *J* = 5.7 Hz, ArCH_2_N), 5.53 & 5.81 (1H, br., NH), 6.18 (1H, m, CH=CHAr), 6.37 & 6.46 (1H, 2 × d, *J* = 15.9 & 11.7 Hz, CH=CHAr), 7.15–7.36 (7H, m, 7 × ArH), 7.93 & 7.97 (1H, 2 × s, H5); ^13^C-NMR δ_C_ 25.1 (CH_2_), 28.6 ((CH_3_)_2_), 32.4 (CH_2_), 35.9 (CH_2_), 42.9 (CH_2_), 44.7 (C2), 53.8 (CH_2_), 126.0 (2C), 127.0, 127.5, 128.1, 128.2, 128.5 (2C), 129.7, 130.8, 133.4, 135.2 (ArC), 137.5 (ArC), 140.6 (ArC), 172.7 (NHC=O), 194.8 (C=O), (*signals for E isomer reported, 1 quaternary signal obscured*); HRMS (M + Na)^+^ 416.1645, C_24_H_27_NO_2_NaS requires 416.1660.

#### *(2E,4E)-5-(Benzo[d][1,3]dioxol-5-yl)-N-((2,2-dimethyl-4-oxothiochroman-6-yl)methyl)penta-2,4-dienamide* (**11c**)

Compound **21** (80 mg, 0.36 mmol) was reacted as for **11a** with piperic acid (79 mg, 0.36 mmol) to afford **11c** as a pale yellow solid (63 mg, 41%). IR (neat) ν_max_ 982, 1036, 1190, 1250, 1443, 1536, 1614, 1644, 1679, 3278 cm^−1^; ^1^H-NMR (CDCl_3_-) δ_H_ 1.45 (6H, s, (CH_3_)_2_, 2.85 (2H, s, CH_2_), 4.51 (2H, d, *J* = 5.9 Hz, CH_2_N), 5.94 (1H, d, *J* = 14.9 Hz, COCH=C), 5.98 (2H, s, OCH_2_O), 6.04 (1H, br. t, NH), 6.65 & 6.68 (1H, 2 × d, *J* = 10.6 Hz, CH=CH), 6.78 (2H, m, 2 × CH=C), 6.89 (1H, d, *J* = 8.1 Hz, CH=C), 6.97 (1H, br. s, CH=C), 7.20 (1H, d, *J* = 8.1 Hz, CH=C), 7.39 (2H, m, 2 × CH=C), 8.00 (1H, br. s, CH=C); ^13^C-NMR δ_C_ 28.6 ((CH_3_)_2_), 43.0 (CH_2_), 44.7 (C(CH_3_)_2_), 53.8 (CH_2_), 101.3 (OCH_2_O), 105.8 (CH=C), 108.5 (CH=C), 122.5 (CH=C), 122.8 (CH=C), 124.5 (CH=C), 127.5 (CH=C), 128.1 (CH=C), 129.6 (ArC), 130.8 (ArC), 133.5 (CH=C), 135.2 (ArC), 139.4 (CH=C), 140.6 (ArC), 141.8 (ArCH), 148.2 (ArC), 148.3 (ArC), 166.2 (NC=O), 195.0 (C=O); HRMS (M + H)^+^ 422.1432, C_24_H_24_NO_4_S requires 422.1426.

#### *N-((2,2-Dimethyl-4-oxothiochroman-6-yl)methyl)octanamide* (**11d**)

Compound **21** (87 mg, 0.39 mmol) was reacted as for **11a** with octanoic acid (56 mg, 0.39 mmol) to afford **11d** as a pale straw-coloured oil that on standing became a semi-solid gum (102 mg, 75%). IR (neat) ν_max_ 1193, 1306, 1407, 1462, 1530, 1639, 1680, 2852, 2923, 3297 cm^−1^; ^1^H-NMR (CDCl_3_, 400 MHz) δ_H_ 0.87 (3H, t, CH_2_CH_3_), 1.28 (m, 8H, 4 × CH_2_), 1.46 (6H, (CH_3_)_2_), 1.65 (2H, m, CH_2_), 2.22 (2H, m, CH_2_CONH), 2.86 (2H, s, CH_2_CO), 4.41 (2H, d, *J* = 5.9 Hz, CH_2_N), 5.86 (1H, br., NH), 7.20 (1H, d, *J* = 8.1 Hz, H8), 7.36 (1H, dd, *J* = 8.1, 2.0 Hz, H7), 7.97 (1H, *J* = 1.5 Hz, H5); ^13^C-NMR δ_C_ 14.1 (CH_3_), 22.6 (CH_2_), 25.7 (CH_2_), 28.6 ((CH_3_)_2_), 29.0 (CH_2_), 29.3 (CH_2_), 31.7 (CH_2_), 36.8 (CH_2_), 42.8 (CH_2_), 44.7 (C(CH_3_)_2_), 53.8 (CH_2_), 127.4 (ArCH), 128.1 (ArCH), 129.6 (ArC), 133.4 (ArCH), 135.3 (ArC), 140.6 (ArC), 173.2 (NC=O), 194.9 (C=O); HRMS (M + Na)^+^ 370.1831, C_20_H_29_NO_2_NaS requires 370.1817.

#### *9-Bromo-N-((2,2-dimethyl-4-oxothiochroman-6-yl)methyl)nonanamide* (**11e**)

Compound **21** (100 mg, 0.45 mmol) was reacted as for **11a** with 9-bromononanoic acid (100 mg, 0.42 mmol) to afford **11e** as a pale straw-coloured oil that on standing became a semi-solid gum (123 mg, 62%). IR (neat) ν_max_ 1215, 1300, 1463, 1539, 1647, 1675, 2855, 2924 cm^−1^; ^1^H-NMR (CDCl_3_) δ_H_ 1.25–1.86 (m, 12H, 6 × CH_2_), 1.46 (6H, s, (CH_3_)_2_, 2.22 (2H, m, NHCOCH_2_), 2.86 (2H, s, CH_2_CO), 3.41 (2H, m, CH_2_Br), 4.42 (2H, d, *J* = 5.9 Hz, CH_2_N), 5.82 (1H, br., NH), 7.21 (1H, d, *J* = 8.2 Hz, H8), 7.36 (1H, dd, *J* = 8.2, 2.2 Hz, H7), 7.97 (1H, dd, *J* = 2, 0.2 Hz, H5); ^13^C-NMR δ_C_ 25.6 (CH_2_), 28.1 (CH_2_), 28.5 (CH_2_), 28.6 ((CH_3_)_2_), 29.1 (CH_2_), 29.2 (CH_2_), 32.8 (CH_2_), 34.0 (CH_2_), 36.7 (CH_2_), 42.8 (CH_2_), 44.7 (C(CH_3_)_2_), 53.8 (CH_2_), 127.4 (ArCH), 128.1 (ArCH), 129.7 (ArC), 133.4 (ArCH), 135.3 (ArC), 140.6 (ArC), 173.0 (NC=O), 194.9 (C=O); HRMS (M + Na)^+^ 462.1078, C_21_H_30_NO_2_NaSBr requires 462.1078.

#### *6-((Butylamino)methyl)-2,2-dimethylthiochroman-4-one* (**12a**)

Compound **19** (100 mg, 0.35 mmol) was dissolved in acetonitrile (25 mL) under aerobic conditions. To this stirred solution was added excess *n*-butylamine, and the reaction mixture was stirred at 100 °C for 2 h. Upon completion, the solvent was removed *in vacuo*, and the residue purified by flash chromatography (pet. ether/EtOAc 2:1) to give **12a** as a pale brown oil (74 mg, 76%), without contamination with any tertiary amine. IR ν_max_ (neat) 1249, 1464, 1601, 1679 cm^−1^; ^1^H-NMR (CDCl_3_) δ_H_ 0.91 (3H, t, *J* = 7.3 Hz, CH_3_), 1.34 (2H, m, (CH_2_), 1.46 (6H, s, (CH_3_)_2_, 1.48 (2H, m, CH_2_), 2.61 (2H, t, *J* = 7.2 Hz, (NCH_2_CH_2_), 2.87 (2H, s, CH_2_), 3.76 (2H, s, ArCH_2_), 7.20 (1H, d, *J* = 8.1 Hz, H8), 7.42 (1H, dd, *J* = 8.1, 1.6 Hz, H7), 8.03 (1H, br. d, H5); ^13^C-NMR δ_C_ 14.0 (CH_3_), 20.5 (CH_2_), 28.6 ((CH_3_)_2_), 32.2 (CH_2_), 44.6 (C(CH_3_)_2_), 49.1 (CH_2_), 53.3 (CH_2_), 53.9 (CH_2_), 127.8 (ArCH), 128.1 (ArCH), 129.5 (ArC), 133.8 (ArCH), 137.3 (ArC), 139.8 (ArC), 195.1 (C=O); HRMS (M + H)^+^ 278.1565, C_16_H_24_NOS requires 278.1579.

#### *6-((Hexylamino)methyl)-2,2-dimethylthiochroman-4-one* (**12b**)

Compound **19** (100 mg, 0.35 mmol) was reacted as for **12a** with *n*-hexylamine to afford **12b** as a pale brown oil (82 mg, 77%). IR ν_max_ (neat) 1251, 1454, 1528, 1683, 3336 cm^−1^; ^1^H- NMR (CDCl_3_) δ_H_ 0.88 (3H, t, *J* = 6.7 Hz, CH_3_), 1.28 (6H, m, (CH_2_)_3_), 1.46 (6H, s, (CH_3_)_2_, 1.48 (2H, m, CH_2_), 2.60 (2H, t, *J* = 7.3 Hz, (NCH_2_CH_2_), 2.87 (2H, s, CH_2_), 3.76 (2H, s, ArCH_2_), 7.20 (1H, d, *J* = 8.2 Hz, H8), 7.42 (1H, dd, *J* = 8.1, 2.1 Hz, H7), 8.02 (1H, d, *J* = 1.3 Hz, H5); ^13^C-NMR δ_C_ 14.1 (CH_3_), 22.6 (CH_2_), 27.0 (CH_2_), 28.6 ((CH_3_)_2_), 30.0 (CH_2_), 31.8 (CH_2_), 44.6 (C(CH_3_)_2_), 49.5 (CH_2_), 53.3 (CH_2_), 53.9 (CH_2_), 127.7 (ArCH), 128.1 (ArCH), 129.5 (ArC), 133.8 (ArCH), 137.3 (ArC), 139.8 (ArC), 195.1 (C=O); HRMS (M+H)^+^ 306.1899, C_18_H_28_NOS requires 306.1892.

#### *2,2-Dimethyl-6-((octylamino)methyl)thiochroman-4-one* (**12c**) 

Compound **19** (100 mg, 0.35 mmol) was reacted as for **12a** with *n*-octylamine (45 mg, 0.35 mmol) to afford **12c** as a pale brown oil (98 mg, 84%). IR ν_max_ (neat) 1303, 1465, 1600, 1678, 2922 cm^−1^; ^1^H-NMR (CDCl_3_) δ_H_ 0.87 (3H, t, *J* = 6.8 Hz, CH_3_), 1.22–1.32 (10H, m, (CH_2_)_5_), 1.46 (6H, s, (CH_3_)_2_, 1.48 (2H, m, CH_2_), 2.60 (2H, t, *J* = 7.2 Hz, (NCH_2_CH_2_), 2.87 (2H, s, CH_2_), 3.76 (2H, s, ArCH_2_), 7.20 (1H, d, *J* = 8.2 Hz, H8), 7.42 (1H, dd, *J* = 8.1, 1.8 Hz, H7), 8.02 (1H, d, *J* = 1.5 Hz, H5); ^13^C-NMR δ_C_ 14.1 (CH_3_), 22.7 (CH_2_), 27.4 (CH_2_), 28.6 ((CH_3_)_2_), 29.3 (CH_2_), 29.5 (CH_2_), 30.1 (CH_2_), 31.8 (CH_2_), 44.6 (C(CH_3_)_2_), 49.5 (CH_2_), 53.3 (CH_2_), 53.9 (CH_2_), 127.8 (ArCH), 128.1 (ArCH), 129.5 (ArC), 133.8 (ArCH), 137.3 (ArC), 139.8 (ArC), 195.1 (C=O); HRMS (M+H)^+^ 334.2195, C_20_H_32_NOS requires 334.2205.

#### *2,2-Dimethyl-6-((decylamino)methyl)thiochroman-4-one* (**12d**)

Compound **19** (100 mg, 0.35 mmol) was reacted as for **12a** with *n*-decylamine (55 mg, 0.35 mmol) to afford **12d** as a pale golden oil (100 mg, 79%). IR ν_max_ (neat) 1189, 1301, 1387, 1468, 1510, 1675, 2852, 2922 cm^−1^; ^1^H-NMR (CDCl_3_-) δ_H_ 0.88 (3H, t, *J* = 6.8 Hz, CH_3_), 1.20–1.33 (14H, m, (CH_2_)_7_), 1.47 (6H, s, (CH_3_)_2_, 1.49 (2H, m, CH_2_), 2.61 (2H, t, *J* = 7.2 Hz, (NCH_2_CH_2_), 2.87 (2H, s, CH_2_), 3.77 (2H, s, ArCH_2_), 7.20 (1H, d, *J* = 8.1 Hz, H8), 7.43 (1H, dd, *J* = 8.2, 1.6 Hz, H7), 8.02 (1H, d, *J* = 1.2 Hz, H5); ^13^C-NMR δ_C_ 14.2 (CH_3_), 22.7 (CH_2_), 27.3 (CH_2_), 28.6 ((CH_3_)_2_), 29.3 (CH_2_), 29.57 (CH_2_), 29.59 (CH_2_), 29.62 (CH_2_), 30.0 (CH_2_), 31.9 (CH_2_), 44.7 (C(CH_3_)_2_), 49.4 (CH_2_), 53.2 (CH_2_), 53.9 (CH_2_), 127.8 (ArCH), 128.2 (ArCH), 129.5 (ArC), 133.9 (ArCH), 137.1 (ArC), 139.9 (ArC), 195.1 (C=O); HRMS (M+H)^+^ 362.2519, C_22_H_36_NOS requires 362.2518.

#### *6-((Butylimino)methyl)-2,2-dimethylthiochroman-4-one* (**22a**)

To a solution of **17** (100 mg, 0.45 mmol) in tetrahydrofuran (10 mL) was added *n*-butylamine (33 mg, 0.45 mmol) and sodium triacetoxyborohydride (144 mg, 0.68 mmol). To the stirred suspension was added acetic acid (20 μL, 0.35 mmol). The reaction was stirred under a N_2_ atmosphere at room temperature overnight. The reaction mixture was quenched by adding aqueous saturated NaHCO_3_, and the product was extracted with EtOAc (3 × 30 mL). The EtOAc extract was dried (Na_2_SO_4_), and the solvent was evaporated to give the crude imine as a clear oil (84 mg, 67%). ^1^H-NMR (CDCl_3_-) δ_H_ 0.86 (3H, t, *J* = 7.4 Hz, CH_2_CH_3_), 1.29 (2H, m, CH_2_), 1.40 (6H, s, C(CH_3_)_2_), 1.58 (2H, m, CH_2_), 2.81 (2H, s, CH_2_), 3.53 (2H, t, *J* = 7 Hz, NCH_2_), 7.19 (1H, d, *J* = 8.3 Hz, H8), 7.84 (1H, dd, *J* = 8.3, 1.4 Hz, H7), 8.18 (2H, m, H5 & CH=N). ^13^C-NMR δ_C_ 13.9 (CH_3_), 20.4 (CH_2_), 28.6 ((CH_3_)_2_), 32.9 (CH_2_), 44.9 (C(CH_3_)_2_), 53.6 (CH_2_), 61.3 (CH_2_), 128.0 (ArCH), 129.3 (ArCH), 129.5 (ArC), 131.4 (ArCH), 133.2 (ArC), 144.1 (ArC), 159.4 (N=CH), 194.5 (C=O).

#### *6-((Hexylimino)methyl)-2,2-dimethylthiochroman-4-one* (**22b**)

Compound **17** (98 mg, 0.44 mmol) was reacted as described for **22a** with *n*-hexylamine (45 mg, 0.44 mmol) to afford **22b** as a clear oil (92 mg, 68%). ^1^H-NMR (CDCl_3_) δ_H_ 0.81 (3H, t, CH_2_CH_3_), 1.23–1.29 (6H, m, (CH_2_)_3_), 1.41 (6H, s, C(CH_3_)_2_), 1.61 (2H, m, CH_2_), 2.82 (2H, s, CH_2_), 3.52 (2H, t, *J* = 7 Hz, NCH_2_), 7.20 (1H, d, *J* = 8.3 Hz, H8), 7.85 (1H, dd, *J* = 8.3, 2.1 Hz, H7), 8.20 (2H, m, H5 & CH=N). ^13^C-NMR δ_C_ 13.1 (CH_3_), 21.6 (CH_2_), 26.0 (CH_2_), 27.6 ((CH_3_)_2_), 29.8 (CH_2_), 30.6 (CH_2_), 43.9 (C(CH_3_)_2_), 52.6 (CH_2_), 60.7 (CH_2_), 126.8 (ArCH), 128.3 (ArCH), 128.5 (ArC), 130.3 (ArCH), 132.2 (ArC), 143.1 (ArC), 158.3 (N=CH), 193.4 (C=O).

#### *6-((Decylimino)methyl)-2,2-dimethylthiochroman-4-one* (**22d**)

Compound **17** (87 mg, 0.39 mmol) was reacted as for **22a** with *n*-decylamine (62 mg, 0.39 mmol) to afford **22d** as a yellow oil (52 mg, 37%). ^1^H-NMR (CDCl_3_) δ_H_ 0.78 (3H, t, CH_2_CH_3_), 1.20 (10H, m, 5 × CH_2_), 1.38 (6H, s, C(CH_3_)_2_), 1.59 (2H, m, CH_2_), 2.79 (2H, s, CH_2_), 3.50 (2H, t, *J* = 7 Hz, NCH_2_), 7.16 (1H, d, *J* = 8.3, H8), 7.82 (1H, d, *J* = 8.3 Hz, H7), 8.16 (2H, m, H5 & CH=N). ^13^C-NMR δ_C_ 14.1 (CH_3_), 22.7, 25.6, 27.3, 28.5 ((CH_3_)_2_), 29.3, 29.4, 29.6, 30.9, 31.9, 44.9 (C(CH_3_)_2_), 53.6 (CH_2_), 61.7 (CH_2_), 127.9 (ArCH), 129.3 (ArCH), 129.5 (ArC), 131.4 (ArCH), 133.2 (ArC), 144.0 (ArC), 159.2 (N=CH), 194.3 (C=O). 

Reduction of **22a**–**d** with one equivalent of sodium borohydride in methanol for one hour at 0 °C to room temperature afforded **12a**–**d** in quantitative yield.

#### *2,2,6-Trimethylthiochroman-4-one 1-oxide (**23**) and 2,2,6-trimethylthiochroman-4-one 1,1-dioxide* (**24**)

To a suspension of **16** (100 mg, 0.48 mmol) in glacial acetic acid (10 mL) was added sodium perborate·4H_2_O (75 mg, 0.49 mmol), in portions. After stirring at 55 °C for 4 h, the reaction was poured into ice/water (100 mL). Ethyl acetate was added and the aqueous layer extracted (3 × 50 mL). The organic layers were combined, the solvent removed *in vacuo* and the residue purified by flash chromatography (pet. ether/EtOAc 8:1) to give two products. 1. Sulfone **24**, a clear oil (69 mg, 64%); ^1^H-NMR (CDCl_3_) δ_H_ 1.53 (6H, s, (CH_3_)_2_, 2.49 (3H, s, (CH_3_), 3.24 (2H, s, CH_2_), 7.64 (1H, d, *J* = 8.1 Hz, ArH), 7.91 (1H, s, H5), 7.96 (1H, d, *J* = 8.1 Hz, ArH); ^13^C-NMR δ_C_ 21.1 ((CH_3_)_2_), 21.6 (CH_3_), 50.6 (CH_2_), 58.6 (C(CH_3_)_2_), 125.0 (ArCH), 128.4 (ArCH), 130.4 (ArC), 135.8 (ArCH), 136.2 (ArC), 144.2 (ArC), 191.0 (C=O). 2. Sulfoxide **23**, straw coloured oil (28 mg, 24%); ^1^H-NMR (CDCl_3_) δ_H_ 1.35 & 1.41 (6H, 2 × s, (CH_3_)_2_, 2.47 (3H, s, (CH_3_), 2.73 (1H, d, *J* = 17.5 Hz, 1H of CH_2_), 3.24 (1H, d, *J* = 17.5 Hz, 1H of CH_2_), 7.60 (1H, d, *J* = 7.9 Hz, ArH), 7.78 (1H, d, *J* = 8.1 Hz, ArH), 7.95 (1H, s, H5); ^13^C- NMR δ_C_ 20.0 (CH_3_), 21.4 (CH_3_), 24.0 (CH_3_), 45.4 (CH_2_), 56.2 (C(CH_3_)_2_), 128.7 (ArCH), 129.0 (ArC), 129.6 (ArCH), 135.6 (ArCH), 140.6 (ArC), 142.4 (ArC), 192.7 (C=O).

#### *3-Bromo-2,2,6-trimethylthiochroman-4-one 1,1-dioxide* (**25**)

Compound **24** (100 mg, 0.45 mmol) was dissolved in cyclohexane/CH_3_CN (1:1) (25 mL). To this stirred solution was added *N*-bromosuccinimide (120 mg, 0.67 mmol) and a catalytic quantity of dibenzoylperoxide, and the reaction mixture was stirred at 100 °C for 6 h. Upon completion, the solvent was removed *in vacuo*, and the residue purified by flash chromatography (pet. ether/EtOAc 10:1) to give an orange oil (100 mg, 74%). ^1^H-NMR (CDCl_3_) δ_H_ 1.44 & 1.75 (6H, 2 × s, (CH_3_)_2_, 2.52 (3H, s, (CH_3_), 5.82 (1H, s, CH), 7.70 (1H, d, *J* = 8.1 Hz, ArH), 7.95 (1H, d, *J* = 7.9 Hz, ArH), 8.00 (1H, s, H5); ^13^C-NMR δ_C_ 18.8 (CH_3_), 20.2 (CH_3_), 21.7 (CH_3_), 62.0 (CH), 64.8 (C(CH_3_)_2_), 125.0 (ArCH), 127.7 (ArC), 129.8 (ArCH), 135.1 (ArC), 136.4 (ArCH), 144.9 (ArC), 184.8 (C=O).

#### *tert-Butyl ((2,2-dimethyl-4-oxothiochroman-6-yl)methyl)(octyl)carbamate* (**26**)

To a solution of **12c** (200 mg, 0.60 mmol) in DCM (10 mL) at 0 °C was added di-*tert*-butyl dicarbonate (130 mg, 0.60 mmol) and triethylamine (0.08 mL, 0.60 mmol). The reaction was stirred overnight, allowing it to reach room temperature. The solvent was evaporated and the residue purified by flash chromatography (pet. ether/EtOAc 8:1) to give **26** as a yellow oil (150 mg, 58%). ^1^H-NMR (CDCl_3_, 400 MHz) δ_H_ 0.87 (3H, t, *J* = 6.6 Hz, CH_3_), 1.25 (10H, m, (CH_2_)_5_), 1.45–1.50 (17H, m, 5 × CH_3_ and CH_2_), 2.87 (2H, s, CH_2_CO), 3.16 (2H, m, NCH_2_CH_2_), 4.39 (2H, m, NCH_2_Ar), 7.20 (1H, dd, *J* = 8.1, 0.4 Hz, ArH), 7.32 (1H, br. d, ArH), 7.97 (1H, br. d, ArH).

#### *2,2-Dimethyl-6-((octylamino)methyl)thiochroman-4-one 1-oxide* (**13**)

To compound **26** (27 mg, 0.06 mmol) in methanol (1 mL) was added 35% H_2_O_2_ (11.3 mg solution, 0.1 mmol) and Montmorillonite K10 (20 mg). The resulting mixture was stirred at room temperature overnight, after which the clay was removed by filtration. The solvent was evaporated to yield BOC-sulfoxide **27a** (25 mg, 89%). ^1^H-NMR (CDCl_3_,-) δ_H_ 0.88 (3H, t, *J* = 6.9 Hz, CH_3_), 1.26 (10H, m, (CH_2_)_5_), 1.41–1.54 (17H, m, 5 × CH_3_ and CH_2_), 2.79 (1H of CH_2_CO), 3.22 (3H, m, 1H of CH_2_CO and NCH_2_CH_2_), 4.52 (2H, m, NCH_2_Ar), 7.68 (1H, br., ArH), 7.90 (1H, br., ArH), 8.01 (1H, br. d, ArH). To a solution of **27a** in DCM (2mL) at 0 °C was added trifluoroacetic acid (2 mL). The reaction was stirred overnight, after which time the reaction was washed with saturated sodium bicarbonate solution and extracted with DCM (3 × 10 mL). The combined organic layers were dried over anhydrous Na_2_SO_4_, evaporated and purified by flash column chromatography (pet. ether/EtOAc 3:1) to yield the deprotected sulfoxide **13**. ^1^H-NMR (CDCl_3_-) δ_H_ 0.80 (3H, t, *J* = 6.7 Hz, CH_3_), 1.19 (12H, m, (CH_2_)_6_), 1.48 (6H, s, (CH_3_)_2_, 2.54 (2H, t, *J* = 7.2 Hz, NCH_2_CH_2_), 3.17 (2H, s, CH_2_CO), 3.85 (2H, s, NCH_2_Ar), 7.78 (1H, d, *J* = 8.1 Hz, ArH), 7.95 (1H, d, *J* = 7.8 Hz, ArH), 7.98 (1H, s, ArH); ^13^C-NMR δ_C_ 14.1 (CH_3_), 21.1 ((CH_3_)_2_), 22.7, 27.3, 29.3, 29.5, 29.9, 31.8, 49.4, 50.6, 53.0 (9 × CH_2_), 58.7 (C(CH_3_)_2_), 125.2 (ArCH), 127.5 (ArCH), 130.5 (ArC), 134.7 (ArCH), 137.4 (ArC), 146.5 (ArC), 190.8 (C=O).

#### *2,2-Dimethyl-6-((octylamino)methyl)thiochroman-4-one 1,1-dioxide* (**14**)

To a suspension of **26** (40 mg, 0.09 mmol) in glacial acetic acid (10 mL) was added sodium perborate·4H_2_O (30 mg, 0.2 mmol), in portions. After stirring at 55 °C for 4 h, the reaction was poured into ice/water (100 mL). Ethyl acetate was added and the aqueous layer extracted (3 × 50 mL). The organic layers were combined, the solvent removed in vacuo and the residue purified by flash chromatography (pet. ether/EtOAc 8:1) to give the BOC-sulfone **27b** (33 mg, 77%). ^1^H-NMR (CDCl_3_) δ_H_ 0.87 (3H, t, *J* = 6.8 Hz, CH_3_), 1.26 (10H, m, (CH_2_)_5_), 1.37–1.55 (17H, m, 5 × CH_3_ and CH_2_), 3.14–3.25 (4H, m, CH_2_CO and NCH_2_CH_2_), 4.52 (2H, m, NCH_2_Ar), 7.71 (1H, br., ArH), 7.95 (1H, br., ArH), 8.03 (1H, d, *J* = 8.1 Hz, ArH). To a solution of **27b** in DCM (2 mL) at 0 °C was added trifluoroacetic acid (2 mL). The reaction was stirred overnight, after which time the reaction was washed with saturated sodium bicarbonate solution and extracted with DCM (3 × 10 mL). The combined organic layers were dried over anhydrous Na_2_SO_4_, evaporated and purified by flash column chromatography (pet. ether/EtOAc 3:1) to yield the deprotected sulfone **14** (18 mg, 69%). ^1^H-NMR (CDCl_3_) δ_H_ 0.87 (3H, t, *J* = 6.6 Hz, CH_3_), 1.26 (12H, m, (CH_2_)_6_), 1.53 (6H, s, (CH_3_)_2_, 2.64 (2H, t, *J* = 7.3 Hz, NCH_2_CH_2_), 3.24 (2H, s, CH_2_CO), 3.95 (2H, s, NCH_2_Ar), 7.87 (1H, d, *J* = 8.2 Hz, ArH), 8.03 (1H, d, *J* = 7.9 Hz, ArH), 8.06 (1H, s, ArH); ^13^C- NMR δ_C_ 14.1 (CH_3_), 21.1 ((CH_3_)_2_), 22.7, 27.2, 29.2, 29.4, 29.5, 31.8, 49.2, 50.6, 52.7 (9 × CH_2_), 58.7 (C(CH_3_)_2_), 125.2 (ArCH), 127.7 (ArCH), 130.6 (ArC), 134.9 (ArCH), 137.7 (ArC), 145.5 (ArC), 190.8 (C=O).

### 4.2. Bioassay Procedures: Antileishmanial Activity on L. infantum Axenic Amastigotes

*L. infantum* promastigotes (MHOM/MA/67/ITMAP-263, CNR Leishmania, Montpellier, France, expressing luciferase activity) were cultivated in RPMI 1640 medium supplemented with 10% foetal calf serum (FCS), 2 mM L-glutamine and antibiotics (100 U/mL penicillin and 100 μg/mL streptomycin) and harvested in the logarithmic phase of growth by centrifugation at 900× *g* for 10 min. The supernatant was carefully removed and replaced by the same volume of RPMI 1640 complete medium at pH 5.4, and then incubated for 24 h at 24 °C. The acidified promastigotes were then incubated for 24 h at 37 °C in a ventilated flask to transform promastigotes into axenic amastigotes. The effects of the tested compounds on the growth of *L. infantum* axenic amastigotes were assessed as follows. *L. infantum* amastigotes were incubated at a density of 2 × 10^6^ parasites/mL in sterile 96-well plates with various concentrations of compounds dissolved in DMSO (final concentration less than 0.5% *v/v*), in duplicate. Appropriate controls, DMSO and amphotericin, were added to each set of experiments. After a 48 h incubation period at 37 °C, each plate-well was then microscopically examined to detect any precipitate formation. To estimate the luciferase activity of axenic amastigotes, 80 μL of each well were transferred to white 96-well plates, Steady Glow^®^ reagent (Promega, Charbonnières-les-Bains, France) was added according to the manufacturer’s instructions, and plates were incubated for 2 min. The luminescence was measured using a Microbeta Luminescence Counter (Perkin Elmer, Villebon-sur-Yvette, France). The inhibitory concentration 50% (IC_50_) was defined as the concentration of drug required to inhibit by 50% the metabolic activity of *L. infantum* amastigotes compared to control. IC_50_ values were calculated by non-linear regression analysis on dose response curves, using the TableCurve 2D V5 software (Systat Software, San Jose, CA, USA). IC_50_ values represent the mean of three independent experiments.

### 4.3. Cytotoxicity Evaluation on J774A.1 Cells

Evaluation of the cytotoxicity of test compounds was performed by MTT assay using the J774A.1 cell line (mouse macrophage cell line, Sigma-Aldrich). Briefly, cells (5 × 10^4^ cells/mL) in 100 µL of complete medium, [DMEM High glucose supplemented with 10% fetal calf serum (FCS), 2 mM L-glutamine and antibiotics (100 U/mL penicillin and 100 µg/mL streptomycin)] were seeded into each well of 96-well plates and incubated at 37 °C in a humidified 5% CO_2_ with 95% air atmosphere. After a 24 h incubation, 100 µL of medium with various product concentrations and appropriate controls were added and the plates were incubated for 72 h at 37 °C. Each plate-well was then examined under the microscope to detect possible precipitate formation before the medium was aspirated from the wells. 100 µL of MTT solution (0.5 mg/mL in RPMI) was then added to each well. Cells were incubated for 2 h at 37 °C. After this time, the MTT solution was removed and DMSO (100 µL) was added to dissolve the resulting formazan crystals. Plates were shaken vigorously (300 rpm) for 5 min. The absorbance was measured at 570 nm with a microplate spectrophotometer. DMSO was used as blank and doxorubicin (Sigma Aldrich, Saint-Quentin-Fallavier, France) as positive control. CC_50_ values were calculated by non-linear regression analysis on dose–response curves, using the TableCurve 2D V5 software.

## Figures and Tables

**Table 1:** Calculations of molecular properties of synthesised compounds using Molinspiration.

Compound	miLog P	TPSA	nAtoms	MW	nON	nOHNH	nRotb	Vol. (A^3^)
**10a**	4.91	43.38	26	366.48	3	0	6	337.14
**10b**	5.36	61.84	30	422.50	5	0	6	371.68
**11a**	4.26	46.17	26	365.50	3	1	5	340.56
**11b**	5.28	46.17	28	393.55	3	1	7	374.16
**11c**	4.40	64.64	30	421.52	5	1	5	375.10
**11d**	5.10	46.17	24	347.52	3	1	8	342.30
**11e**	5.45	46.17	26	440.45	3	1	10	377.23
**12a**	3.76	29.10	19	277.43	2	1	5	272.91
**12b**	4.77	29.10	21	305.49	2	1	7	306.51
**12c**	5.78	29.10	23	333.54	2	1	9	340.12
**12d**	6.79	29.10	25	361.60	2	1	11	373.72
**13**	4.19	46.17	24	349.54	3	1	9	347.67
**14**	4.66	63.24	25	365.54	4	1	9	353.42
**3**	5.40	38.33	23	317.47	3	1	9	330.98

**Table 2:** Activity of thiochromanones against *Leishmania* amastigotes and evaluation of cytotoxicity.

Compound	*L. infantum* IC_50_ (μM) ^1^	J774A.1 CC_50_ (μM) ^1^	S.I. ^2^
**10a**	34.4 (2.76)	>136.43	>3.97
**10b**	91.3 (0.47)	22.53 (2.32)	0.25
**11a**	10.5 (1.12)	31.63 (1.56)	3.01
**11b**	19.9 (2.01)	14.03 (1.63)	0.71
**11c**	>118.62	>118.62	ND ^3^
**11d**	51.3 (3.45)	137.46 (6.68)	2.68
**11e**	7.20 (0.34)	58.01 (2.31)	8.06
**12a**	26.5 (0.79)	33.23 (3.97)	1.25
**12b**	23.1 (0.85)	25.76 (2.42)	1.12
**12c**	19.5 (1.41)	22.64 (1.68)	1.16
**12d**	12.4 (0.88)	23.12 (0.33)	1.86
**13**	33.4 (2.72)	40.91 (3.06)	1.22
**14**	33.8 (1.56)	42.76 (5.96)	1.27
Amphotericin B	0.072 (0.011)		
Doxorubicin		0.047 (0.004)	

^1^ Results are reported as the mean value ± standard deviation of the half-maximum concentration in µM. ^2^ SI (Selectivity Index) = CC_50_/IC_50_. ^3^ Non-determinable.

## Data Availability

All data used for the present study has been provided within the Supplementary Materials.

## Supplementary Materials

The following are available online. NMR spectra of synthesized compounds.

## Sample Availability

Samples of the compounds are available from the authors on request.

## References

[1] Maxfield L., Crane J.S. (2019). Leishmaniasis. StatPearls.

[2] Pramanik P.K., Alam M.N., Roy Chowdhury D., Chakraborti T. (2019). Drug Resistance in Protozoan Parasites: An Incessant Wrestle for Survival. J. Glob. Antimicrob. Resist..

[3] Do Nascimento A.M., Costa F.C., Thiemann O.H., de Oliveira D.C. (2007). Chromanones with Leishmanicidal Activity from Calea uniflora. Z. Naturforsch C..

[4] Castro H., Cruz T., de Aguiar Amaral P., da Silva Cardoso P., Alsaffar A., Farrell P., Tomas A.M., Barlow J.W. (2020). Synthesis and evaluation of novel chromanone and quinolinone analogues of uniflorol as anti-leishmanial agents. Heliyon.

[5] Varela M.T., Dias R.Z., Martins L.F., Ferreira D.D., Tempone A.G., Ueno A.K., Lago J.H., Fernandes J.P. (2016). Gibbilimbol analogues as antiparasitic agents—Synthesis and biological activity against *Trypanosoma cruzi* and *Leishmania (L.) infantum*. Bioorg. Med. Chem. Lett..

[6] De Oliveira A., Mesquita J.T., Tempone A.G., Lago J.H., Guimarães E.F., Kato M.J. (2012). Leishmanicidal activity of an alkenylphenol from Piper malacophyllum is related to plasma membrane disruption. Exp. Parasitol..

[7] Varela M.T., Romaneli M.M., Lima M.L., Borborema S.E.T., Tempone A.G., Fernandes J.P.S. (2018). Antiparasitic activity of new gibbilimbol analogues and SAR analysis through efficiency and statistical methods. Eur. J. Pharm. Sci..

[8] Zhong Y., Han X., Li S., Qi H., Song Y., Qiao X. (2017). Design, Synthesis, Antifungal Activity and Molecular Docking of Thiochroman-4-one Derivatives. Chem. Pharm. Bull..

[9] Bang E.J., Noh S.G., Ha S., Jung H.J., Kim D.H., Lee A.K., Hyun M.K., Kang D., Lee S., Park C. (2018). Evaluation of the Novel Synthetic Tyrosinase Inhibitor (Z)-3-(3-bromo-4-hydroxybenzylidene)thiochroman-4-one (MHY1498) In Vitro and In Silico. Molecules.

[10] Song J., Jones L.M., Kishore Kumar G.D., Conner E.S., Bayeh L., Chavarria G.E., Charlton-Sevcik A.K., Chen S.-E., Chaplin D.J., Trawick M.L. (2012). Synthesis and Biochemical Evaluation of Thiochromanone Thiosemicarbazone Analogues as Inhibitors of Cathepsin L.. ACS Med. Chem. Lett..

[11] Schröder J., Noack S., Marhöfer R.J., Mottram J.C., Coombs G.H., Selzer P.M. (2013). Identification of Semicarbazones, Thiosemicarbazones and Triazine Nitriles as Inhibitors of Leishmania mexicana Cysteine Protease CPB. PLoS ONE.

[12] Vargas E., Echeverri F., Vélez I.D., Robledo S.M., Quiñones W. (2017). Synthesis and Evaluation of Thiochroman-4-One Derivatives as Potential Leishmanicidal Agents. Molecules.

[13] Vargas E., Echeverri F., Upegui Y.A., Robledo S.M., Quiñones W. (2018). Hydrazone Derivatives Enhance Antileishmanial Activity of Thiochroman-4-ones. Molecules.

[14] Upegui Zapata Y.A., Echeverri F., Quiñones W., Torres F., Nacher M., Rivas L.I., dos Santos Meira C., Gedamu L., Escobar G., Archbold R. (2020). Mode of action of a formulation containing hydrazones and saponins against *leishmania* spp. Role in mitochondria, proteases and reinfection process. Int. J. Parasitol. Drugs Drug Resist..

[15] Ortiz C., Echeverri F., Robledo S., Lanari D., Curini M., Quiñones W., Vargas E. (2020). Synthesis and Evaluation of Antileishmanial and Cytotoxic Activity of Benzothiopyrane Derivatives. Molecules.

[16] Prakash G.K.S., Narayanan A., Nirmalchandar A., Vaghoo H., Paknia F., Mathew T., Olah G.A. (2017). Direct synthesis of 2-/3-(trifluoromethyl)thiochroman-4-ones: Superacid-induced tandem alkylation-cyclic acylation of benzenethiols using 2-/3-(trifluoromethyl)acrylic acid. J. Fluor. Chem..

[17] Vaghoo H., Prakash G.K.S., Narayanan A., Chaudhary R., Paknia F., Mathew T., Olah G.A. (2015). Superelectrophilic Activation of Crotonic/Methacrylic Acids: Direct Access to Thiochroman-4-ones from Benzenethiols by Microwave-Assisted One-Pot Alkylation/Cyclic Acylation. Org. Lett..

[18] Clayton S.E., Gabbutt C.D., Hepworth J.D., Heron B.M. (1993). Direct aromatic tert-butylation during the synthesis of thiochroman-4-ones. Tetrahedron.

[19] Lakouraj M.M., Tajbakhsh M., Tashakkorian H. (2007). Montmorillonite K10 Catalyzed Selective Oxidation of Sulfides to Sulfoxides Using Hydrogen Peroxide. Lett. Org. Chem..

[20] Page G.O. (1993). The Mild Oxidation of Some Alpha-Difluoro Sulfides to Sulfones with Sodium Perborate. Synth. Commun..

[21] Zhang C., Bourgeade Delmas S., Fernández Álvarez Á., Valentin A., Hemmert C., Gornitzka H. (2018). Synthesis, characterization, and antileishmanial activity of neutral N-heterocyclic carbenes gold(I) complexes. Eur. J. Med. Chem..

[22] Di Pisa F., Landi G., Dello Iacono L., Pozzi C., Borsari C., Ferrari S., Santucci M., Santarem N., Cordeiro-da-Silva A., Moraes C.B. (2017). Chroman-4-One Derivatives Targeting Pteridine Reductase 1 and Showing Anti-Parasitic Activity. Molecules.

[23] Omolabi K.F., Iwuchukwu E.A., Odeniran P.O., Soliman M.E.S. (2021). Could chroman-4-one derivative be a better inhibitor of PTR1? Reason for the identified disparity in its inhibitory potency in Trypanosoma brucei and Leishmania major. Comput. Biol. Chem..

