# Supplementary material for: Synthesis and Evaluation of Novel 2,2-Dimethylthiochromanones as Anti-Leishmanial Agents
Source: Molecules. 2021 Apr 12;26(8):2209. doi: 10.3390/molecules26082209 (PMC8069510; doi:10.3390/molecules26082209)
Supplement: Supplementary file 1 [file molecules-26-02209-s001.zip › Supplementary Information.docx]

**Supplementary Information : NMR spectra**

^1^H-NMR (400 MHz, CDCl_3_) spectrum of compound **16**

**
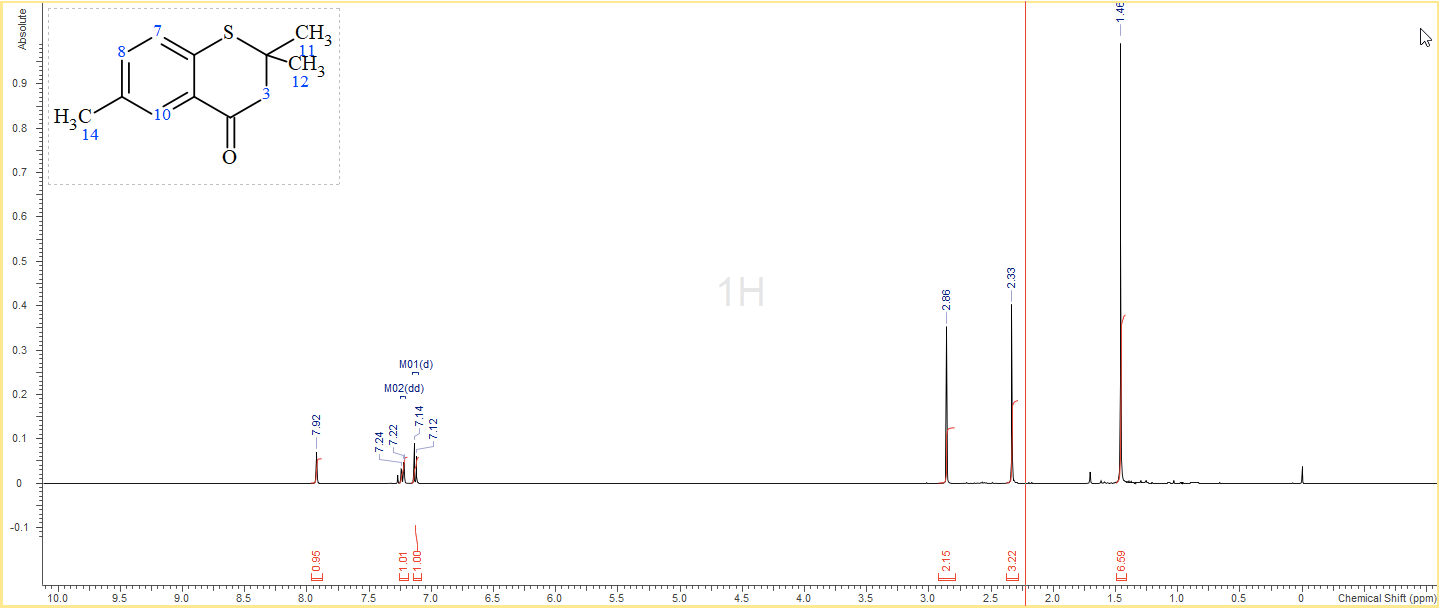
**

^13^C-NMR (101 MHz, CDCl_3_) spectrum of compound **16**

**
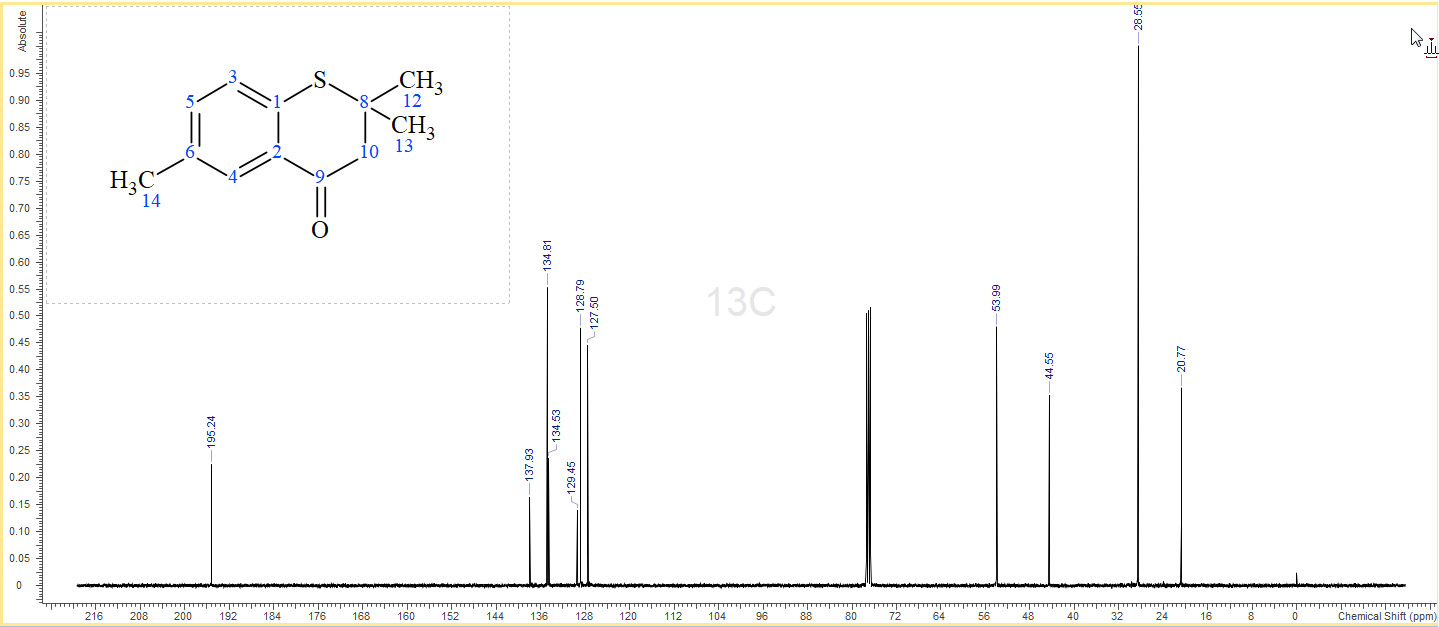
**

^1^H-NMR (400 MHz, CDCl_3_) spectrum of compound **17**

**
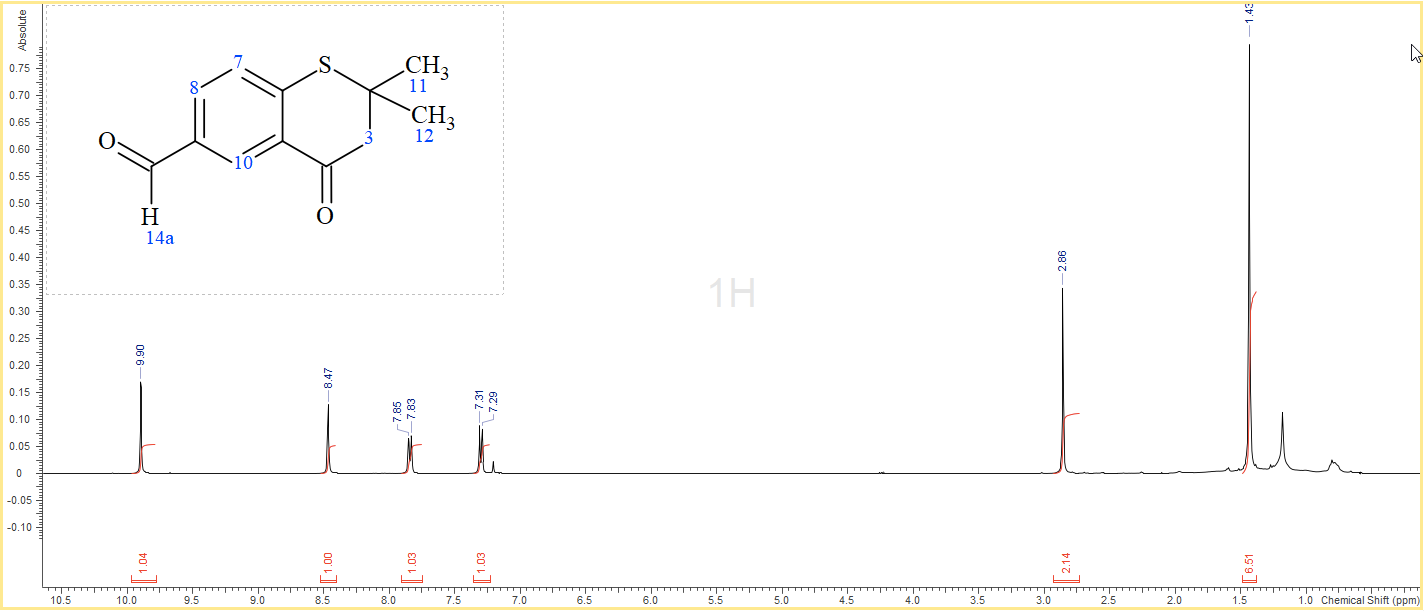
**

^13^C-NMR (101 MHz, CDCl_3_) spectrum of compound **17**

^
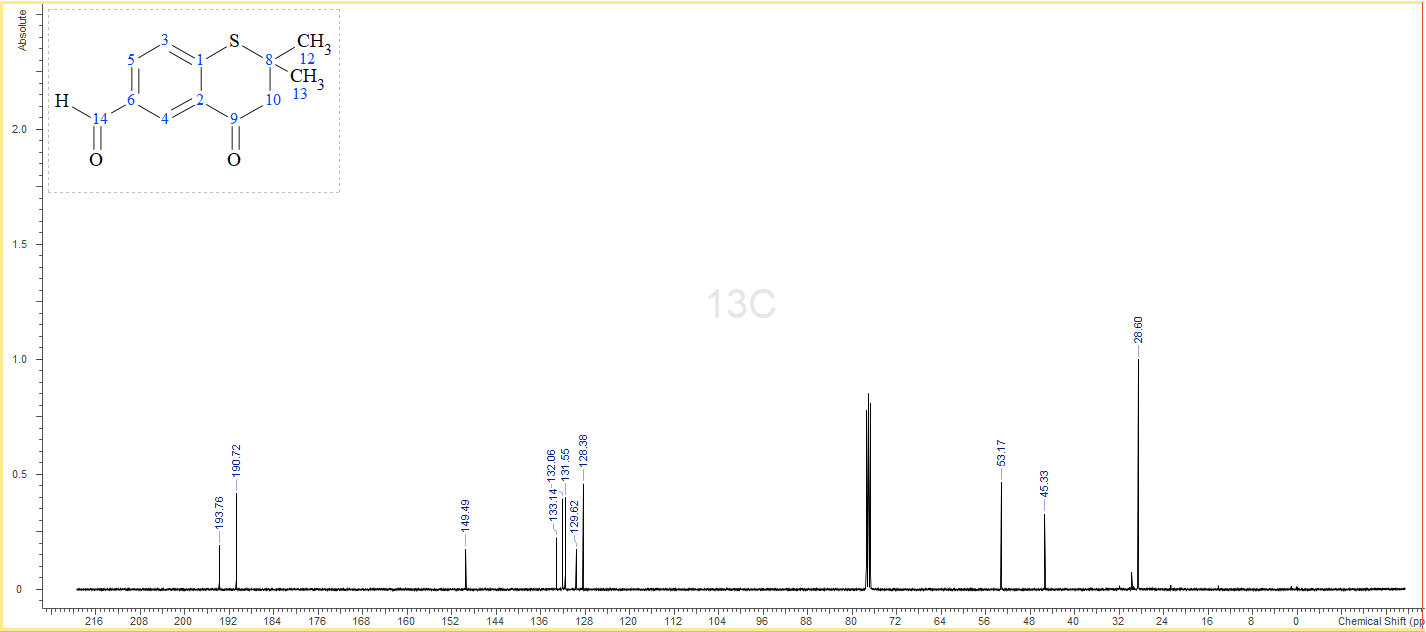
^

^1^H-NMR (400 MHz, CDCl_3_) spectrum of compound **18**

^
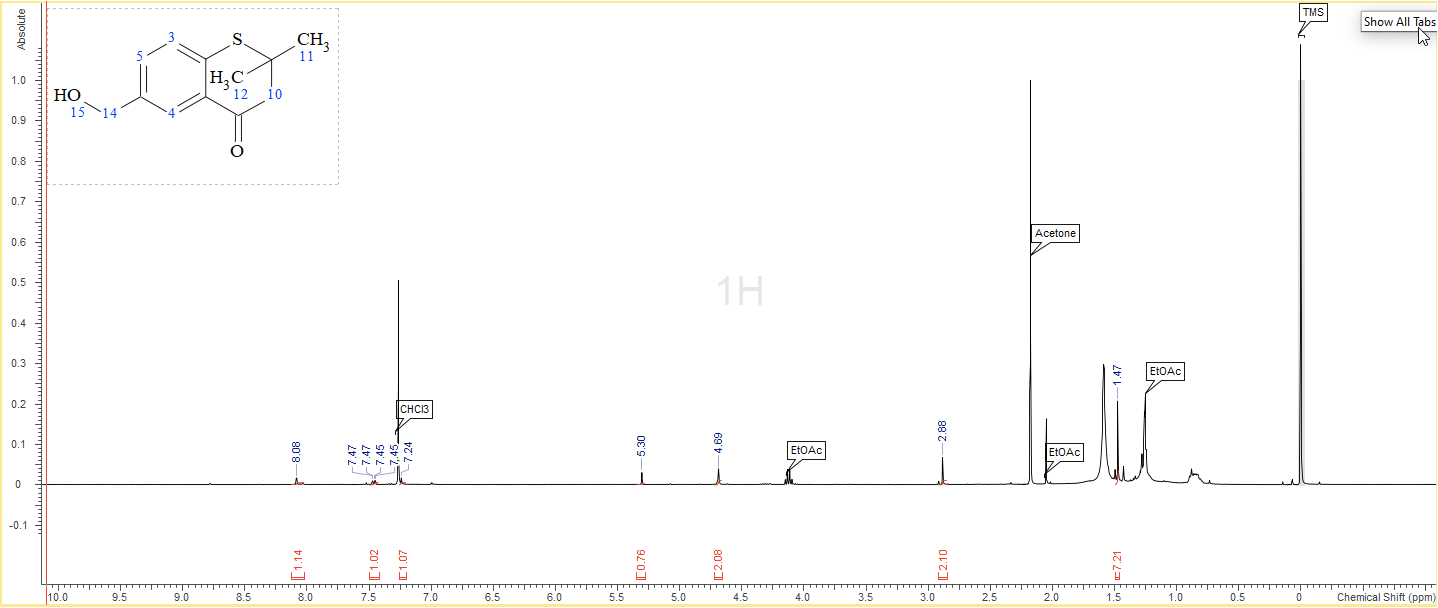
^

^1^H-NMR (400 MHz, CDCl_3_) spectrum of compound **10a**

**
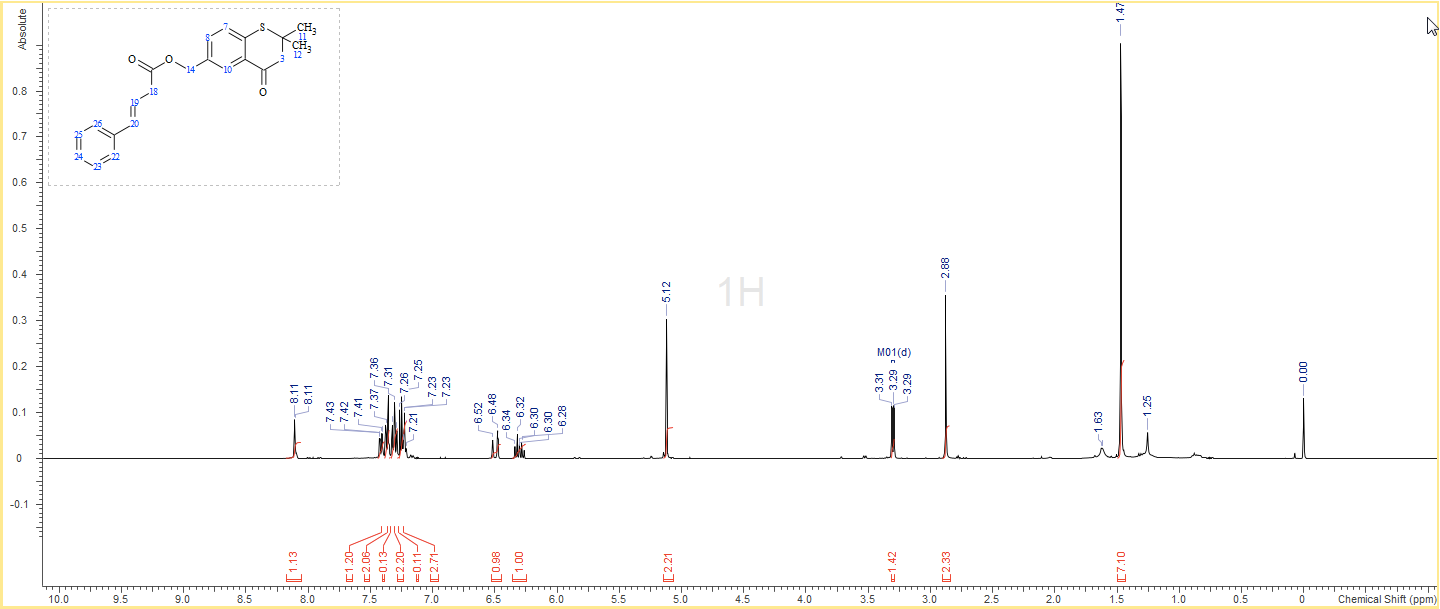
**

^13^C-NMR (101 MHz, CDCl_3_) spectrum of compound **10a**


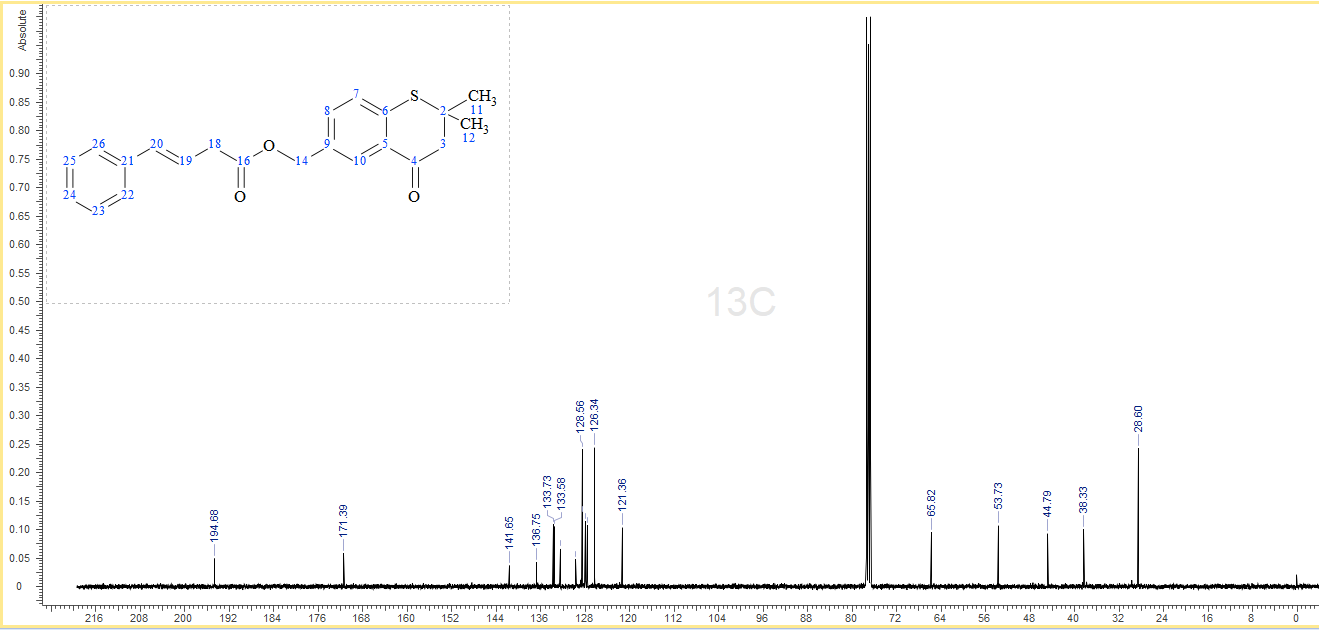


^1^H-NMR (400 MHz, CDCl_3_) spectrum of compound **10b**


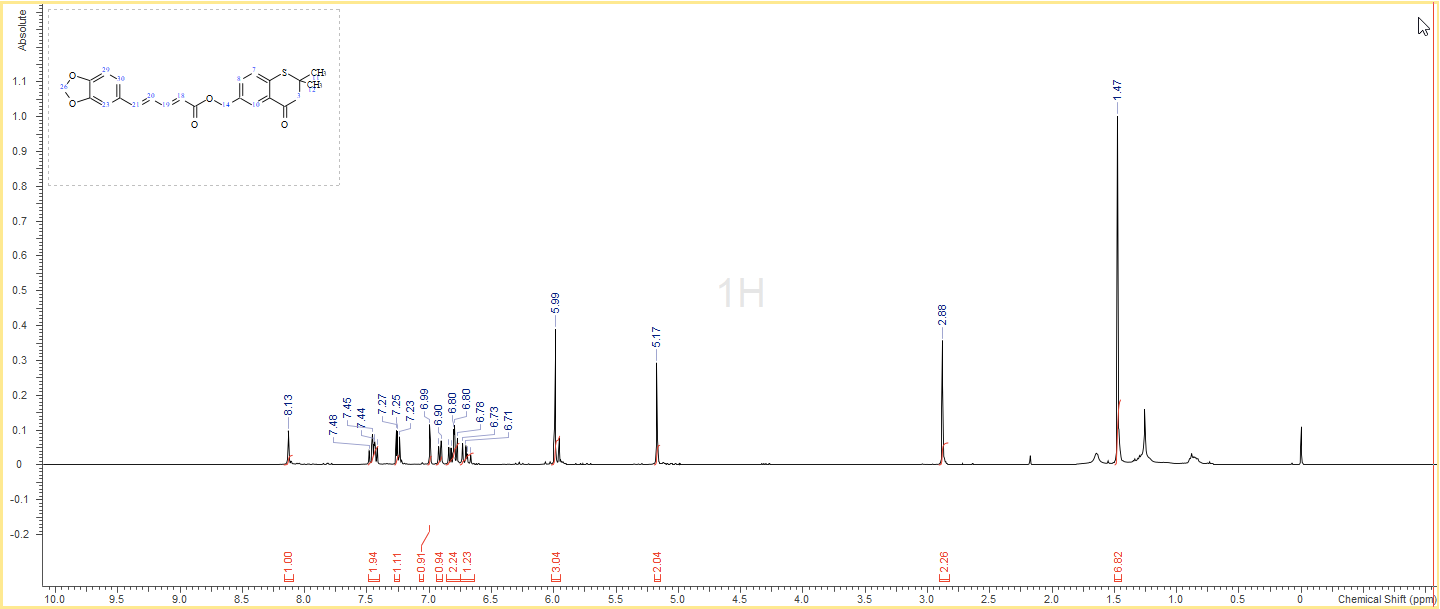


^13^C-NMR (101 MHz, CDCl_3_) spectrum of compound **10b**

^
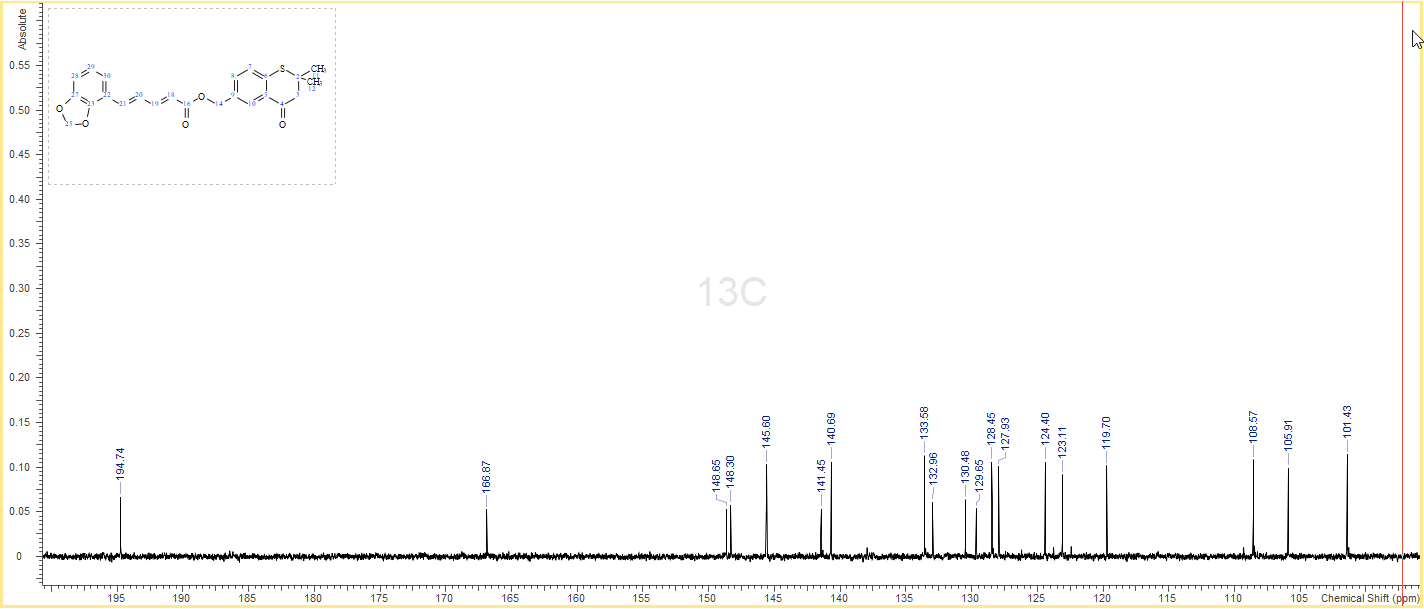
^

^1^H-NMR (400 MHz, CDCl_3_) spectrum of compound **19**

**
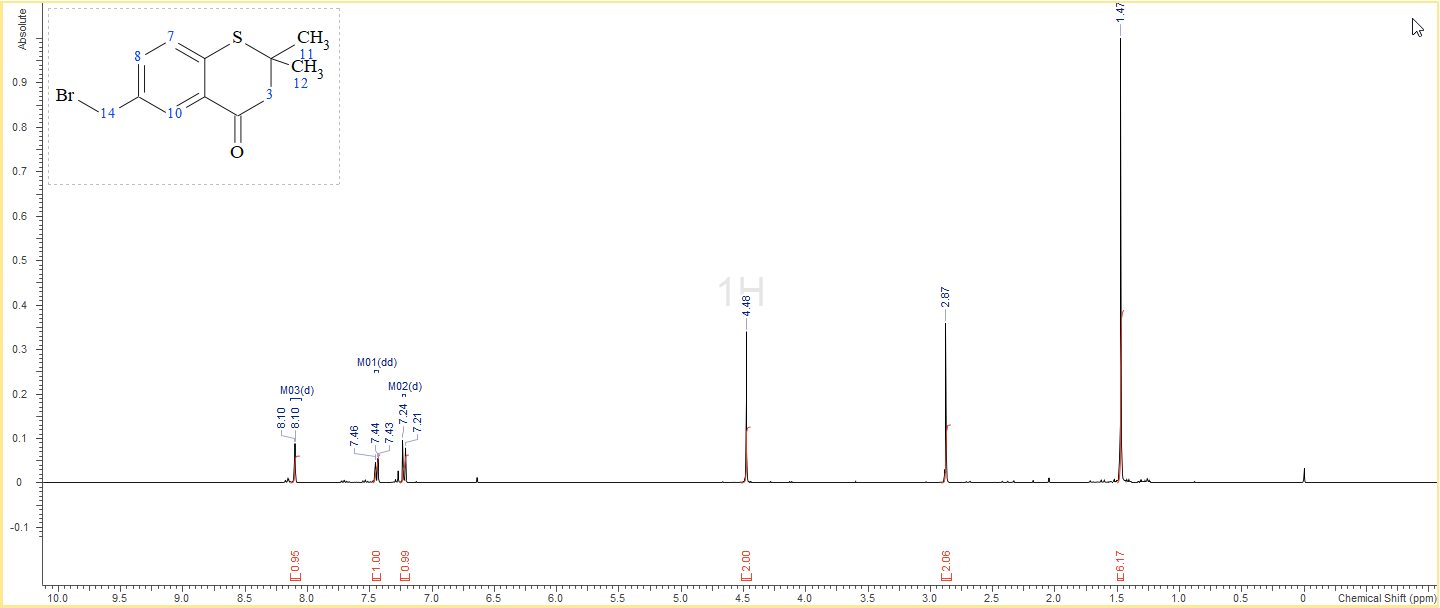
**

^13^C-NMR (101 MHz, CDCl_3_) spectrum of compound **19**

**
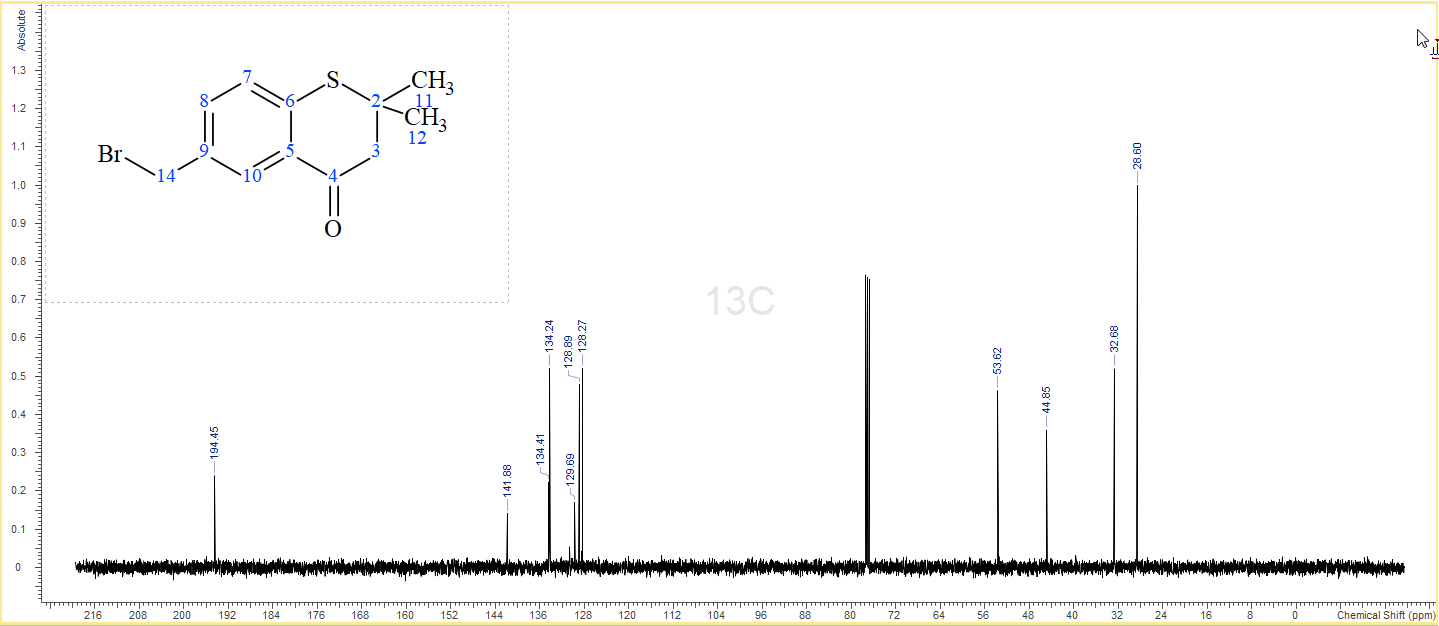
**

^1^H-NMR (400 MHz, CDCl_3_) spectrum of compound **20**

^
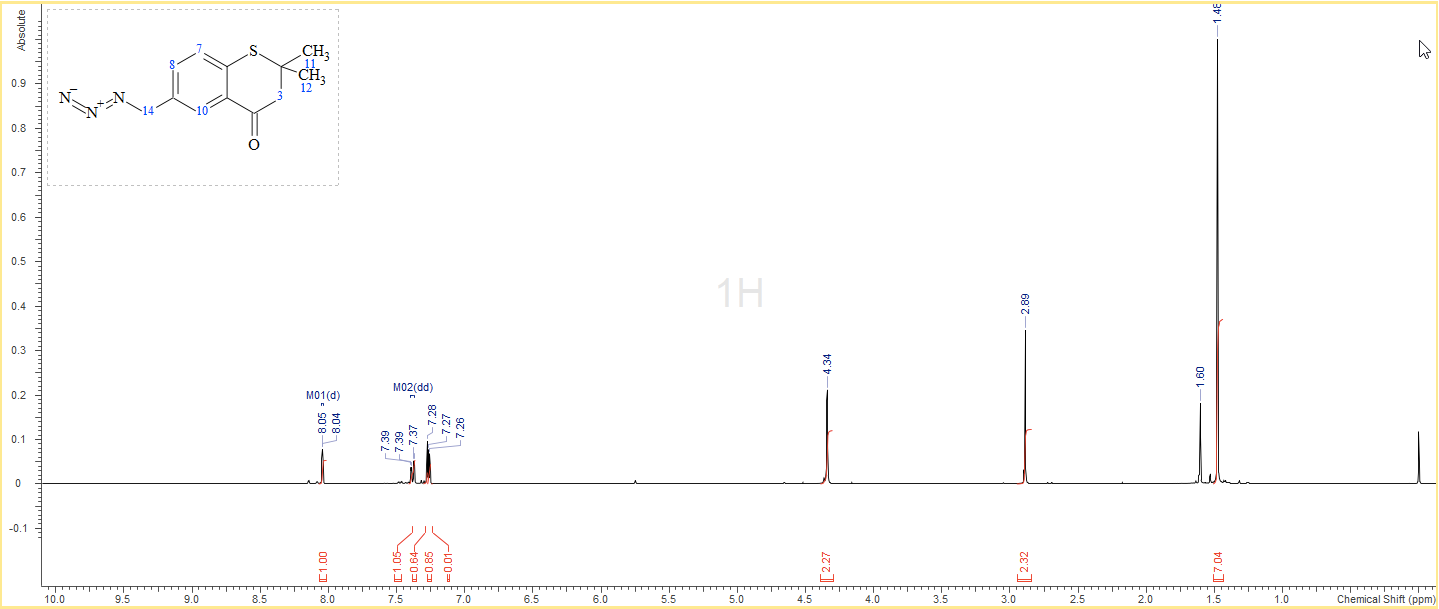
^

^13^C-NMR (101 MHz, CDCl_3_) spectrum of compound **20**

**
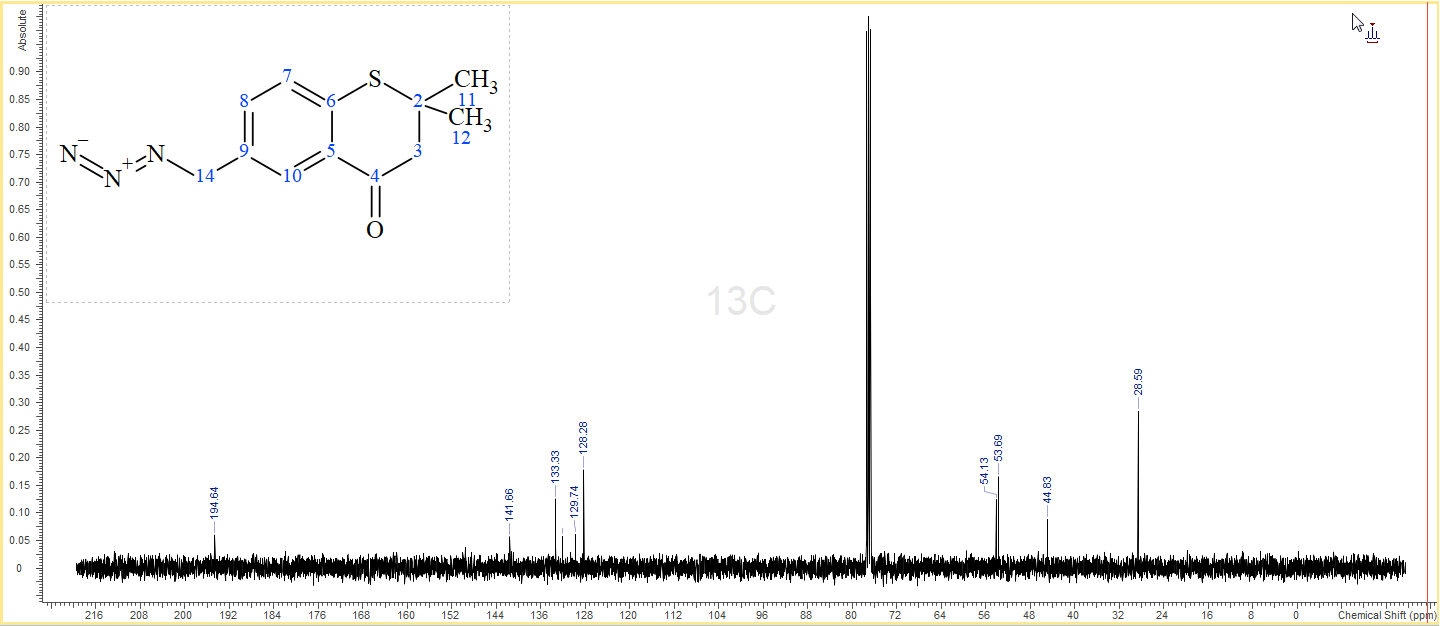
**

^1^H-NMR (400 MHz, CDCl_3_) spectrum of compound **21**

^
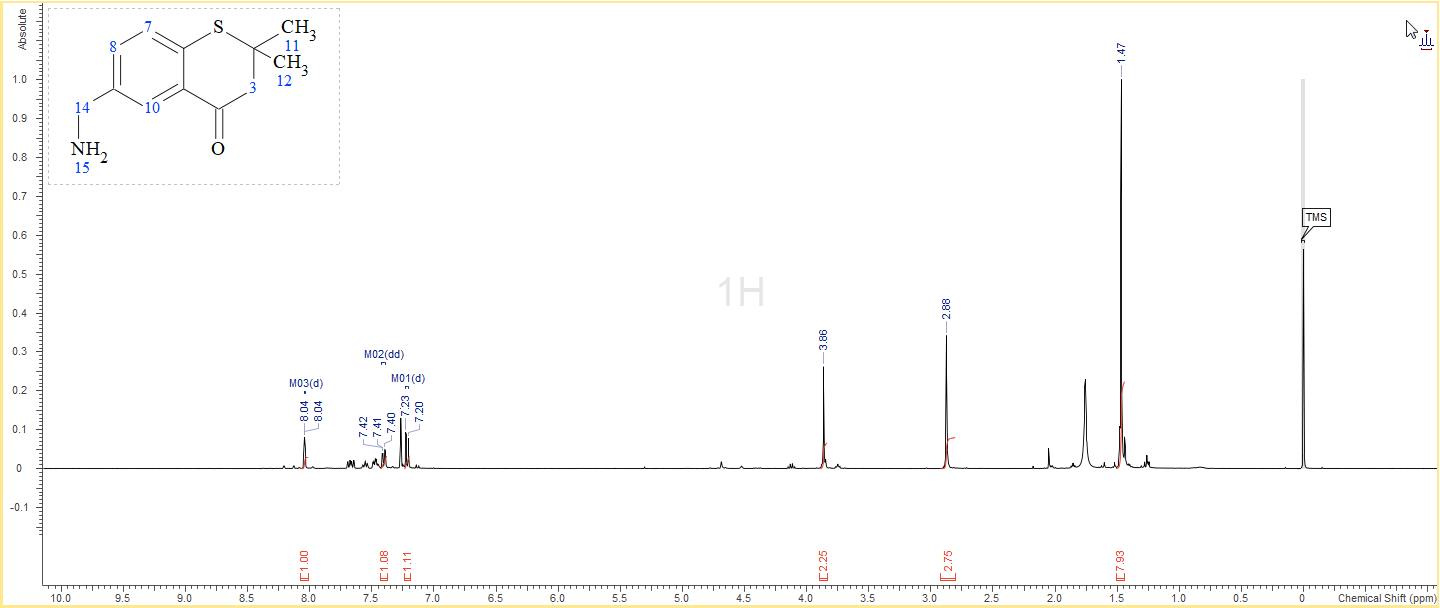
^

^13^C-NMR (101 MHz, CDCl_3_) spectrum of compound **21**

^
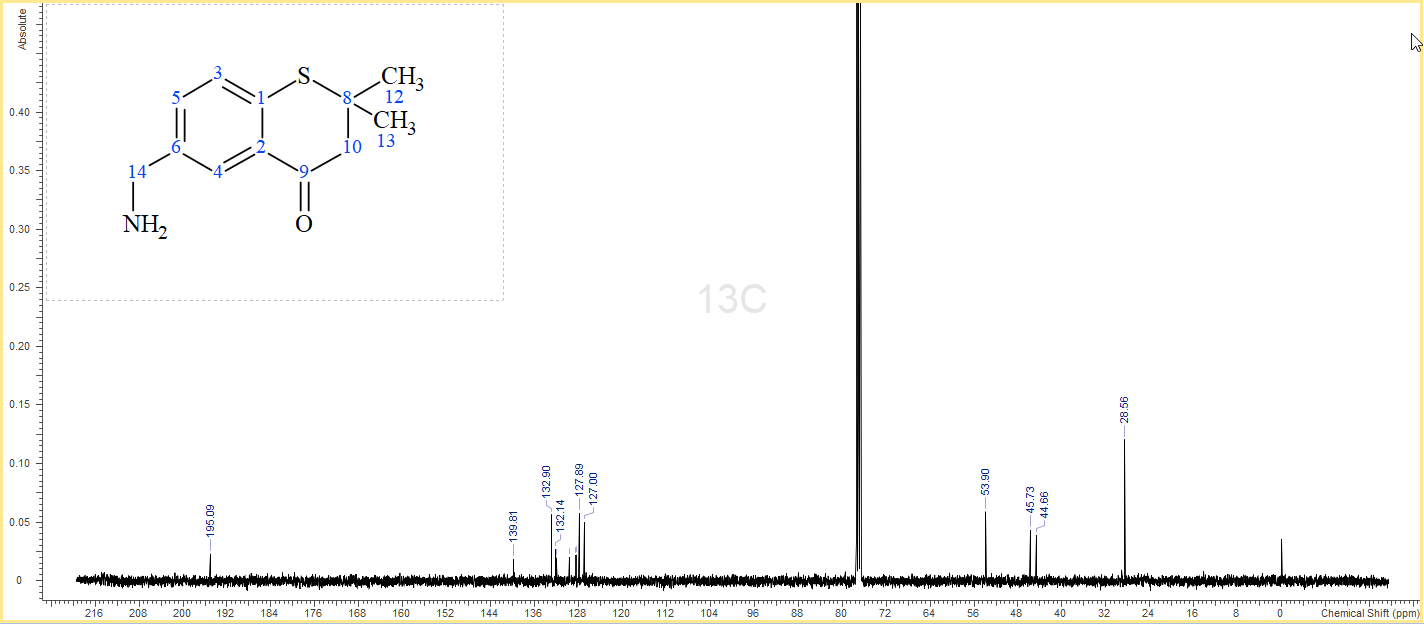
^

^1^H-NMR (400 MHz, CDCl_3_) spectrum of compound **11a**

^
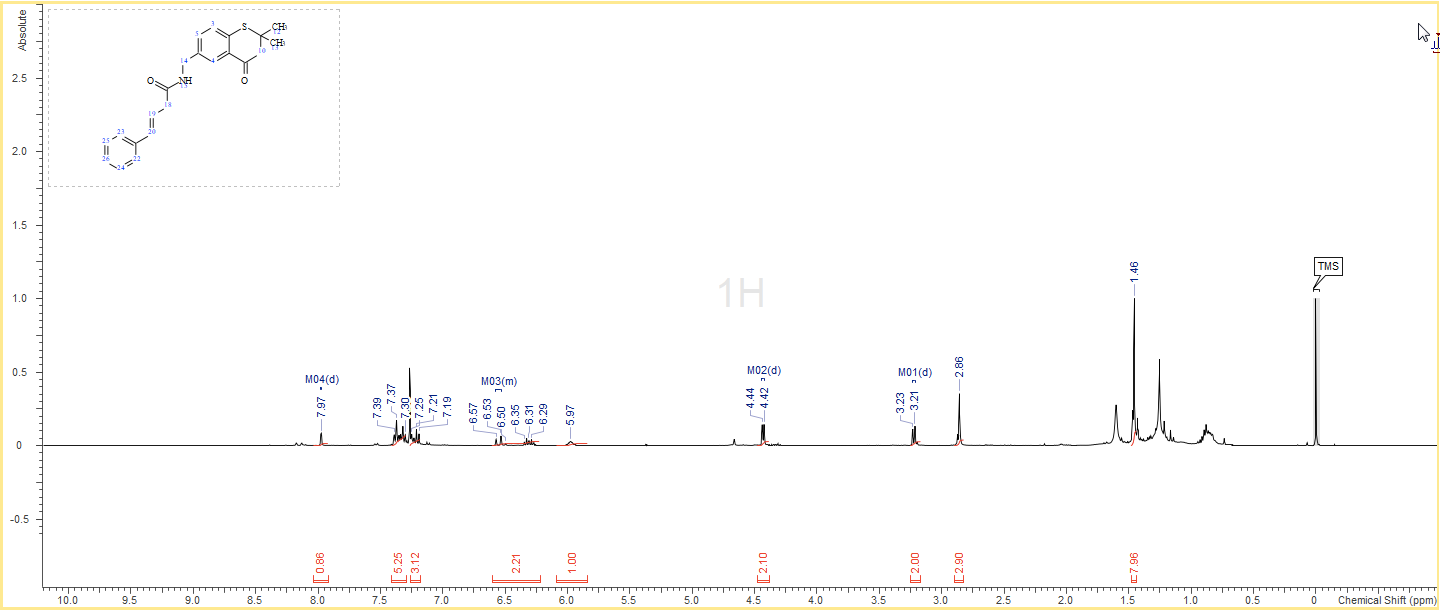
^

^13^C-NMR (101 MHz, CDCl_3_) spectrum of compound **11a**

**
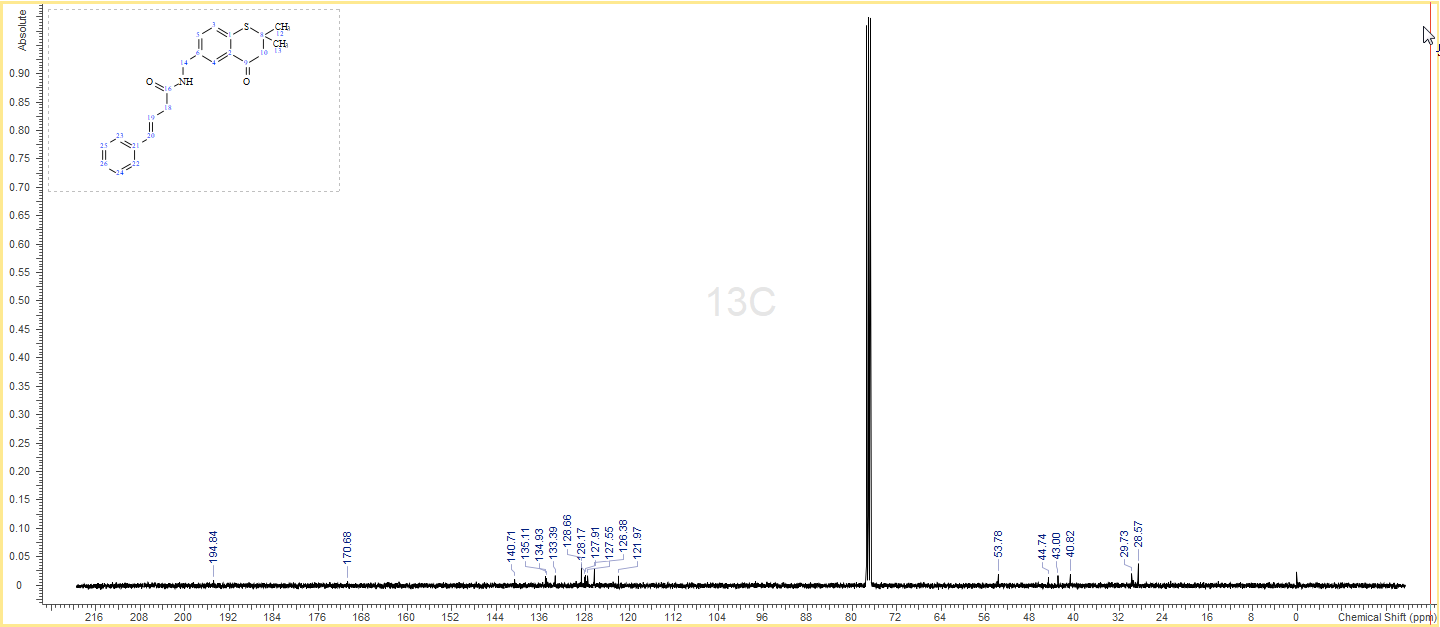
**

^1^H-NMR (400 MHz, CDCl_3_) spectrum of compound **11b**


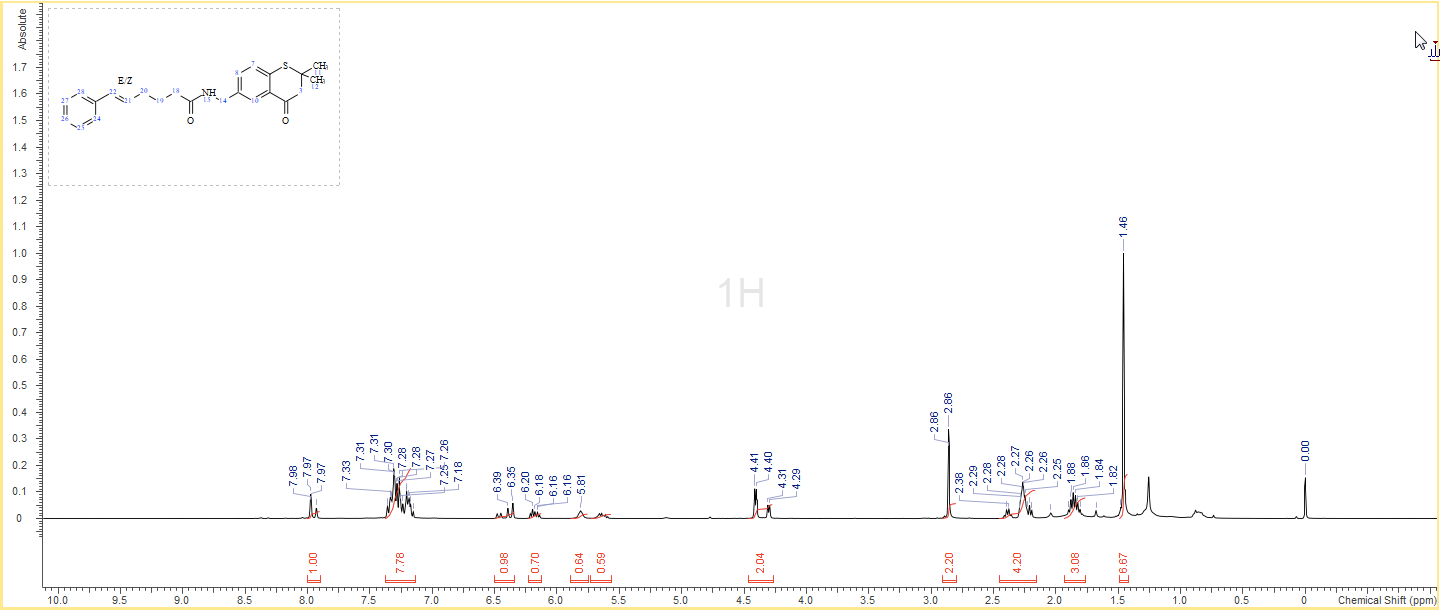


^13^C-NMR (101 MHz, CDCl_3_) spectrum of compound **11b**

^
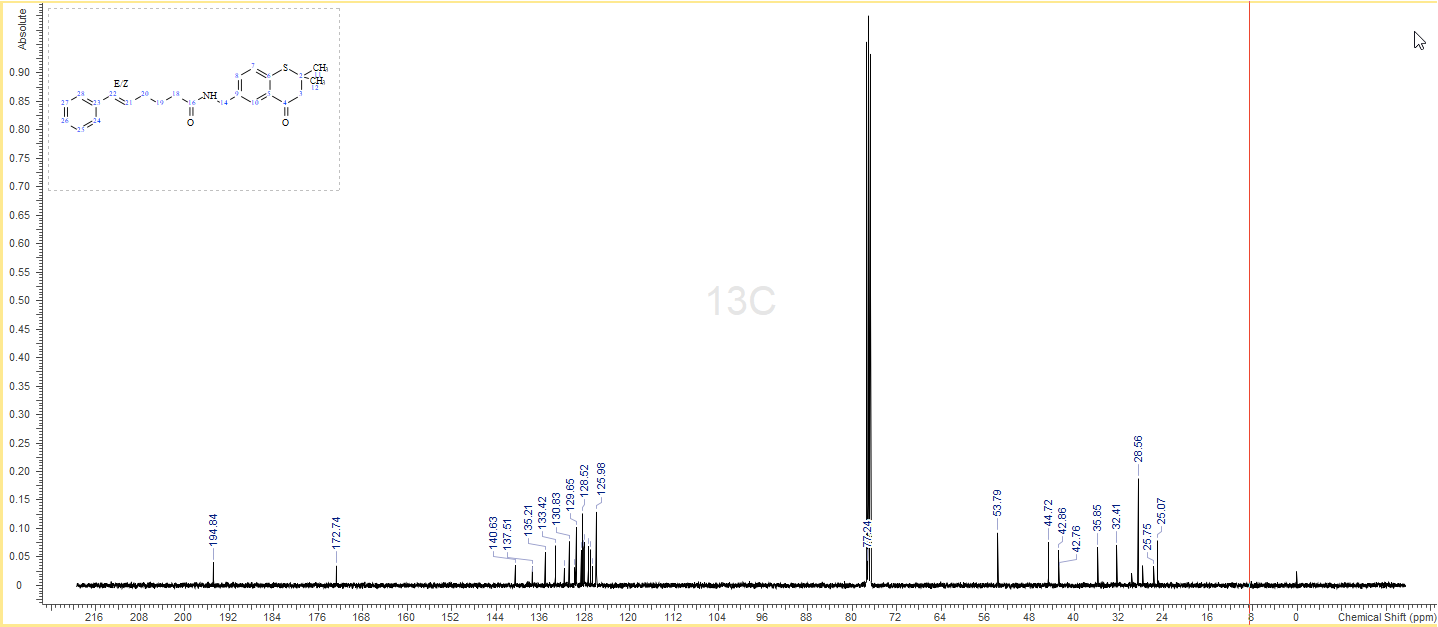
^

^1^H-NMR (400 MHz, CDCl_3_) spectrum of compound **11c**

^
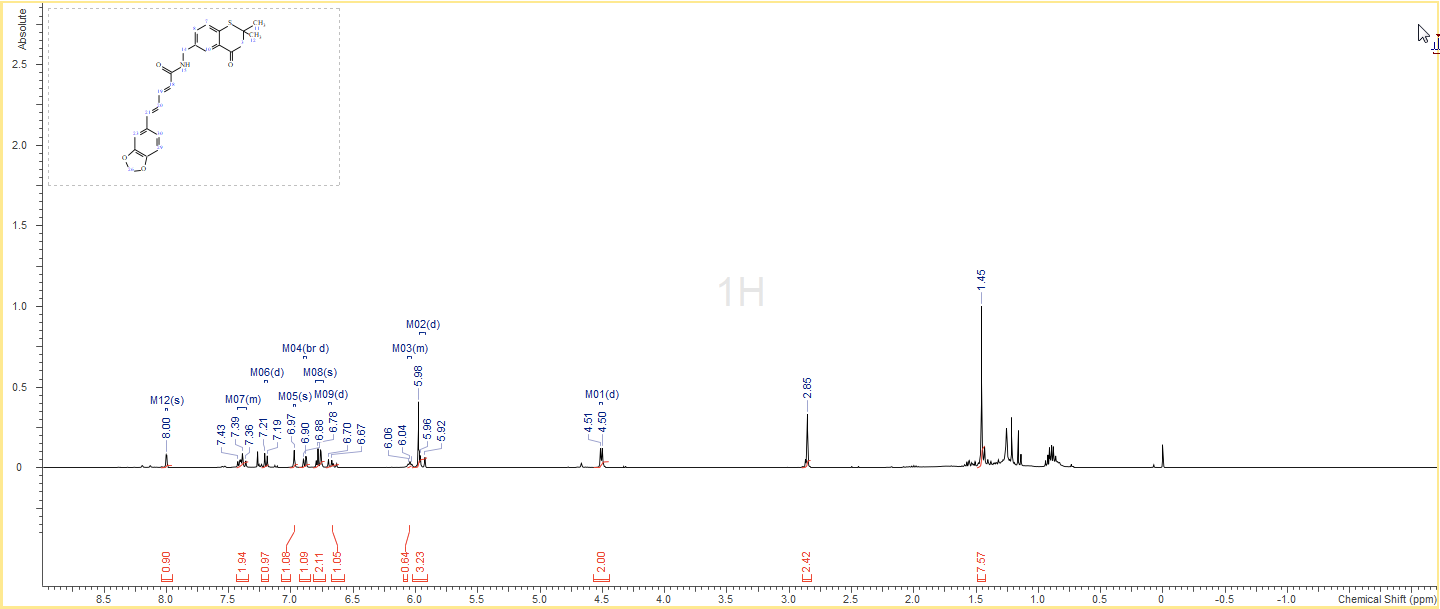
^

^13^C-NMR (101 MHz, CDCl_3_) spectrum of compound **11c**

**
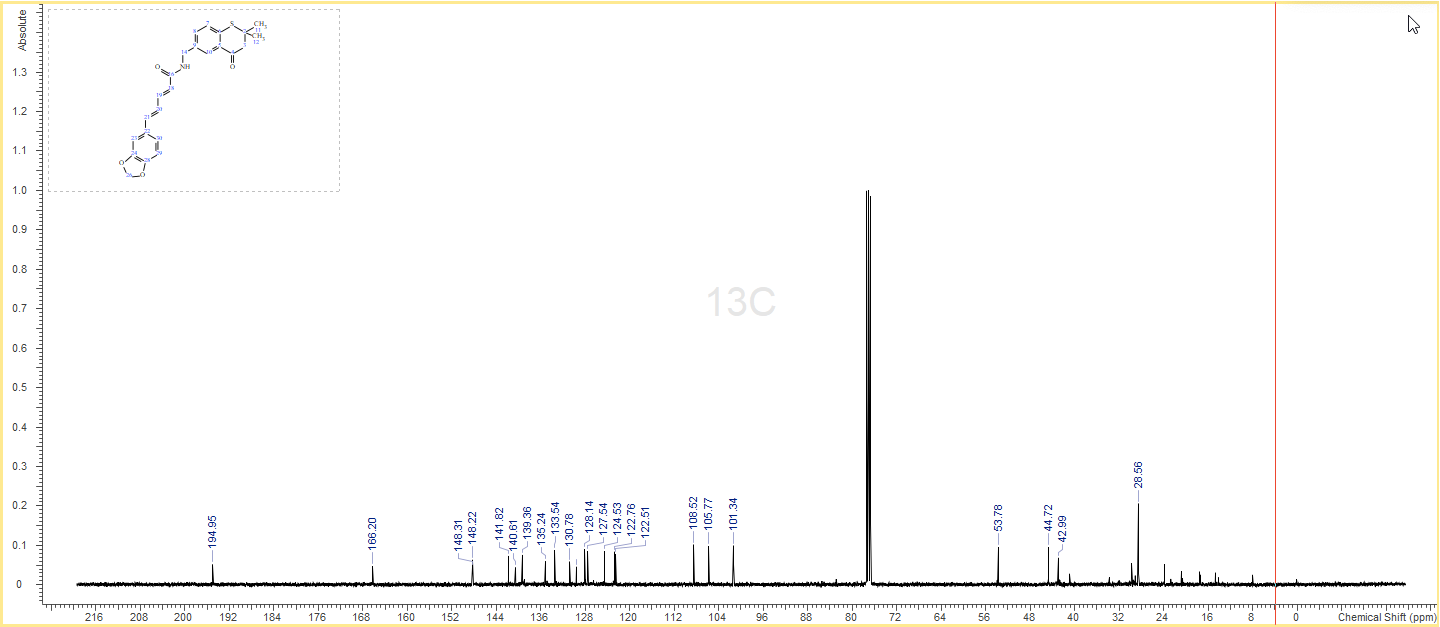
**

^1^H-NMR (400 MHz, CDCl_3_) spectrum of compound **11d**


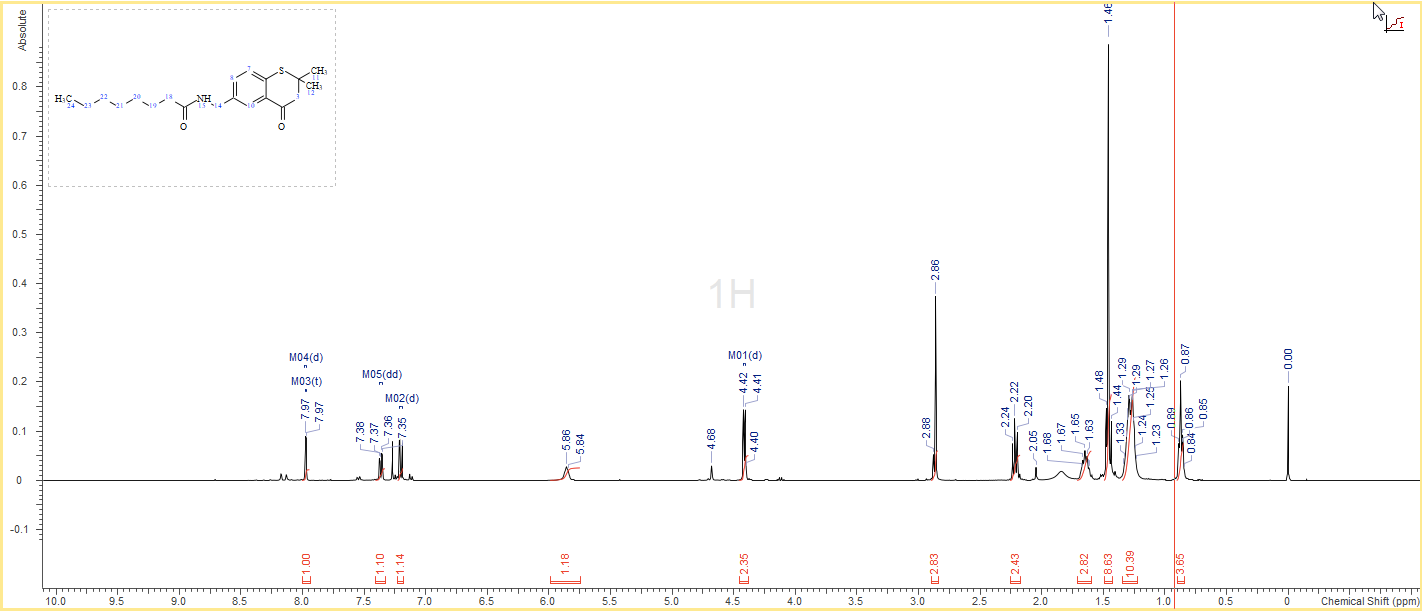


^13^C-NMR (101 MHz, CDCl_3_) spectrum of compound **11d**


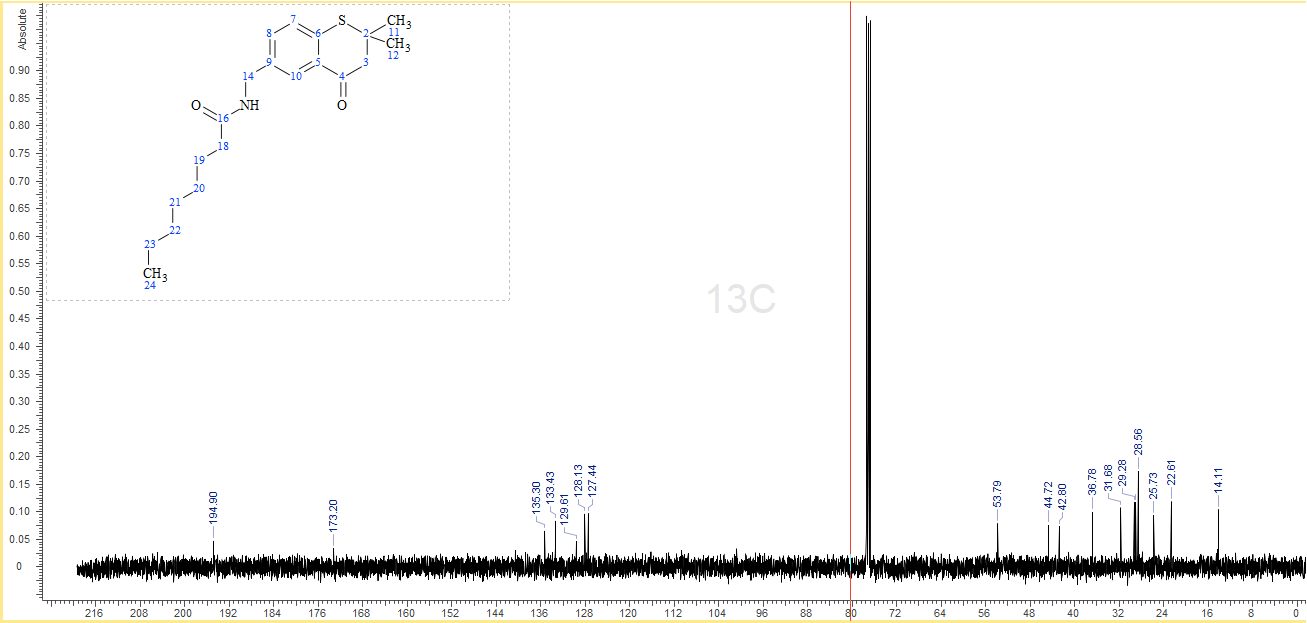


^1^H-NMR (400 MHz, CDCl_3_) spectrum of compound **11e**

^
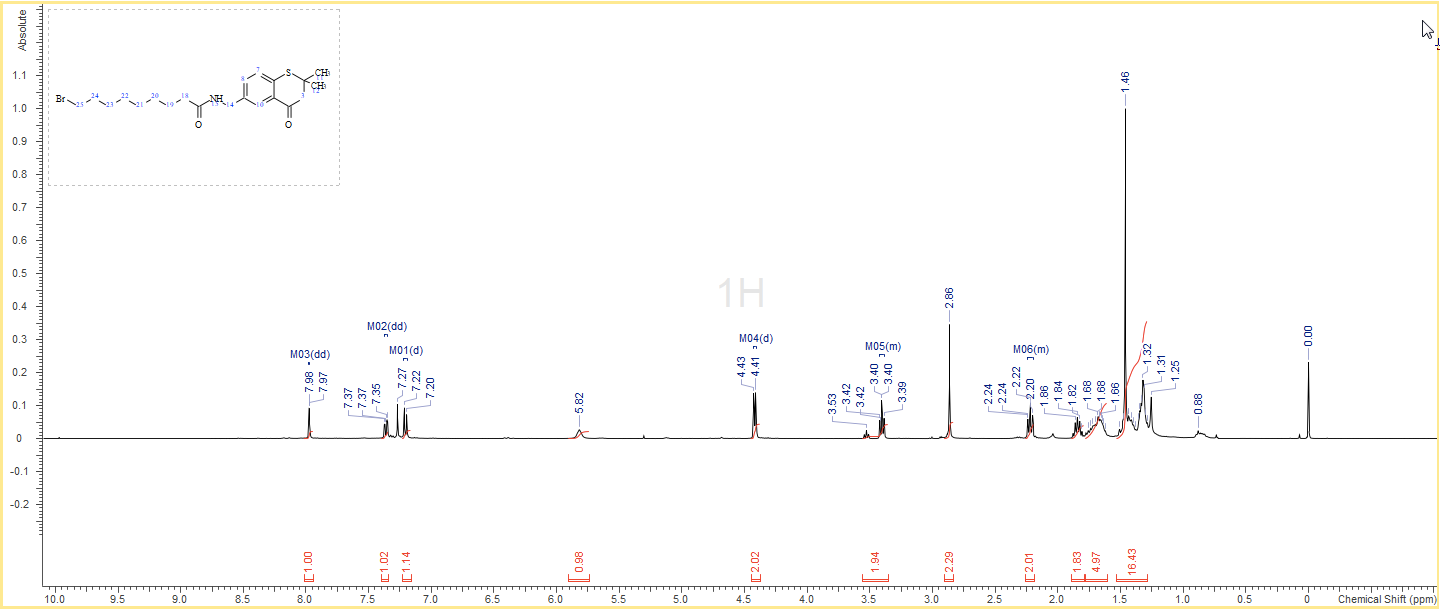
^

^13^C-NMR (101 MHz, CDCl_3_) spectrum of compound **11e**

^
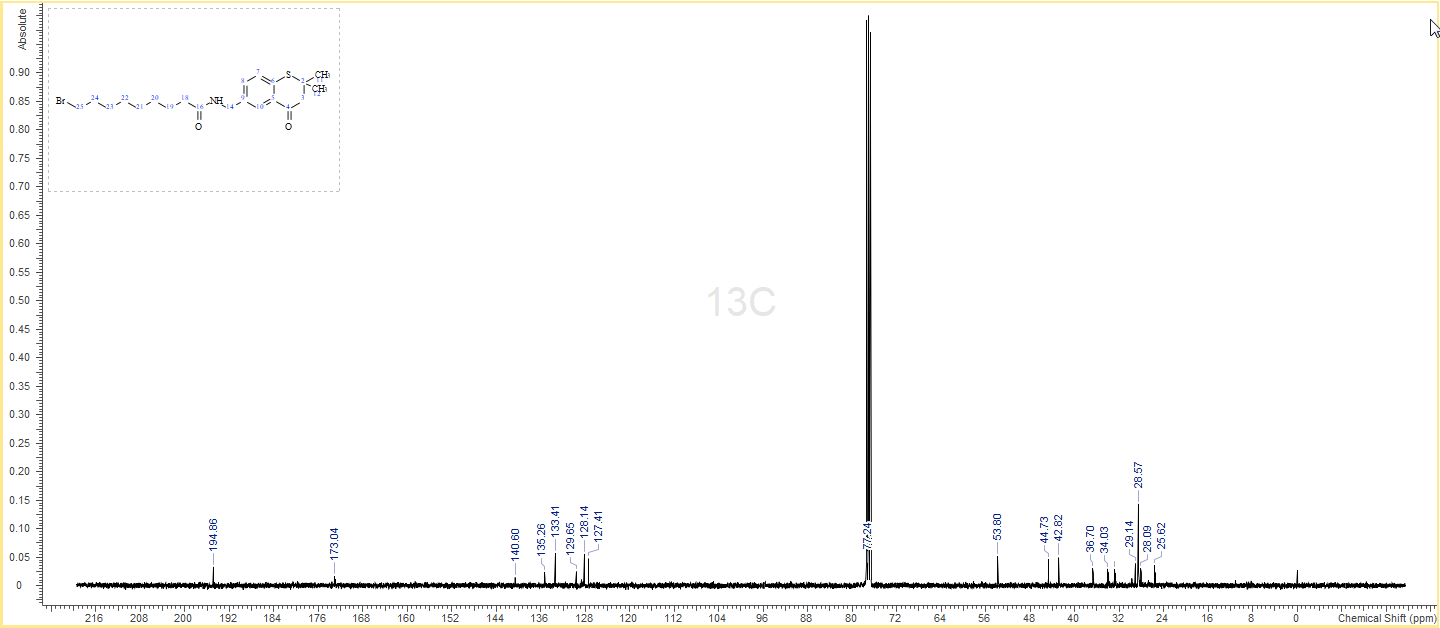
^

^1^H-NMR (400 MHz, CDCl_3_) spectrum of compound **22a**

^
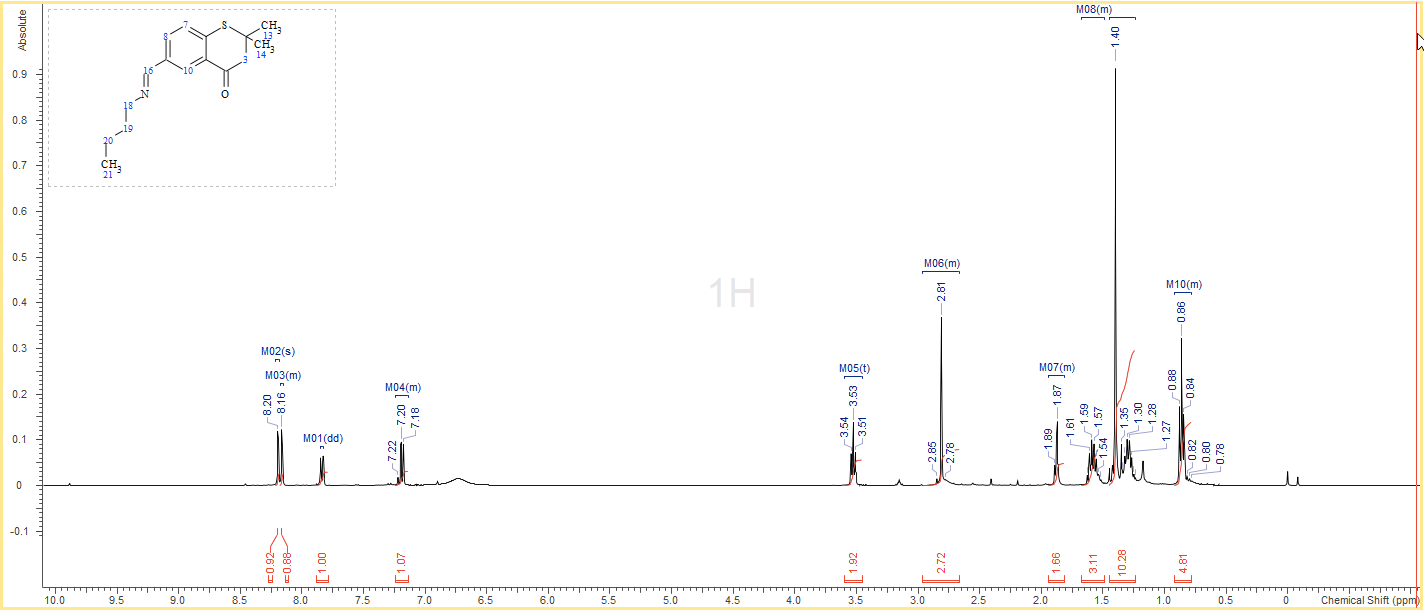
^

^13^C-NMR (101 MHz, CDCl_3_) spectrum of compound **22a**

^
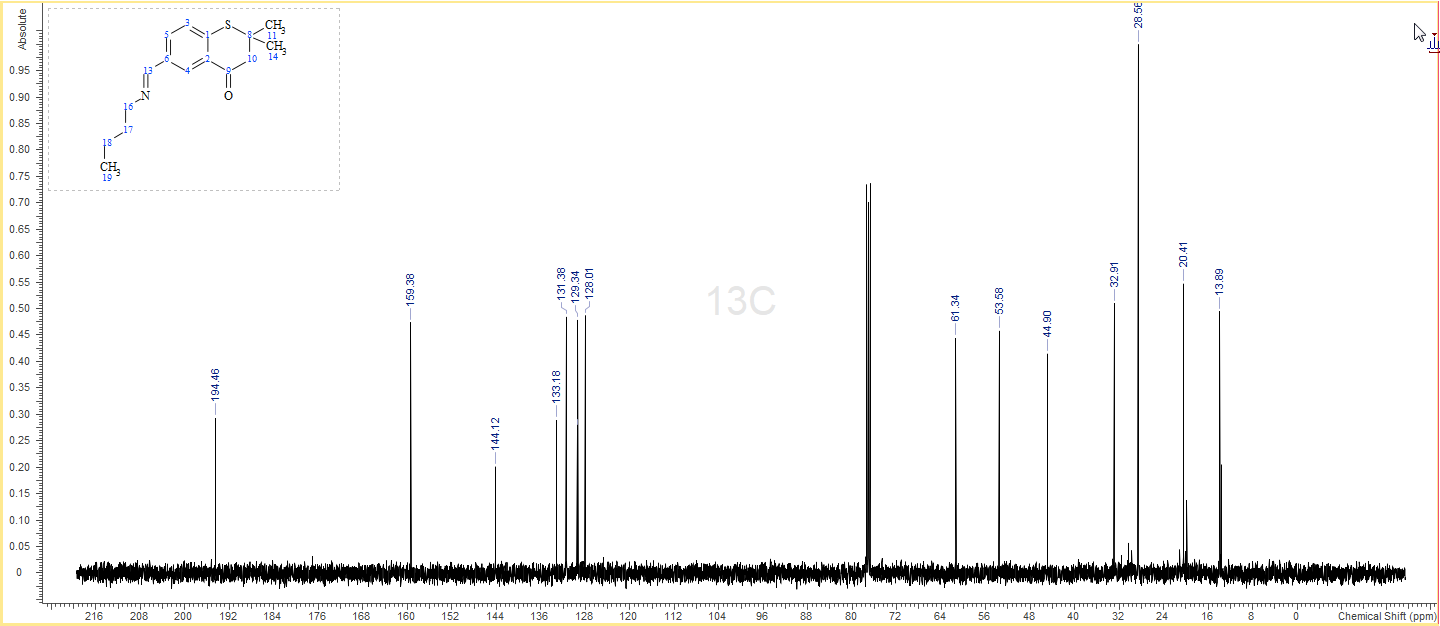
^

^1^H-NMR (400 MHz, CDCl_3_) spectrum of compound **22b**

^
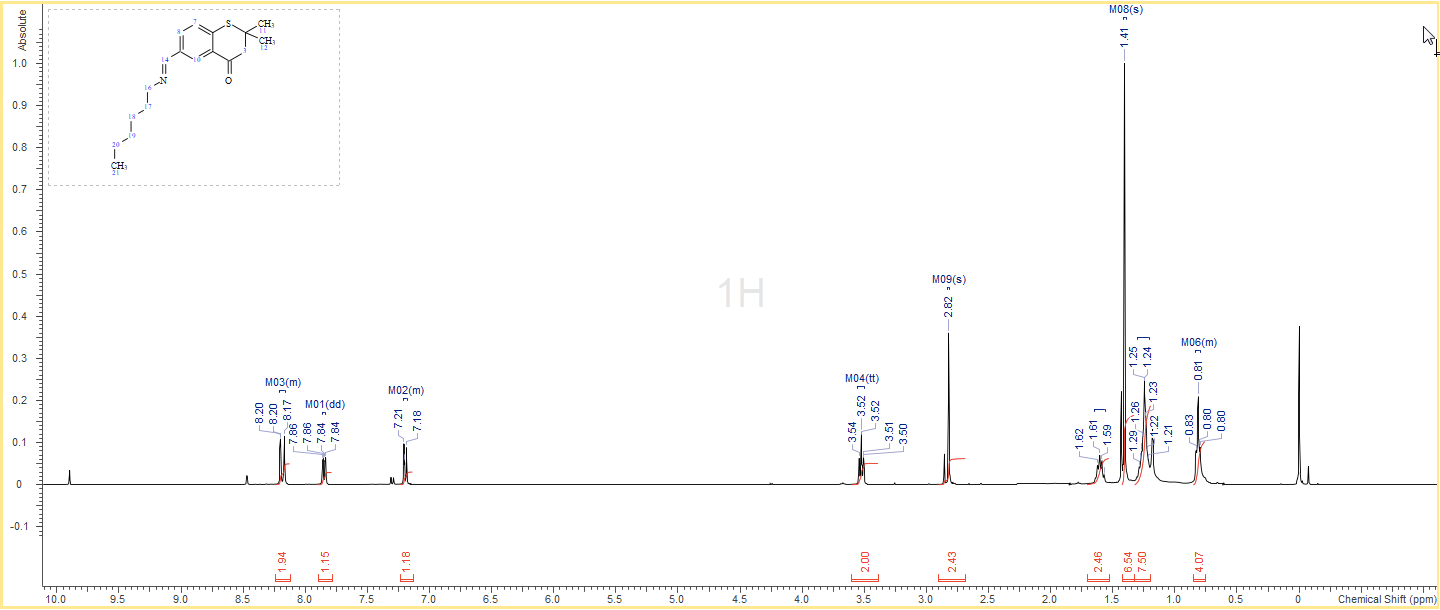
^

^13^C-NMR (101 MHz, CDCl_3_) spectrum of compound **22b**

^
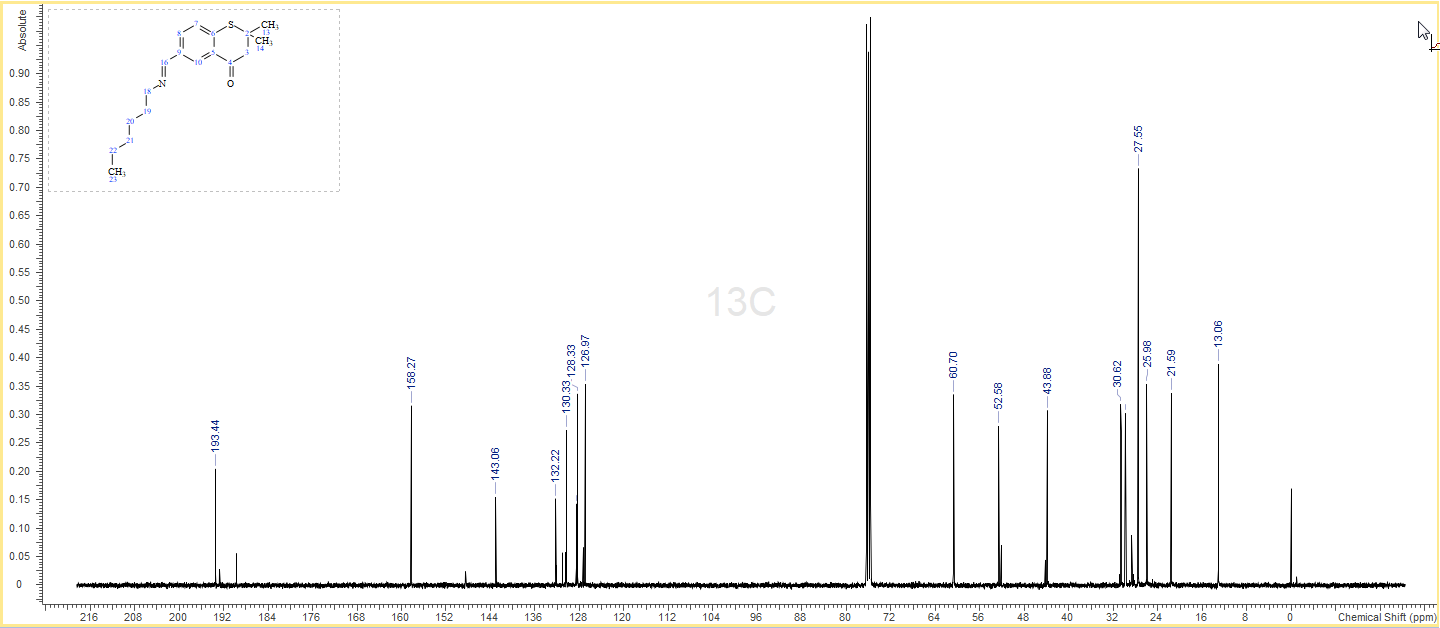
^

^1^H-NMR (400 MHz, CDCl_3_) spectrum of compound **22d**

**
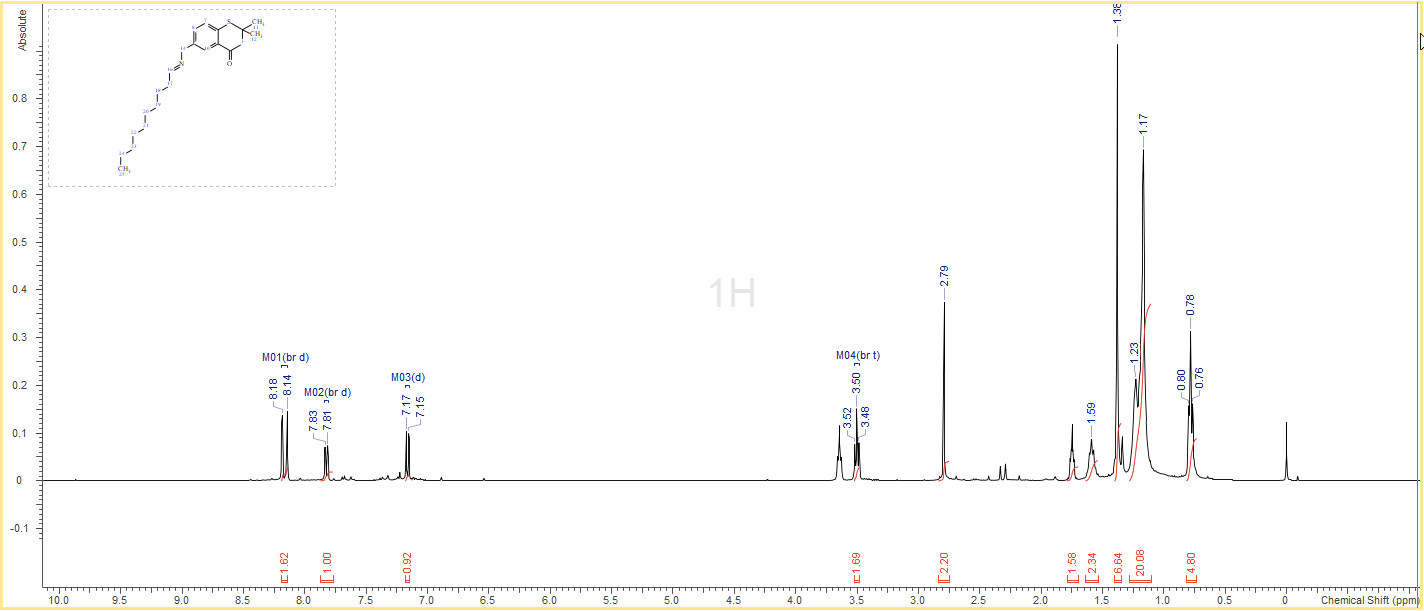
**

^13^C-NMR (101 MHz, CDCl_3_) spectrum of compound **22d**

^
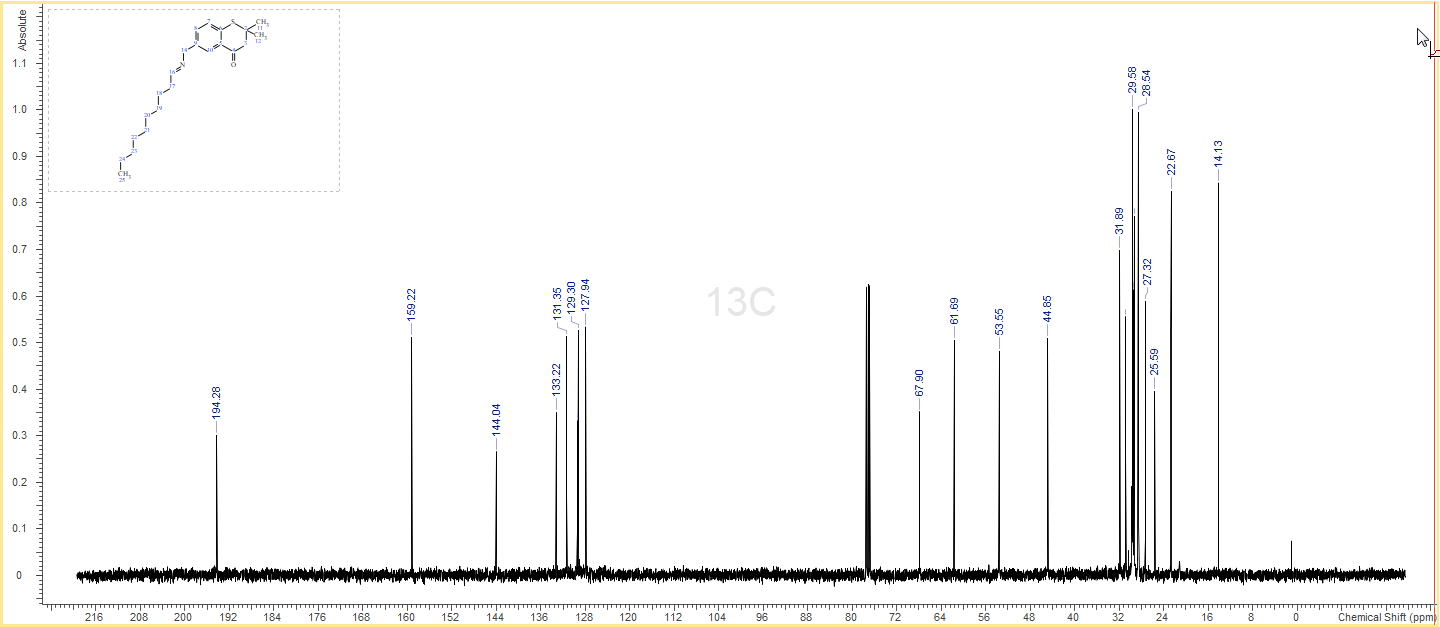
^

^1^H-NMR (400 MHz, CDCl_3_) spectrum of compound **12a**


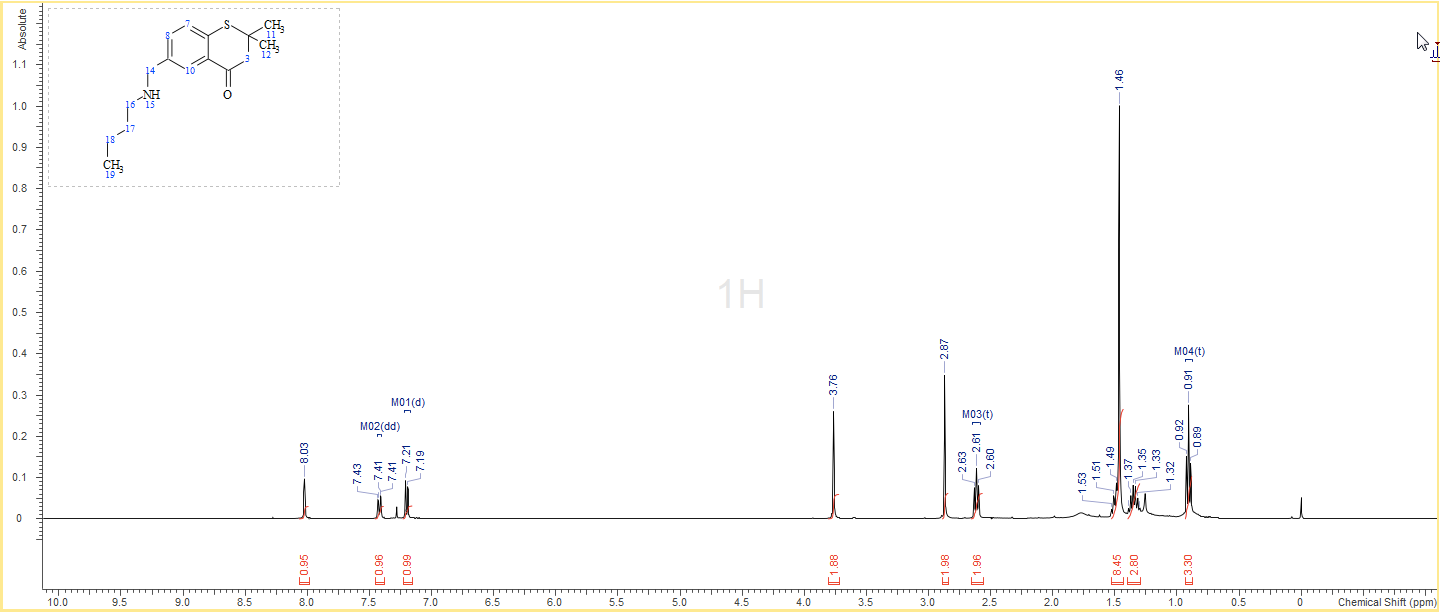


^13^C-NMR (101 MHz, CDCl_3_) spectrum of compound **12a**


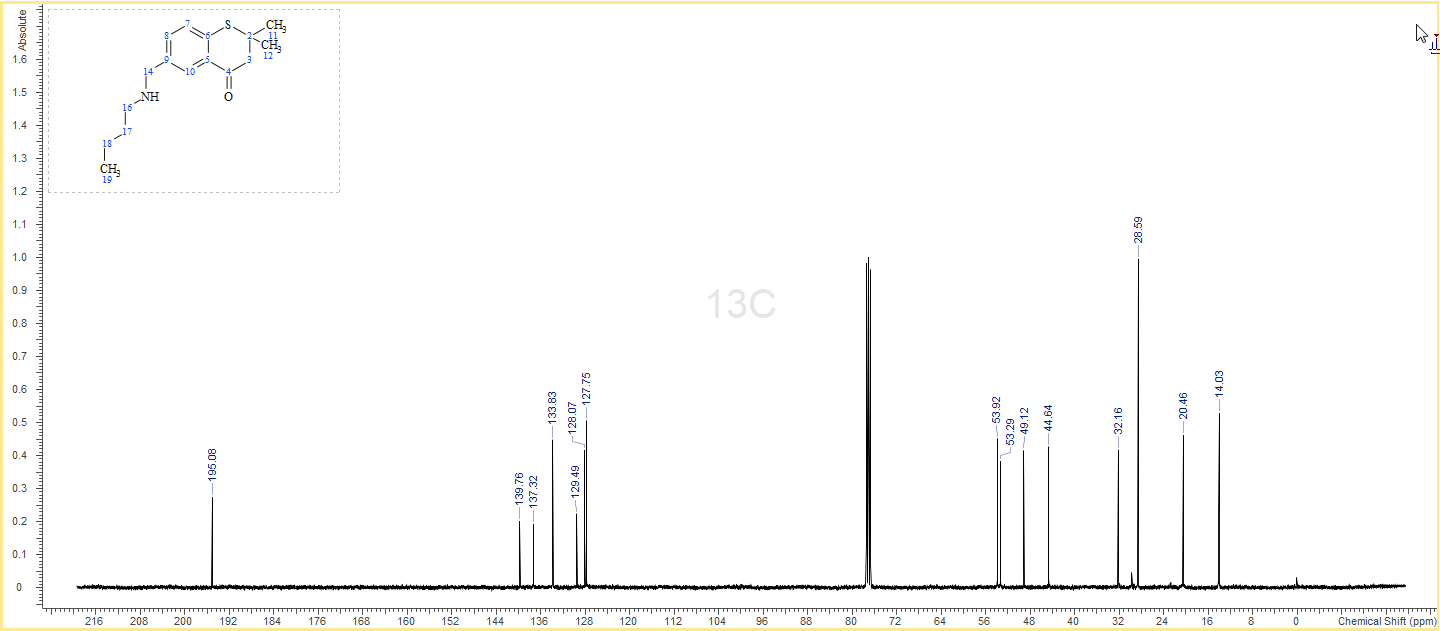


^1^H-NMR (400 MHz, CDCl_3_) spectrum of compound **12b**


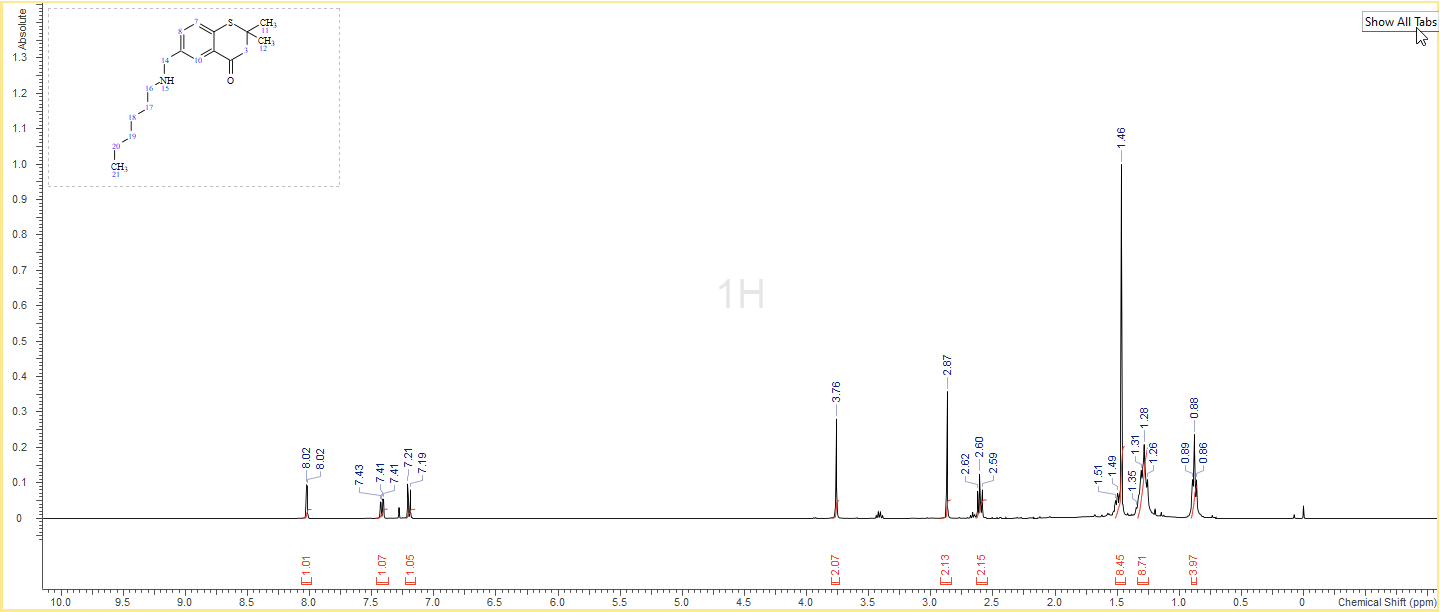


^13^C-NMR (101 MHz, CDCl_3_) spectrum of compound **12b**


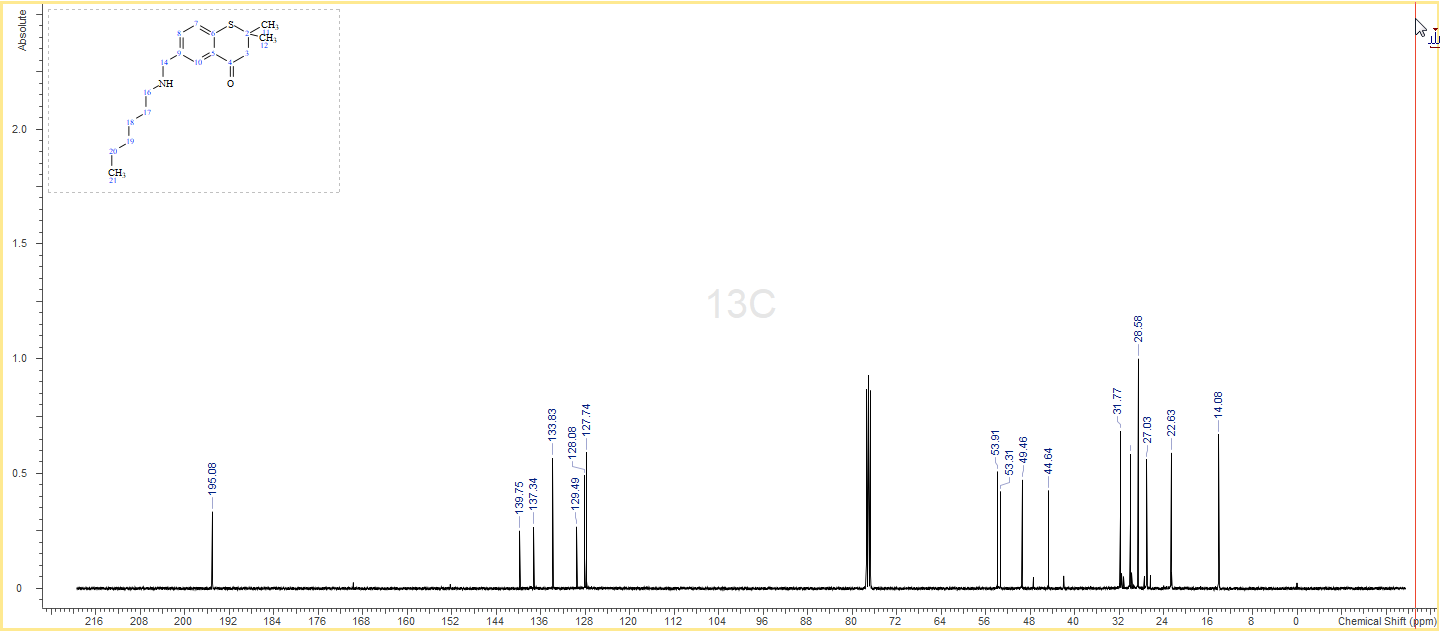


^1^H-NMR (400 MHz, CDCl_3_) spectrum of compound **12c**


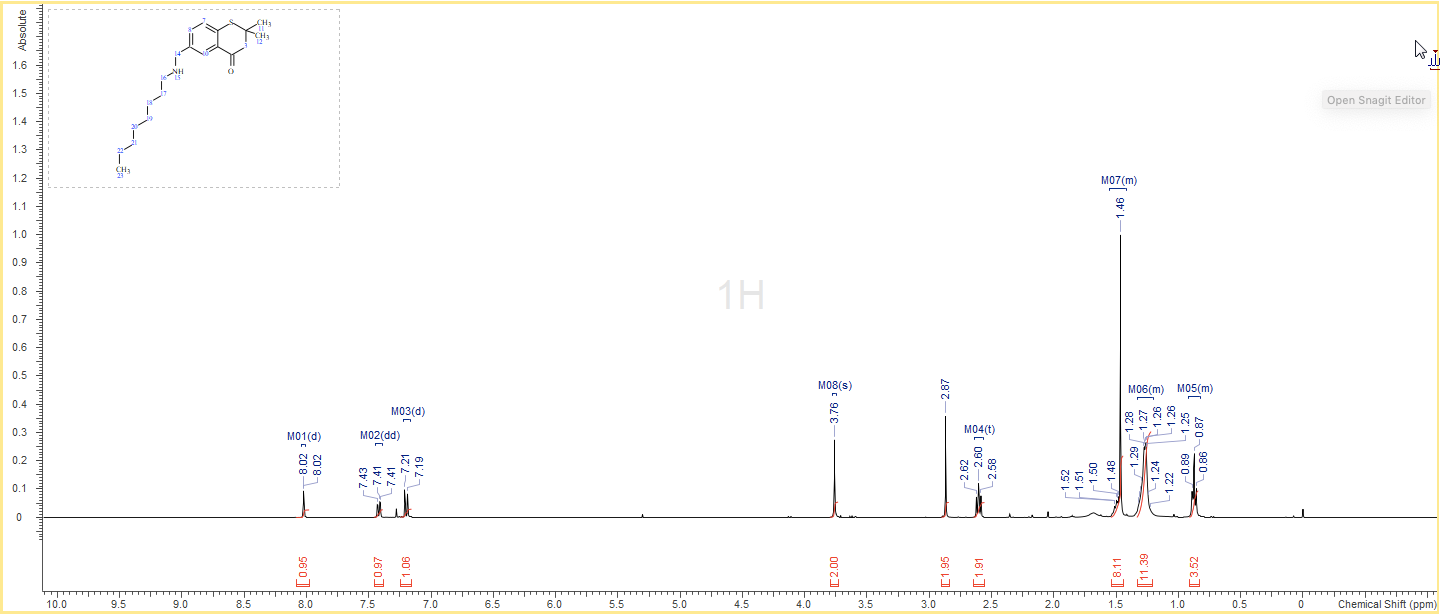


^13^C-NMR (101 MHz, CDCl_3_) spectrum of compound **12c**


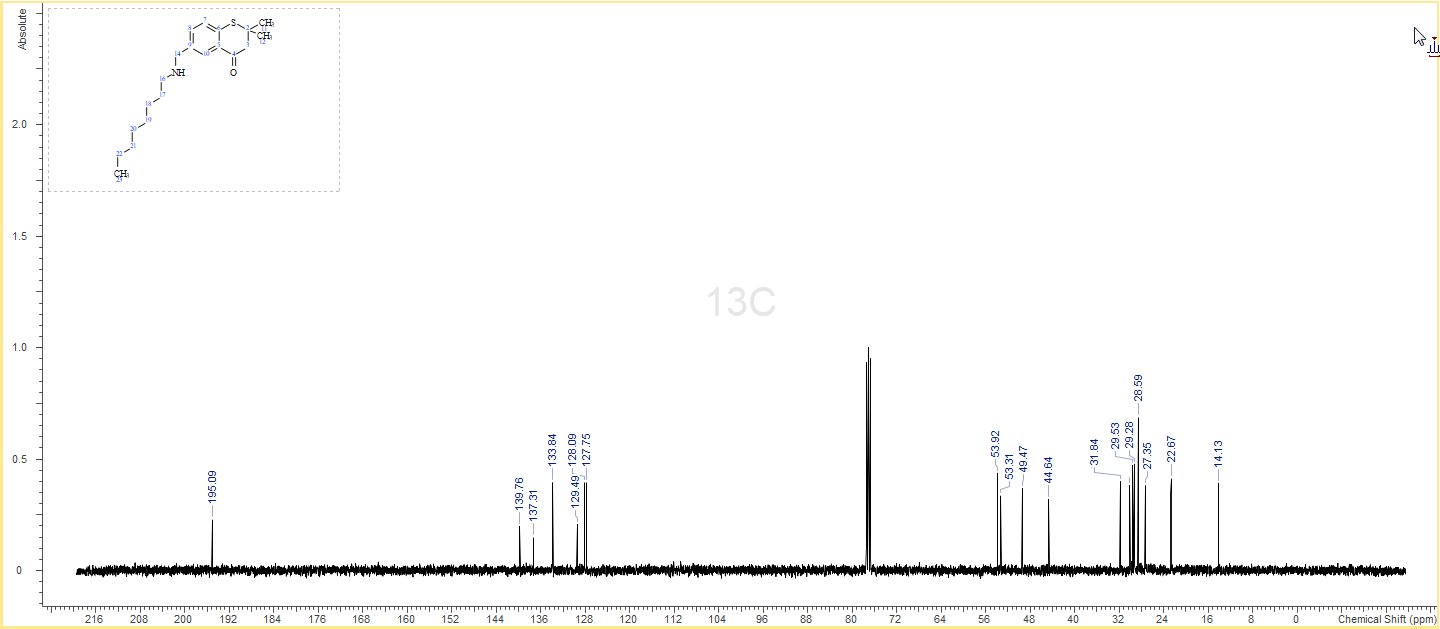


^1^H-NMR (400 MHz, CDCl_3_) spectrum of compound **12d**

**
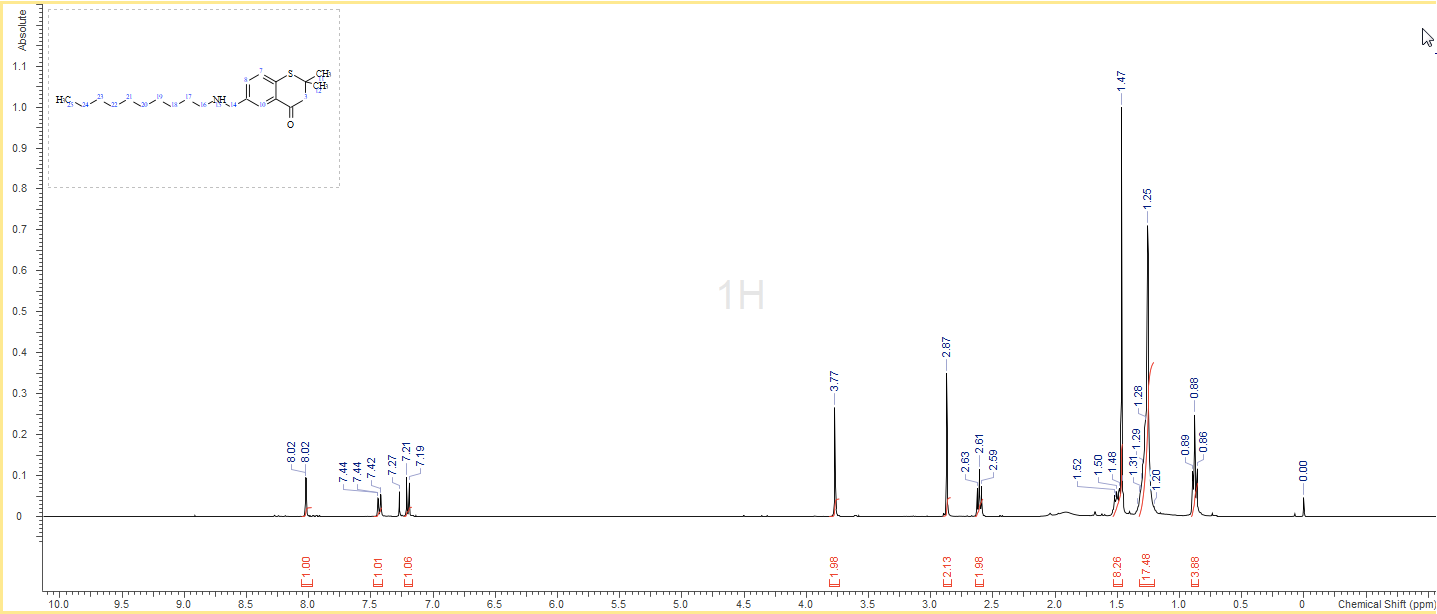
**

^13^C-NMR (101 MHz, CDCl_3_) spectrum of compound **12d**


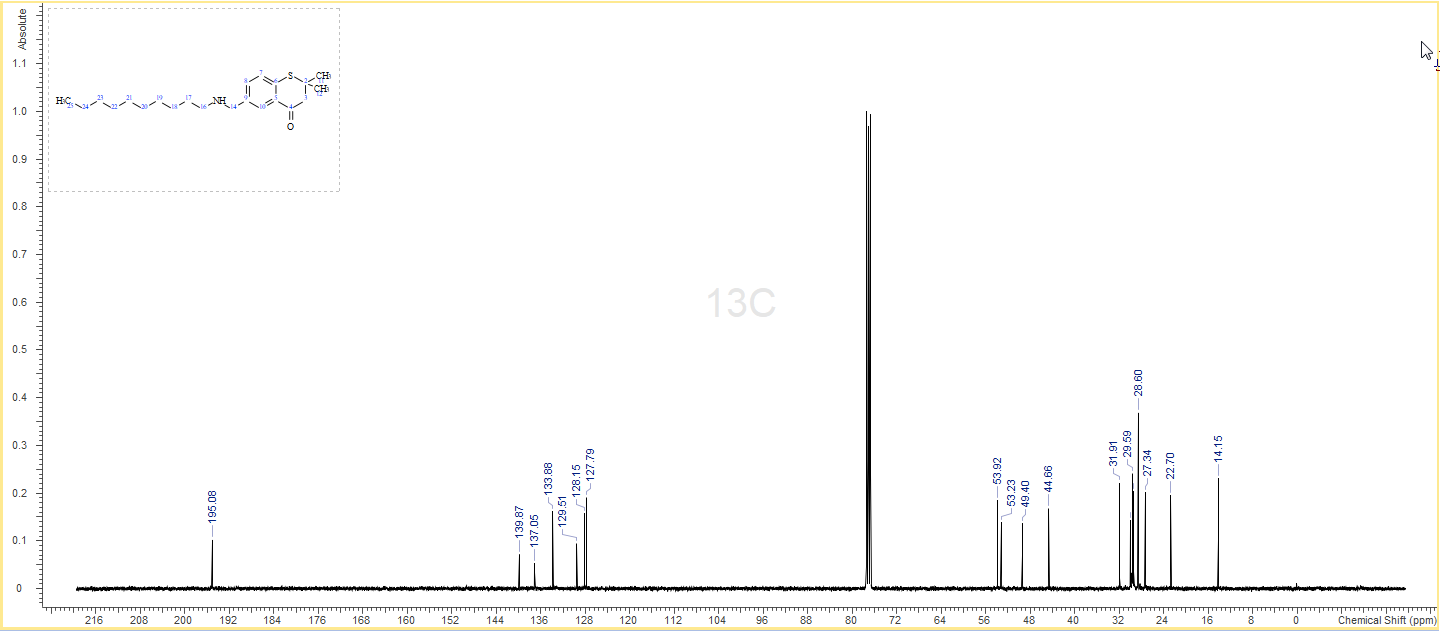


^1^H-NMR (400 MHz, CDCl_3_) spectrum of compound **23**

^
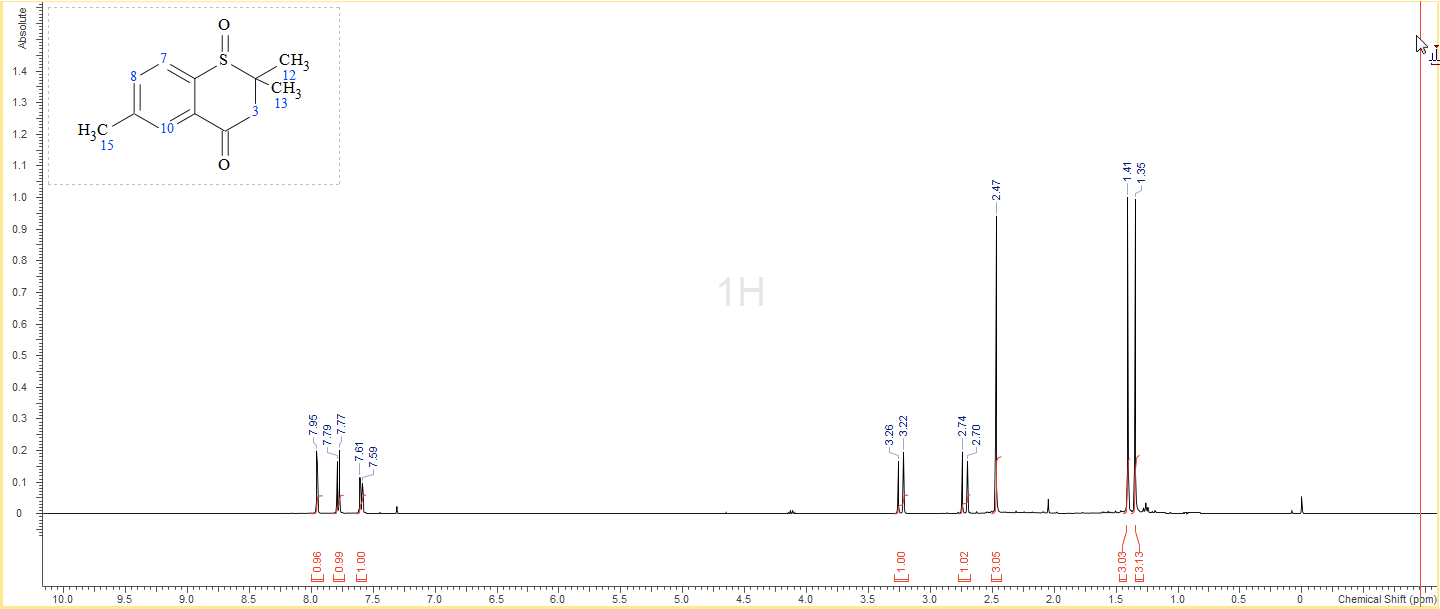
^

^1^H-NMR (400 MHz, CDCl_3_) spectrum of compound **24**


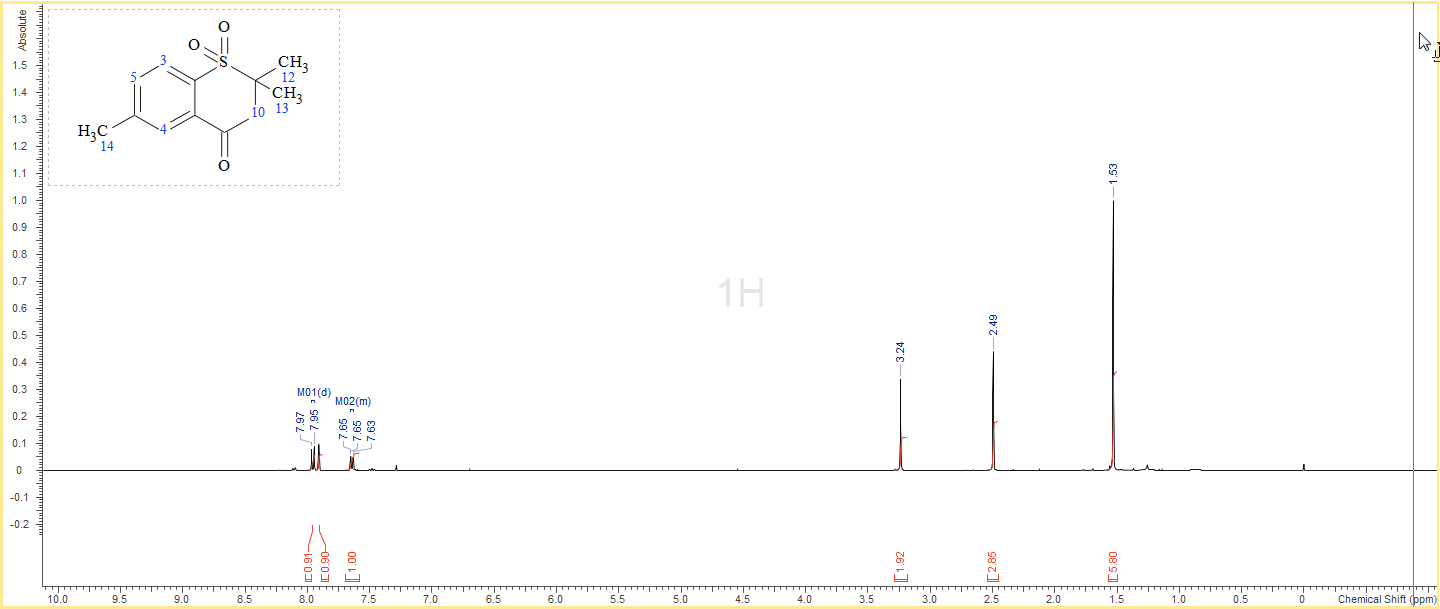


^13^C-NMR (101 MHz, CDCl_3_) spectrum of compound **24**

^
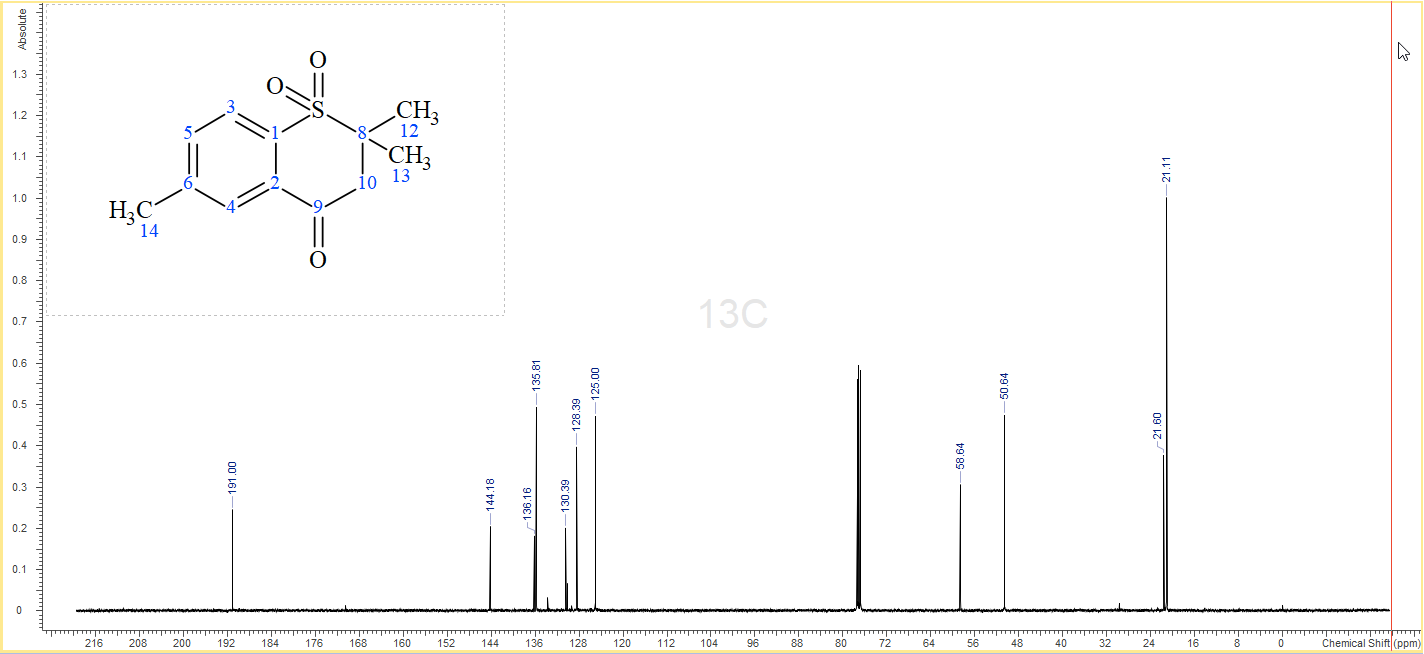
^

^1^H-NMR (400 MHz, CDCl_3_) spectrum of compound **25**


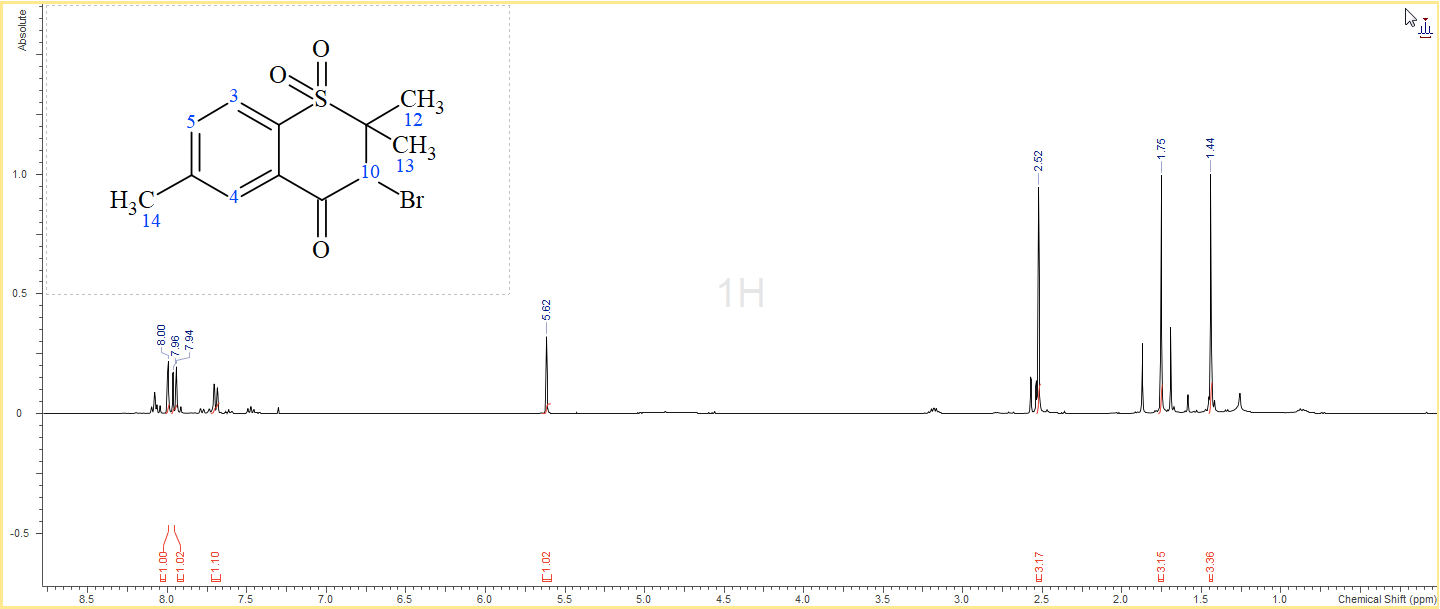


^13^C-NMR (101 MHz, CDCl_3_) spectrum of compound **25**

^
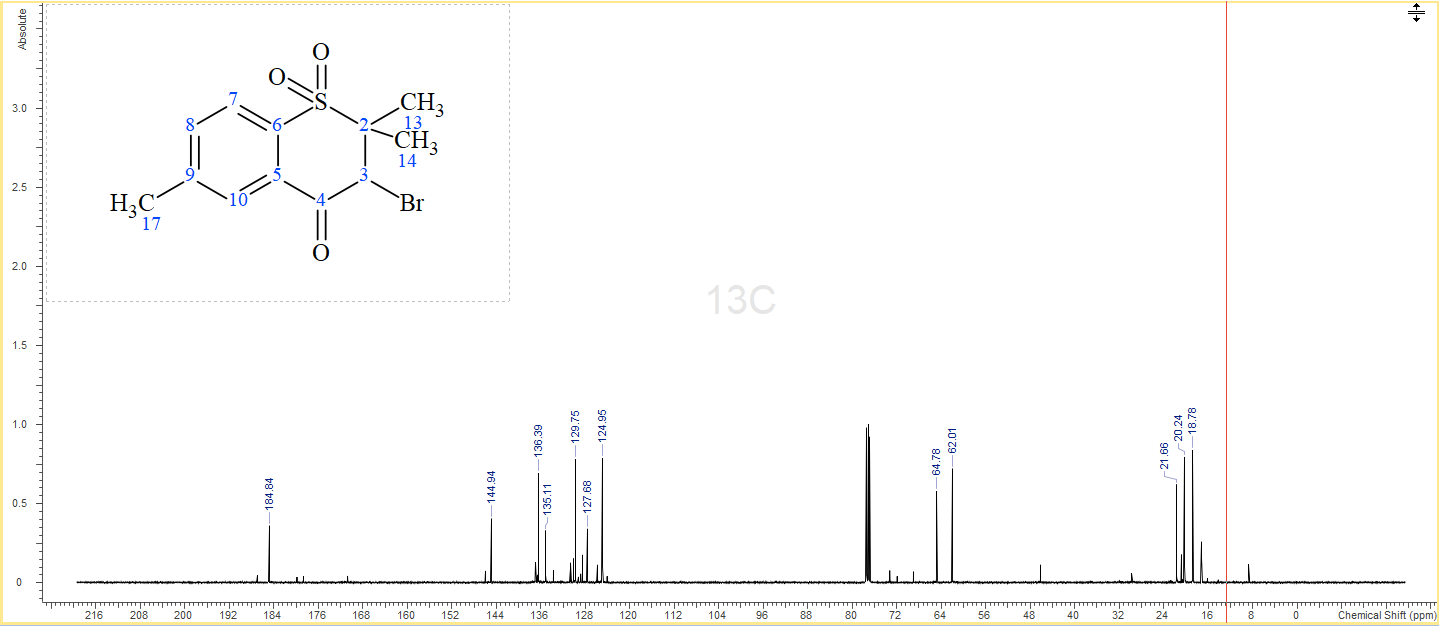
^

^1^H-NMR (400 MHz, CDCl_3_) spectrum of compound **26**


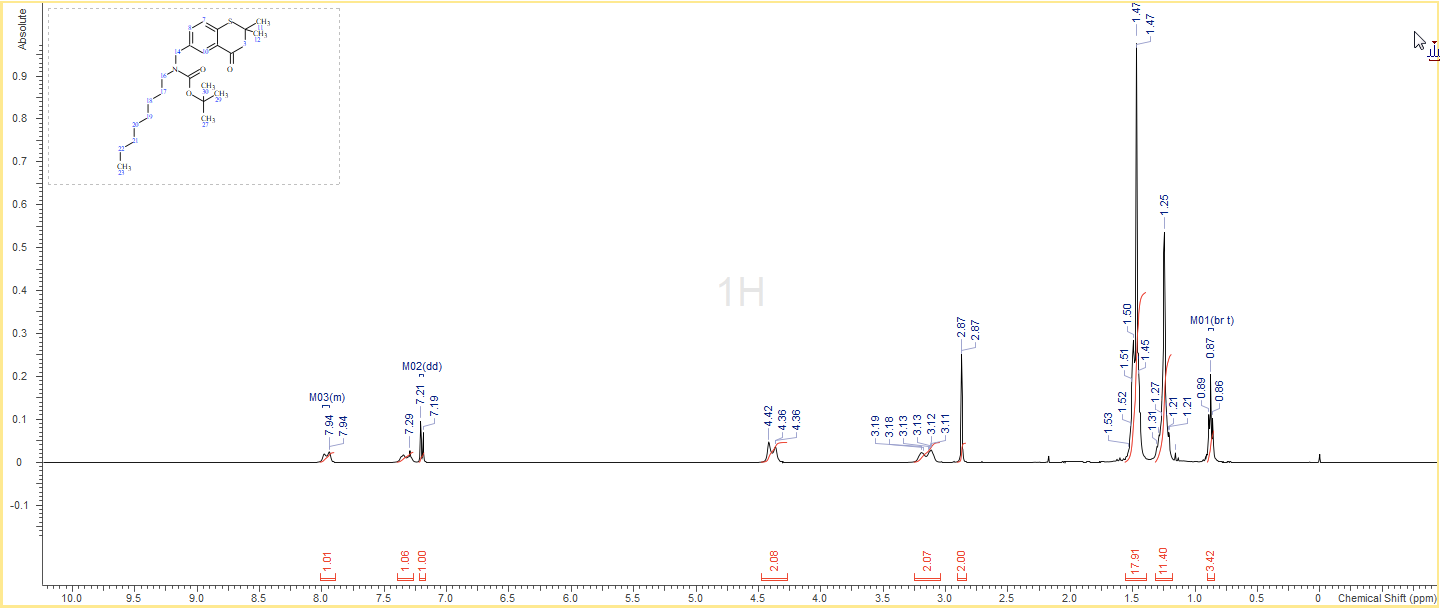


^1^H-NMR (400 MHz, CDCl_3_) spectrum of compound **27a**

^
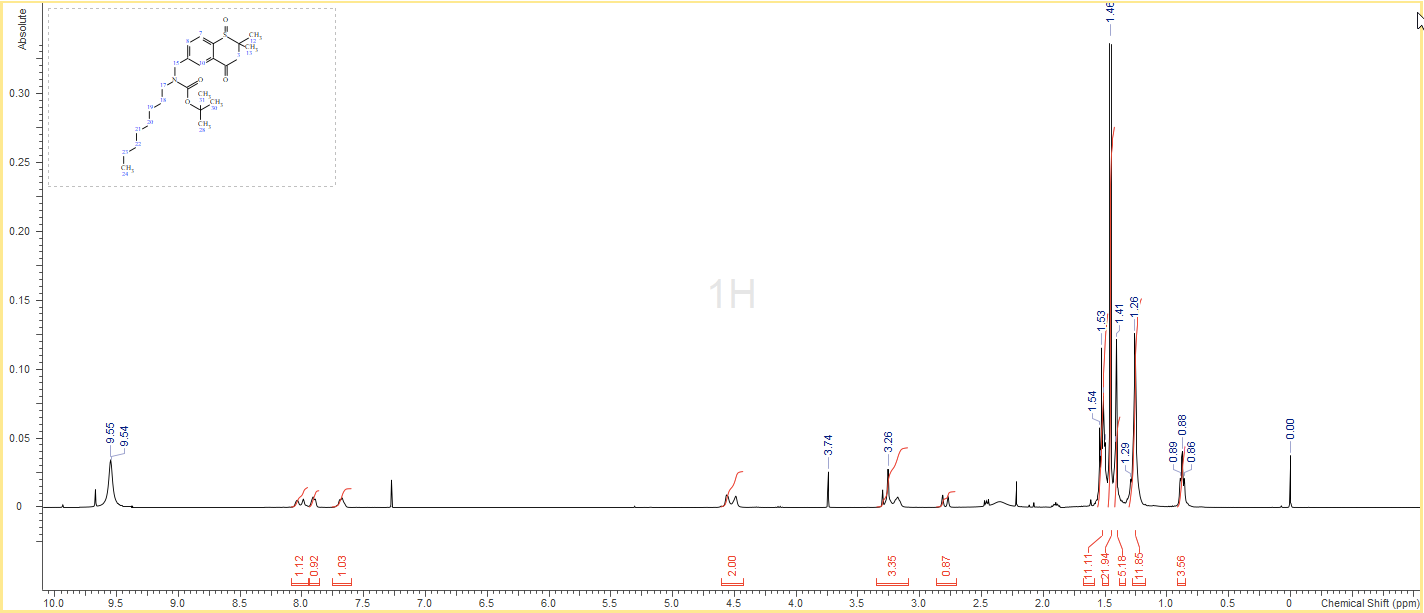
^

^1^H-NMR (400 MHz, CDCl_3_) spectrum of compound **27b**

^
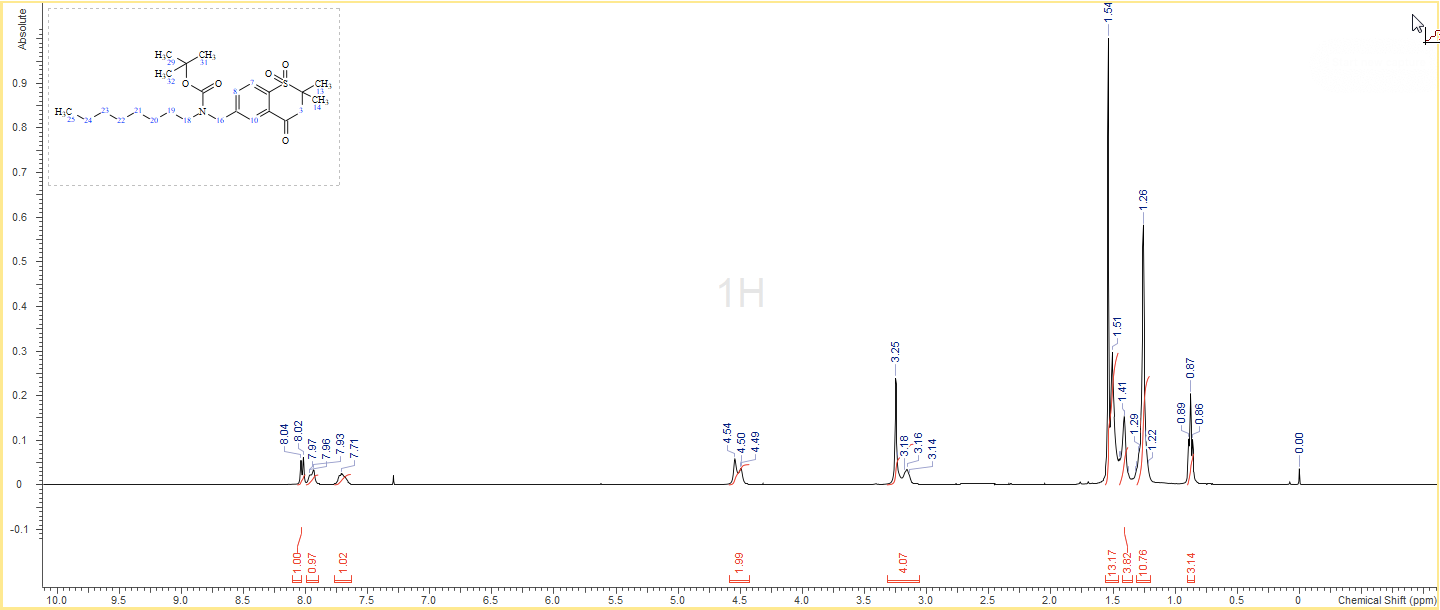
^

^1^H-NMR (400 MHz, CDCl_3_) spectrum of compound **13**

^
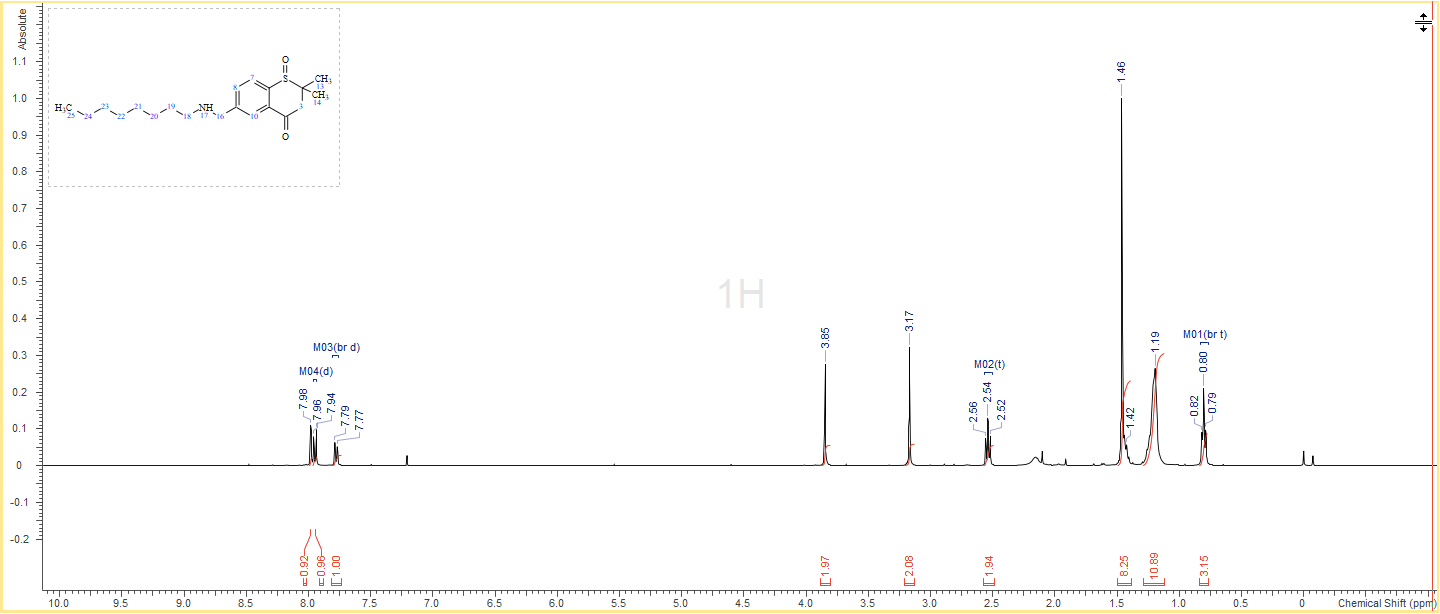
^

^13^C-NMR (101 MHz, CDCl_3_) spectrum of compound **13**

^
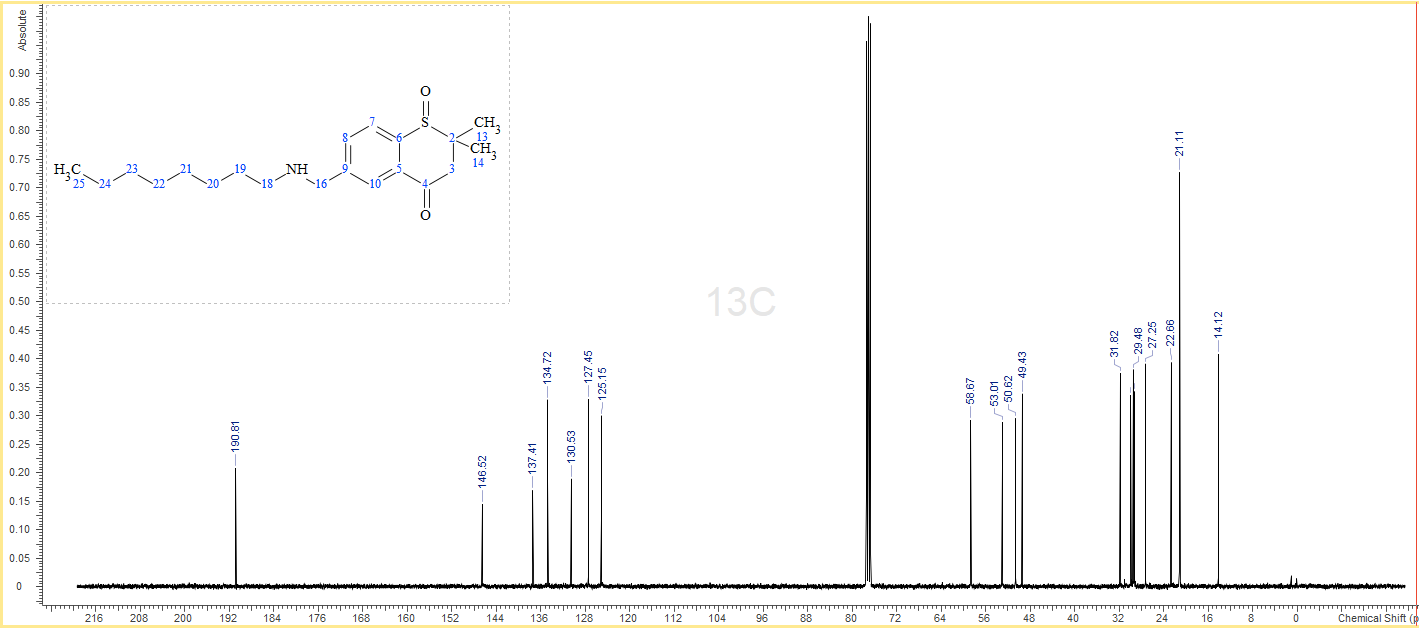
^

^1^H-NMR (400 MHz, CDCl_3_) spectrum of compound **14**


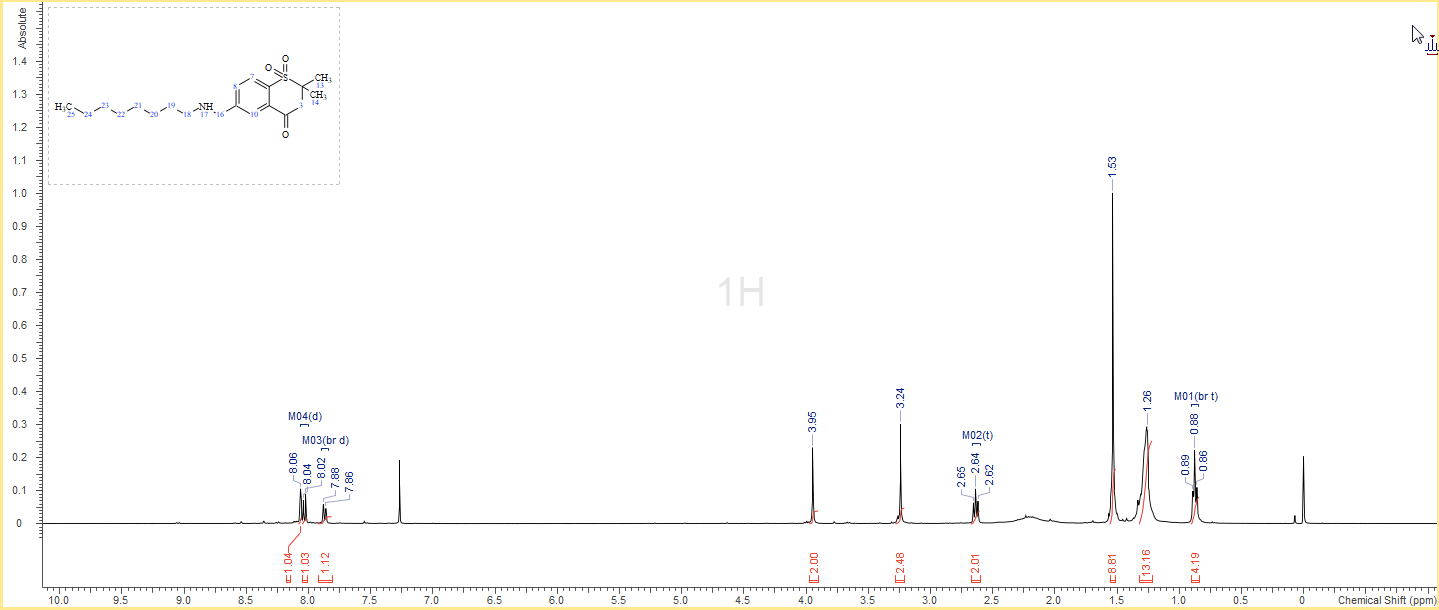


^13^C-NMR (101 MHz, CDCl_3_) spectrum of compound **14**

**
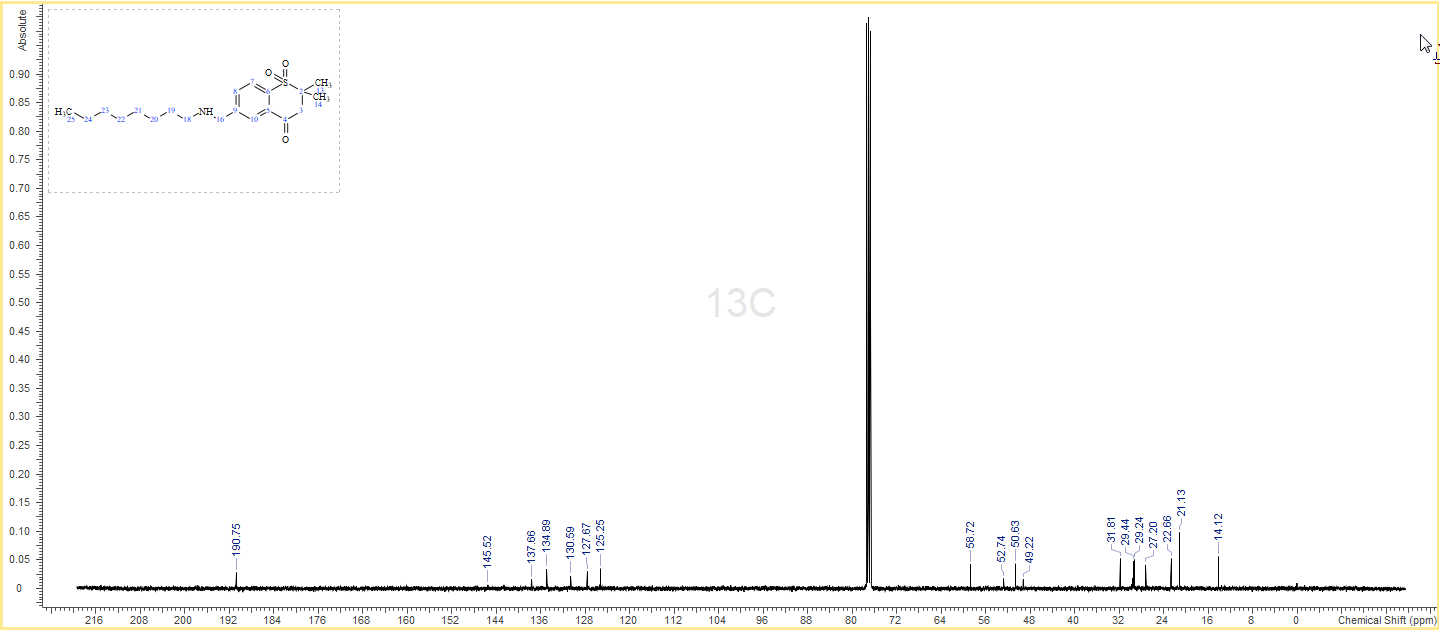
**
